# ﻿Revision of the genus *Arthrotus* Motschulsky, 1858 (Coleoptera, Chrysomelidae, Galerucinae) of Taiwan, with notes on color polymorphism

**DOI:** 10.3897/zookeys.1091.79486

**Published:** 2022-04-01

**Authors:** Chi-Feng Lee

**Affiliations:** 1 Applied Zoology Division, Taiwan Agricultural Research Institute, Taichung 413, Taiwan Taiwan Agricultural Research Institute Taichung Taiwan

**Keywords:** *Dercetina*, food plant, leaf beetles, new species, new synonym, nomenclature, taxonomy

## Abstract

Seven species of *Arthrotus* are recognized and redescribed: *A.abdominalis* (Chûjô, 1962), *A.gressitti* Kimoto, 1969, *A.hirashimai* Kimoto, 1969, *A.fulvus* Chûjô, 1938, *A.saigusai* Kimoto, 1969, *A.tricolor* (Chûjô, 1965), and *A.testaceus* Gressitt & Kimoto, 1963. Also, two new species are described: *A.yuae***sp. nov.** and *A.yangi***sp. nov.** Three new synonyms are proposed: *Proegmenataiwana* Takizawa, 1978 **syn. nov.**, *Dercetinanakanei* Kimoto, 1969 **syn. nov.** and *A.shibatai* Kimoto, 1984 **syn. nov.** Lectotypes are designated for *A.fulvus* Chûjô, 1938 and *Dercestraabdominalis* Chûjô, 1962. Color polymorphism of each species is delimited base on more than 1800 specimens.

## ﻿Introduction

The genus *Arthrotus* Motschulsky, 1858 includes 48 species from the Palaearctic and Oriental regions (Nie et al. 2017). In Taiwan, Chûjô (1938, 1962, 1965) described three species: *A.fulvus* Chûjô, 1938; *A.abdominalis* (Chûjô, 1962); transferred from *Dercestra* by Kimoto (1965), and *A.tricolor* (Chûjô, 1965); transferred from *Dercetis* by Kimoto (1969). Kimoto (1969, 1984) treated six species: *A.gressitti* Kimoto, 1969, *A.hirashimai* Kimoto, 1969, *A.saigusai* Kimoto, 1969, *A.nakanei* (Kimoto, 1969; transferred from *Dercetina* by Lee and Bezdĕk (2013)), *A.testaceus* Gressitt & Kimoto, 1963 (newly recorded by Kimoto (1969)), and *A.shibatai*, Kimoto, 1984. Takizawa (1978) described one species as *Proegmenataiwana* which was transferred to *Arthrotus* by Kimoto (1996). In total, ten species have been recorded or described from Taiwan previously (Table 1).

**Table 1.** Taxonomic works on species of *Arthrotus* of Taiwan.

Reference	New species, new records, or nomenclatural acts
Chûjô 1938	*A.fulvus*
Chûjô 1962	*Dercestraabdominalis*
Chûjô 1965	*Dercetistricolor*
Kimoto 1965	*A.abdominalis* (Chûjô, 1962) comb. nov. (transferred from *Dercestra*)
Kimoto 1969	*A.gressitti*, *A.hirashimai*, *A.saigusai*, *Dercetinanakanei*; *A.testaceus* Gressitti & Kimoto, 1963 (new record); *A.tricolor* (Chûjô, 1965) comb. nov. (transferred from *Dercetis*)
Takizawa 1978	*Proegmenataiwana*
Kimoto 1984	*A.shibatai*
Kimoto 1996	*A.taiwanus* (Takizawa, 1978) comb. nov. (transferred from *Proegmena*)
Lee and Bezdĕk 2013	*A.nakanei* (Kimoto, 1969) comb. nov. (transferred from *Dercetina*)

*Arthrotus* Motschulsky is similar to *Dercetina* Gressitt & Kimoto, 1963, with the following combination of shared characters: pronotum usually with one pair of lateral depressions, basal margin entirely marginate; closed anterior coxal cavity; elytra without setae; tibia of hind leg without one apical spine; first tarsomere of hind leg usually shorter than or subequal to combination of the rest; tarsal claws appendiculate. These genera differ from each other only by the structure of the male antennae: antennomere III is approximately twice as long as antennomere II in *Dercetina*, while antennomeres II and III are subequal in length in *Arthrotus*.

Most members of *Arthrotus* have similar shapes of male aedeagi and great color variation. Species boundaries are hard to determine without sufficient material. Fortunately, adults are collected easily by sweeping. More than 1800 specimens are available for study thanks to collecting efforts by members of the Taiwan Chrysomelid Research Team (TCRT), and borrowed material from several museums (see below).

## ﻿Materials and methods

For taxonomic study, the abdomens of adults were separated from the forebodies and boiled in 10% KOH solution, followed by washing in distilled water to prepare genitalia for illustrations. The genitalia were then dissected from the abdomens, mounted on slides in glycerin, and studied and drawn using a Leica M165 stereomicroscope. For detailed examination, a Nikon ECLIPSE 50i microscope was used.

At least three sex pairs from each species were examined to delimit variability of diagnostic characters. For species collected from more than one locality or with color variations, at least one sex pair of specimens from each locality and color morph was examined. Length was measured from the anterior margin of the eye to the elytral apex, and width at the greatest width of the elytra.

Specimens studied herein are deposited at the following institutes and collections:

**BPBM**Bernice P. Bishop Museum, Hawaii, USA [James Boone];

**CAS**California Academy of Sciences, California, USA [David H. Kavanaugh];

**HTC** Haruo Takizawa private collection;

**KMNH**Kitakyushu Museum of Natural History and Human History, Kitakyushu, Japan [Yûsuke Minoshima];

**KUEC**Faculty of Agriculture, Kyushu University, Fukuoka, Japan [Osamu Tadauchi];

**NMNS** National Museum of Natural Science, Taichung, Taiwan [Jing-Fu Tsai];

**OMNH**Osaka Museum of Natural History, Osaka, Japan [Shigehiko Shiyake];

**TARI**Applied Zoology Division, Taiwan Agricultural Research Institute, Taichung, Taiwan.

Precise label data are cited for all type specimens of described species; a double slash (//) divides the data on different labels and a single slash (/) divides the data in different rows. Other comments and remarks are in square brackets: [p] – preceding data are printed, [h] – preceding data are handwritten, [w] – white label, [y] – yellow label, [b] – blue label, and [r] – red label.

## ﻿Taxonomy

### ﻿ Arthrotus abdominalis

Taxon classificationAnimaliaColeopteraChrysomelidae

(Chûjô, 1962)

152F08FA-EDFB-5E68-87FA-EB0B59D3C348

Figs 1A–C , 2 , 3

Dercetis metallica : Chûjô 1935: 169 (nec Weise 1922); misidentification (Chûjô 1962)
Dercestra abdominalis Chûjô, 1962: 166; Chûjô 1965: 93 (additional records).
Arthrotus abdominalis : Kimoto, 1965 (transferred from Decesta); Kimoto 1969: 59 (additional records); Kimoto 1986: 58 (additional records); Kimoto 1989: 259 (additional records); Kimoto 1991: 16 (additional records).

#### Type series.

***Lectotype*** ♂ (TARI, here designated): “Hatonosawa (Chiuchihtse, 鳩之澤) / Mt. Taiheizan / 23.vii.1940 / FORMOSA / Col. M. CHUJO [p, w] // 2634 [p, w]”. Paralectotypes. 2♂, 1♀ (TARI), same holotype but with “2635–2637 [p, w]” respectively;1♀ (TARI): “RAISYA (in Chaochou, 潮州) / 30-VIII-1927 / J. Sonan [p, w] // 1910 [p, w]”; 2♀: “Formosa / Karenko (= Hualien, 花蓮), -19. / VII 20-VIII 4 / T. Okuni. [p, w] // 1911, 2155 [respectively, p, w]”; 1♂ (head detached, glued on another card) (TARI): “ (明治) 41[h]年[p]4[h]月[p]20[h]日[p] (= 20.IV.1908) / Kuaru (= Kueitzuchiao, 龜子角) [h, w, in Japanese] // Nitobe [p, w] // 2150 [p, w]”; 1♂ (TARI): “18/IV 1910 / Kammon [h] (in Hualien, 花蓮) / Col. I. Nitobe [p, w] // 2151 [p, w]”; 1♂, 2♀ (TARI): “Formosa / Musha (= Wushe, 霧社), 1919. / V 18 – VI 15. / T. Okuni [p, w] // 2152–2154 [respectively, p, w]”; 1♂, 1♀ (TARI, with heads lost): “Takeyama (= Chushan, 竹山) / 17.IV.1928 / Coll. R. Takahashi [p, w] // 2156, 2157 [respectively, p, w]”; 1♀ (TARI): “KUSKUS (typed as Kusukusu in the original description, = Kaoshih, 高士) / 18.III.1930 / Col. T. Shiraki (typed wrongly as “R. Takahashi”) [p, w] // 2158 [p, w]”; 1♀ (TARI): “Taihorin (= Talin, 大林) / Formosa / H. Sauter, 1911 [p, w] // 7.VII. [p, w] // Dercetes [sic!] / metallica Weise [h] / DET. M. CHUJO [p, b] // 2607 [p, w]”; 1♀ (TARI): “Kankau (Koshun (= Henchun, 恆春)) / Formosa / H. Sauter V. 1912 [p, w] // 7.IV. [p, w] // Dercetes [sic!] / metallica Weise [h] / DET. M. CHUJO [p, b] // 2608 [p, w]”; 1♂ (TARI): “Kankau (Koshun (= Henchun, 恆春)) / Formosa / H. Sauter V.(22) (indicated in the original description). 1912 [p, w] // Dercetes [sic!] / metallica Weise [h] / DET. M. CHUJO [p, b] // 2609 [p, w]”; 1♂ (TARI): “Kosempo (= Chiasien, 甲仙) / Formosa / H. Sauter 1912 [p, w] // 22.V. [p, w] // Dercetes [sic!] / metallica Weise [h] / DET. M. CHUJO [p, b] // 2610 [p, w]”; 1♀ (TARI): “Shinsuiyei [sic!] (Shinsuiei = Chinshuiying, 浸水營, typed as “Sinsuiei” in the original description) / 16.III.1926 / S. Issiki [p, w] // 2611 [p, w]”; 1♂ (only head, prothorax, and part of elytra and abdomen left, TARI): “Taihoku (= Taipei, 台北) / FORMOSA / 5.VII.1941/ T. KAGEYAMA [p, w] (this card was not shown in the original description) // KuSukusu (= Kaoshih, 高士) / 25.III.1926 / S. Issiki [p, w] // 2612 [p, w]”; 1♂, 2♀ (TARI): “Urai [h] (= Wulai, 烏來) / FORMOSA [p] / 28.III.1932 [h] / COL. M. CHUJO [p, w] // 2613–2615 [respectively, p, w]”; 2♀ (TARI): “Shiigao (= Maopu, 茅圃) Chikuto (= Chutung, 竹東) / SHINCHIKU / 27–30.VI.1934 (typed wrongly as “May 27 to 30, 1934” in the original description) Col. M. CHUJO [p, w] // 2616, 2617 [respectively, p, w]”; 1♀ (TARI): “ Kuaru [h] (= Kueitzuchiao, 龜子角) / FORMOSA / 14.VI.1937 [h] / COL. M. CHUJO [p, w] // 2618 [p, w]”; 1♀ (TARI): “ Kuaru [h] (= Kueitzuchiao, 龜子角) / FORMOSA / 15.VI.1937 (typed wrongly as “1938” in the original description) [h] / COL. M. CHUJO [p, w] // 2619 [p, w]”; 2♂, 1♀ (TARI): “Rimogan [h] (= Fushan, 福山) / FORMOSA [p] / 5.IV.1940 [h] / COL. M. CHUJO [p, w] // 2620–2622 [respectively, p, w]”; 1♂, 1♀ (TARI): “Tyakon [h] (扎亞孔, near Wulai, 烏來) / FORMOSA [P] / 5.IV.1940 [h] / COL. M. CHUJO [p, w] / 2623, 2624 [respectively, p, w]”; 2♂, 5♀ (TARI): “Tipon [h] (= Chihpen, 知本) / FORMOSA [p] / 13.VI.1940 [h] / COL. M. CHUJO [p, w] // 2625–2631 [respectively, p, w]”; 1♂ (TARI): “Hakurei (= Pailing, 白嶺) / Mt. Taiheizan / FORMOSA / 16.vii.1940 / Col. M. CHUJO [p, w] // 2632 [p, w]”; 1♂ (TARI): “Hatonosawa (= Chiuchihtse, 鳩之澤) / Mt. Taiheizan / 22.vii.1940 / FORMOSA / Col. M. CHUJO [p, w] // 2633 [p, w]”; 1♂, 1♀ (TARI): “Miharasi (= Chiencheng, 見晴) / Kubayan (= Kupaiyang, 古白楊) - / Kareno-tyo / FORMOSA / 16.viii.1940 / Col. M. CHUJO [p, w] // 2638, 2639 [respectively, p, w]”; 1♀ (TARI): “TAIWAN / HASSENZAN [p] (= Pahsienshan, 八仙山) / 4.VI.1942 [h] / A. MUTUURA [p, w] // 加保台 (Kahodai = Chiapaotai) – 黎明 (Reimei = Liming) [h, on the back of the same card] / 2640 [p, w]” 5♀ (TARI): “Tipon [h] (= Chihpen, 知本) / FORMOSA [p] / 8.V.1943 [h] / COL. M. CHUJO [p, w] // 2386, 2387, 2641–2643 [respectively, p, w]”. All specimens bear two additional cards: “CO / Type [p, w, circle label with yellow letters and border] // Dercestra / abdominalis / Chûjô [h] / DET. M. CHUJO [p, w]”.

#### Other material.

A total of 316 specimens was examined (Suppl. material 1).

#### Diagnosis.

**Figure 1.**
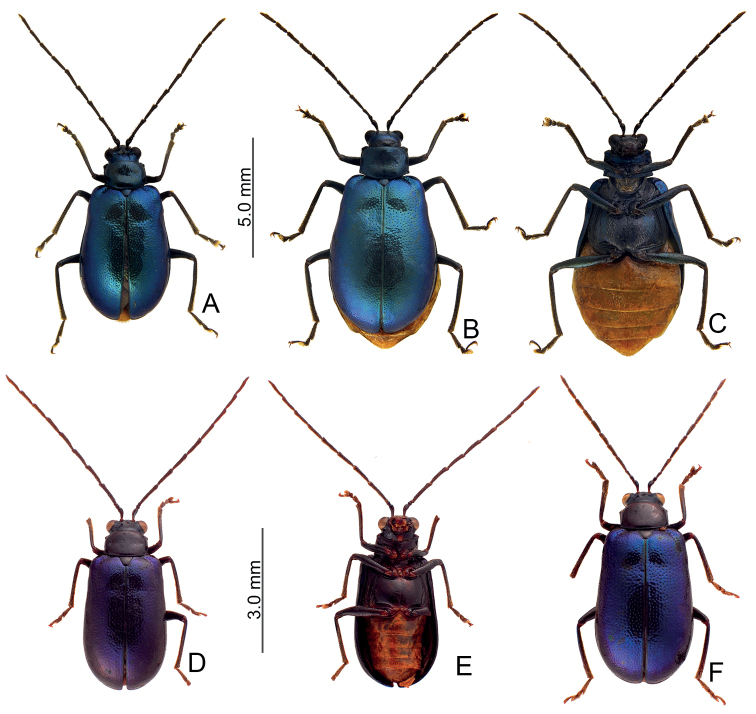
Habitus of *Arthrotusabdominalis* (Chûjô) and *A.gressitti* Kimoto **A***A.abdominalis*, male, dorsal view **B***A.abdominalis*, female, dorsal view **C***A.abdominalis*, female, ventral view **D***A.gressitti*, male, dorsal view **E***A.gressitti*, male, ventral view **F***A.gressitti*, female, dorsal view.

**Figure 2.**
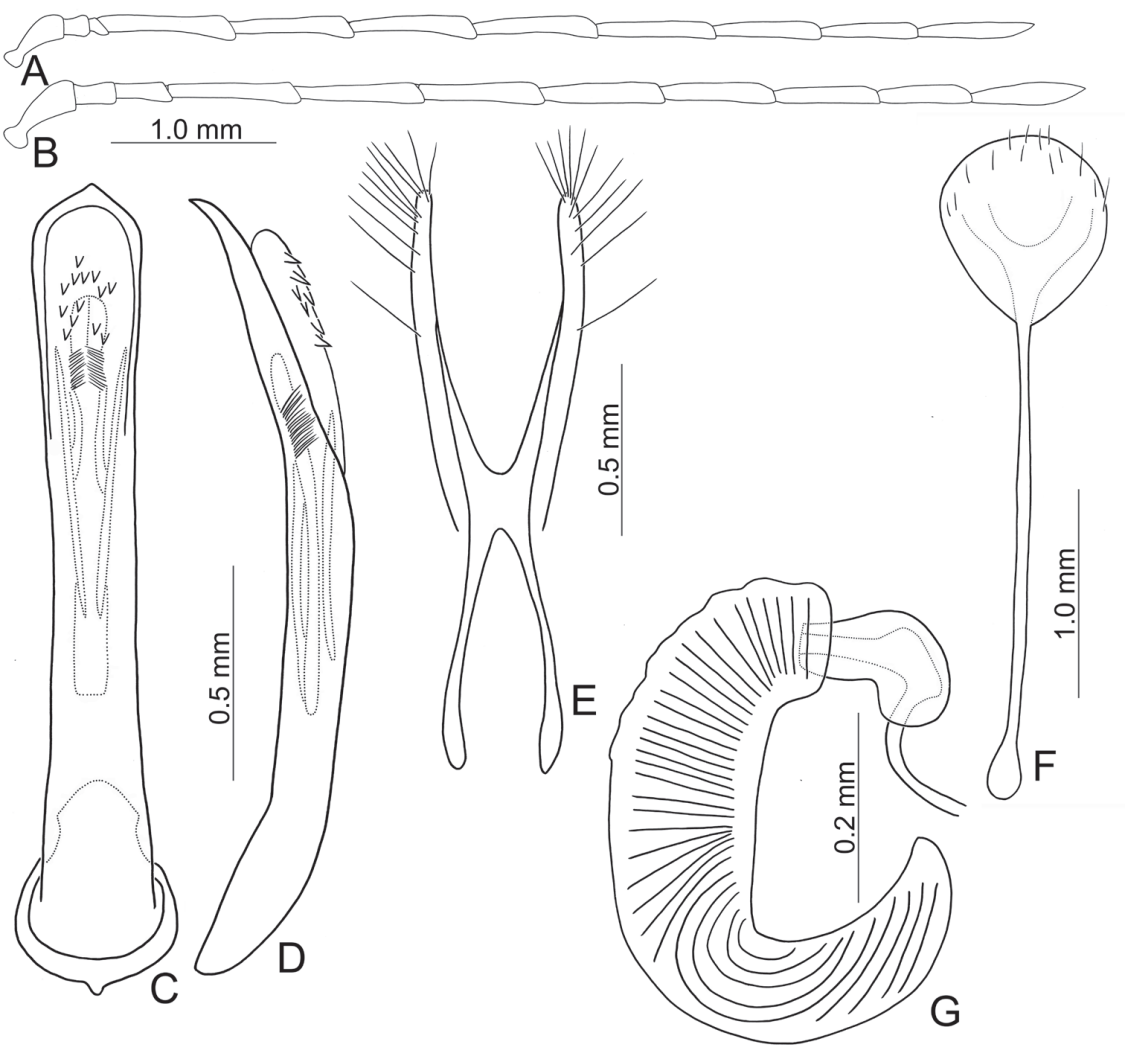
Diagnostic characters of *Arthrotusabdominalis* (Chûjô) **A** antenna, male **B** antenna, female **C** aedeagus, dorsal view **D** aedeagus, lateral view **E** gonocoxae **F** abdominal ventrite VIII **G** spermatheca.

Adults of *Arthrotusabdominalis* (Chûjô) (Fig. 1A–C) are similar to those of *A.gressitti* Kimoto (Fig. 1D–F), *A.hirashimai* Kimoto (Fig. 6A–C), and *A.yuae* sp. nov. (Fig. 6D–F) in possessing metallic blue elytra with a transverse depression at basal third (various elytra without transverse depression in other congeners), and straight lateral margins of the pronotum (rounded lateral margins of the pronotum in other congeners). Adults of this species is easily recognized by their metallic blue head, thorax, and legs (Fig. 1A–C) (black head, thorax, and legs in *A.gressitti* (Fig. 1D–F); yellowish brown head, thorax, and legs in *A.hirashimai* and *A.yuae* sp. nov. (Fig. 6)); and more slender antennae, anternnomeres V–VIII > 5.5 × longer than wide (< 5.5 × longer than wide in other congeners), tectum of aedeagus covered with stout teeth (Fig. 2C) (covered with needle-shaped setae laterally in *A.gressitti* (Fig. 4C); covered with short needle-shape laterally and stout teeth apically in *A.hirashimai* (Fig. 7C)), apex of aedeagus curved (Fig. 2D) (apex of aedeagus recurved in *A.yuae* sp. nov. (Fig. 8D)), and widely separated apices of gonocoxae (Fig. 2E) (narrowly separated apices of gonocoxae in other congeners (Figs 4E, 7E, 8E)).

#### Redescription.

Color metallic blue, antennae and legs black, abdomen yellow (Fig. 1A–C). Pronotum with transverse depression behind middle; dull, with reticulate microsculpture; with sparse, fine punctures confused with a few coarse punctures; lateral margins straight; apical and basal margins slightly concave. Elytra with rounded lateral margins, widest at apical 1/3; disc shiny, without reticulate microsculpture, and with dense, coarse punctures, and transverse depression at basal 1/3.

**Male.** Length 6.2–7.6 mm, width 3.2–4.5 mm. Antennae filiform (Fig. 2B), antennomere III modified, much shorter than II, IV–VII apically widened, length ratios of antennomeres I–XI 1.0: 0.4: 0.2: 2.0: 1.9: 1.9: 1.9: 1.8: 1.7: 1.5: 1.8, length to width ratios of antennomeres I–XI 2.7: 1.3: 0.9: 5.3: 5.8: 5.8: 6.0: 6.3: 6.2: 5.9: 7.7. Pronotum 1.5–1.6 × wider than long. Elytra 1.4–1.5 × longer than wide. Aedeagus (Fig. 2C, D) extremely slender, ~ 7.7 × longer than wide, parallel-sided, basally widened, apex narrowly rounded; tectum membranous, with scattered small, stout setae; weakly curved in lateral view; primary endophallic sclerite elongate, ~ 0.4 × as long as aedeagus, bifurcate, with a cluster of dense setae near apex, deeply bifurcate from apical 1/3 to base; a pair of dorsal sclerites present, longitudinal and slender, 0.7 × as long as primary sclerite.

**Female.** Length 7.8–9.2 mm, width 3.9–5.1 mm. Antennae similar to those of males, but antennomere III slightly longer than II in females (Fig. 2B), length ratios of antennomeres I–XI 1.0: 0.5: 0.6: 1.6: 1.6: 1.5: 1.5: 1.4: 1.3: 1.1: 1.4, length to width ratios of antennomeres I–XI 2.6: 1.9: 2.7: 5.9: 6.2: 5.9: 5.9: 5.7: 5.7: 5.0: 5.3. Pronotum 1.5 × wider than long. Elytra 1.3–1.5 × longer than wide. Ventrite VIII (Fig. 2F) weakly sclerotized laterally and apically, apical margin widely rounded, with scattered setae along lateral and apical margin, spiculum extremely slender. Receptacle of spermatheca (Fig. 2G) slightly swollen, undivided from pump; pump narrow and moderately curved, apex broadly rounded; sclerotized proximal spermathecal duct wide and short, shallowly projecting into receptaculum. Gonocoxae (Fig. 2E) narrowly connected at middle, ~ 3.4 × longer than wide, curved inwards at apical 1/3, with one long seta at apical 1/3, ten additional setae at apical areas.

#### Food plants.

Adults feed on leaves of Quercusglaucavar.glauca Thunb. (Fagaceae), *Hydrangeachinensis* Maxim. (Hydrangeaceae), *Persicariachinensis* (L.) H. Gross (Polygonaceae), *Prunuscampanulata* Maxim. (Rosaceae), *Zelkovaserrata* (Thunb.) Makino (Ulmaceae), and *Debregeasiaorientalis* C.J. Chen (Urticaceae).

#### Distribution.

**Figure 3.**
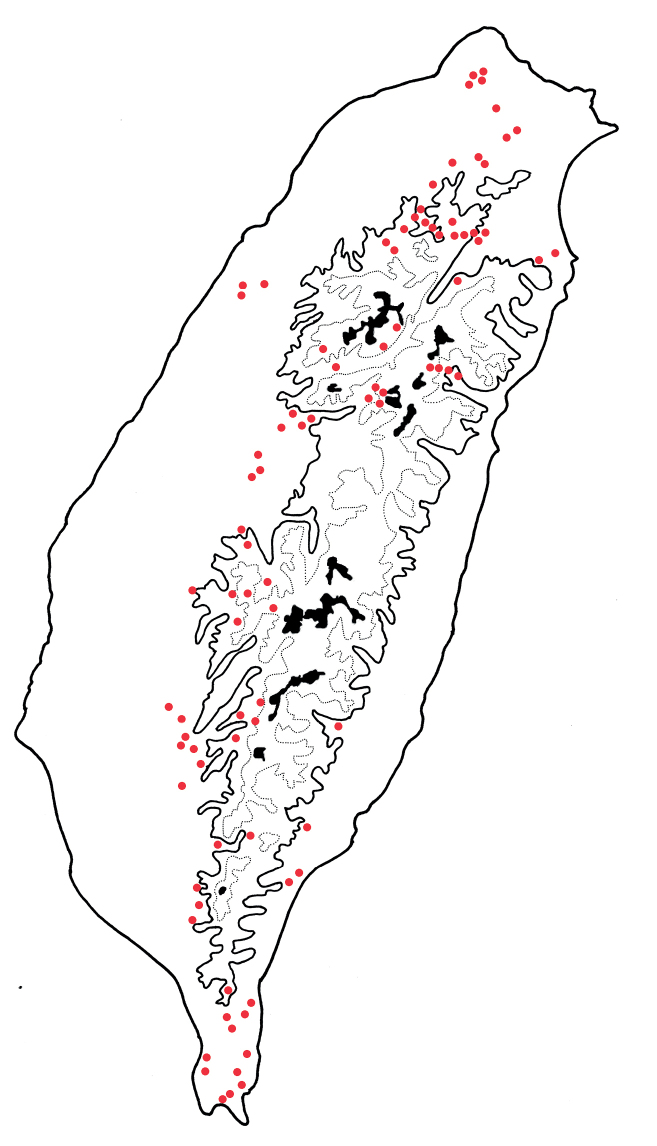
Distribution map of *Arthrotusabdominalis* (Chûjô), solid line: 1000 m, broken line: 2000 m, black areas: 3000 m.

*Arthrotusabdominalis* is a common, widespread species in lowlands (below 1500 m) of Taiwan (Fig. 3).

### ﻿ Arthrotus gressitti

Taxon classificationAnimaliaColeopteraChrysomelidae

Kimoto, 1969

D163ABD3-812E-5300-8DC9-04C4A32580F3

Figs 1D–F , 4 , 5

Arthrotus gressitti Kimoto, 1969: 61.

#### Types.

***Paratypes*.** 2♀ (BPBM): “FORMOSA / Hassenzan (= Pasienshan, 八仙山) / VI [p] 26 [h] 1934 / L. Gressitt [p, w] // L. Gressitt / Collection [p, w] // PARATOPOTYPE [p, b] // Arthrotus / gressitti / Kimoto, n. sp. [h, w]”. Holotype could be deposited at the BPBM but was not found.

#### Other material

**(*n* = 21).** 1♂, 1♀ (BPBM), same data as paratypes; 3♂, 2♀ (BPBM), same but with “VI 24 1934”; 1♀ (KMNH), same locality, 29.V.1971, leg. K. Kamiya; Nantou: 2♂, 8♀ (HTC), Habonsan (合望山 = 北東眼山), 2.VIII.1985, leg. H. Takizawa; 1♂ (HTC), Hotsu (廬山, = Lushan), 6.VII.1983, leg. H. Takizawa; 3♀ (HTC), Nanshanchi (南山溪), 31.VII.1985, leg. H. Takizawa.

#### Diagnosis.

**Figure 4.**
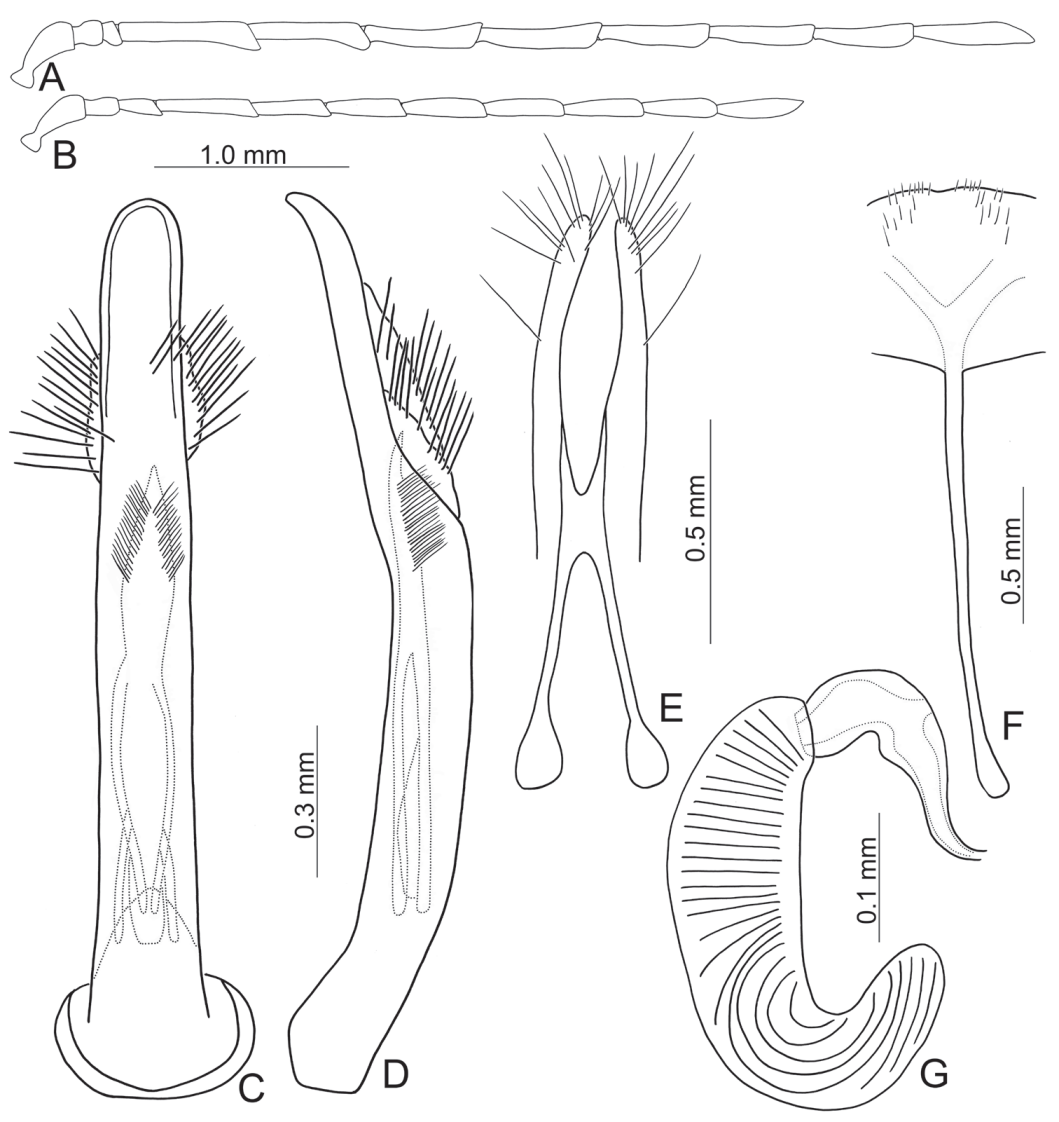
Diagnostic characters of *Arthrotusgressitti* Kimoto **A** antenna, male **B** antenna, female **C** Aedeagus, dorsal view **D** aedeagus, lateral view **E** gonocoxae **F** abdominal ventrite VIII **G** spermatheca.

Adults of *Arthrotusgressitti* Kimoto (Fig. 1D–F) are similar to those of *A.abdominalis* (Chûjô) (Fig. 1A–C), *A.hirashimai* Kimoto (Fig. 6A–C), and *A.yuae* sp. nov. (Fig. 6D–F) in possessing metallic blue elytra with a transverse depression at basal 1/3 (various elytra without transverse depression in other congeners), and straight lateral margins of the pronotum (rounded lateral margins of the pronotum in other congeners). Adults of this species is easily recognized by the black head, thorax, and legs (Fig. 1D–F) (metallic blue head, thorax, and legs in *A.abdominalis* (Fig. 1A–C); yellowish brown head, thorax, and legs in *A.hirashimai* and *A.yuae* sp. nov. (Fig. 6)); and less slender antennae, antennomeres V–VIII < 5.5 × longer than wide (> 5.5 × longer than wide in *A.abdominalis*), tectum of aedeagus covered with needle-shaped setae laterally (Fig. 4C) (covered with stout teeth in *A.abdominalis* (Fig. 2C) and *A.yuae* sp. nov. (Fig. 8C); covered with short needle-shape laterally and stout teeth apically in *A.hirashimai* (Fig. 7C)), apex of aedeagus curved (Fig. 4D) (apex of aedeagus recurved in *A.yuae* sp. nov. (Fig. 8D)), and narrowly separated apices of gonocoxae (Fig. 4E) (widely separated apices of gonocoxae in *A.abdominalis* (Fig. 2E)).

#### Redescription.

Color blackish brown, elytra metallic blue, abdomen yellow (Fig. 1D–F). Pronotum with median transverse depression; dull, with reticulate microsculpture; with sparse, fine punctures confused with a few coarse punctures; lateral margins straight, basally narrowed; apical and basal margins slightly concave. Elytra with rounded lateral margins, widest at apical 1/3; disc shiny, without reticulate microsculpture, and with dense, coarse punctures, and a feeble, transverse depression at basal 1/3.

**Male.** Length 5.9–6.6 mm, width 2.8–3.0 mm. Antennae filiform (Fig. 4A), antennomere III modified, much shorter than II, IV–VII apically widened, length ratios of antennomeres I–XI 1.0: 0.4: 0.2: 1.8: 1.6: 1.5: 1.6: 1.4: 1.4: 1.3: 1.6, length to width ratios of antennomeres I–XI 2.6: 1.1: 0.7: 4.6: 4.0: 4.2: 4.9: 4.6: 4.9: 4.6: 5.4. Pronotum 1.5 × wider than long. Elytra 1.6 × longer than wide. Aedeagus (Fig. 4C, D) extremely slender, ~ 9.6 × longer than wide, parallel-sided, slightly narrowed at apical 1/4, basally widened, apex widely rounded; tectum membranous, covered with needle-shaped setae laterally; weakly curved in lateral view, apex truncate; primary endophallic sclerite elongate, ~ 0.5 × as long as aedeagus, apex pointed, with a cluster of dense setae near apex, deeply bifurcate from middle to base; a pair of dorsal sclerites longitudinally and apically connected with primary sclerite.

**Female.** Length 7.4–7.9 mm, width 3.6 mm. Antennae (Fig. 4B) much shorter than in males, antennomere III slightly longer than II, length ratios of antennomeres I–XI 1.0: 0.4: 0.5: 1.3: 1.0: 1.0: 1.1: 1.1: 1.0: 1.0: 1.2, length to width ratios of antennomeres I–XI 3.0: 1.9: 2.2: 5.8: 4.4: 4.1: 4.6: 4.6: 4.2: 4.1: 4.8. Pronotum 1.5–1.6 × wider than long. Elytra 1.5–1.6 × longer than wide. Ventrite VIII (Fig. 4F) membranous, apically truncate with shallow median depression, with scattered long setae at sides and short setae along apical margin, spiculum extremely slender. Receptacle of spermatheca (Fig. 4G) slightly swollen, undivided from pump; pump narrow and moderately curved, apex broadly rounded; sclerotized proximal spermathecal duct wide and short, shallowly projecting into receptaculum. Gonocoxae (Fig. 4E) narrowly connected at middle, ~ 5.0 × longer than wide, curved inwards at apical 1/3, with one long seta at apical 1/3, and nine or ten additional setae at apical areas.

#### Food plants.

Unknown.

#### Distribution.

**Figure 5.**
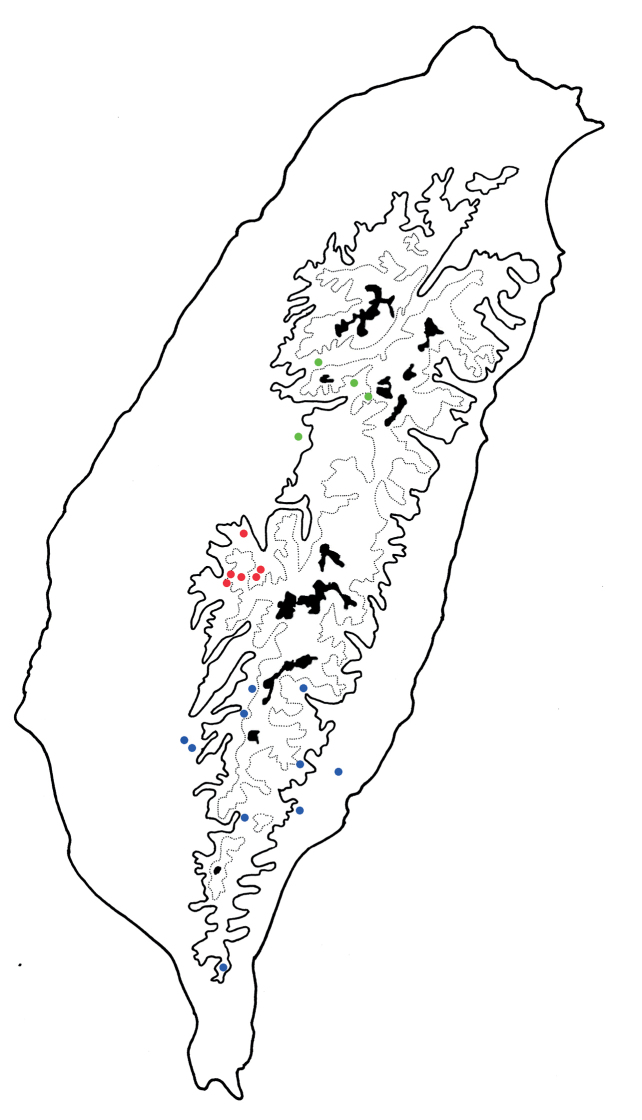
Distribution map of *Arthrotusgressitti* Kimoto, *A.hirashimai* Kimoto, and *A.yuae* sp. nov., solid line: 1000 m, broken line: 2000 m, black areas: 3000 m. Key: green dots *A.gressitti*, red dots *A.hirashimai*, blue dots *A.yuae* sp. nov.

Adults have been collected from several localities of central Taiwan, including Pasienshan (八仙山) in Taichung county; Peitungyanshan (北東眼山), Lushan (廬山), and Nanshanchi (南山溪) in north Nantou county (Fig. 5).

### ﻿ Arthrotus hirashimai

Taxon classificationAnimaliaColeopteraChrysomelidae

Kimoto, 1969

B1FAD6D8-F7B3-5AB1-8795-670717E28EC3

Figs 5 , 6A–C , 7

Arthrotus hirashimai Kimoto, 1969: 60.
Proegmena taiwana Takizawa, 1978: 125. syn. nov.
Arthrotus taiwana : Kimoto 1996: 40 (transferred from Proegmena); Kimoto and Takizawa, 1997: 390 (catalogue).

#### Types.

*Arthrotushirashimai*. ***Holotype*** ♂ (KUEC): “(Taiwan) / 5–10 km S of Fen- / chihu (奮起湖), Chiayi Hsien [p, w] // 11 [h] .iv.1965 / Y. Hirashima [p, w] // Japan-U. S. / Co-op. Sci. / Programme [p, y] // Arthrotus / hirashimai / Kimoto, n. sp. [h, w] // HOLOTYPE [p, r]”. ***Paratype***: 1♀ (KMNH), same data as holotype but with “PARATOPOTYPE [p, b]”.

*Proegmenataiwana*. The male holotype and one male paratype should be deposited at the Hokkaido University but were not found (Takemoto pers. comm., 23 Sept 2021). Two paratypes were deposited at the Takizawa’s private collection. 1♀ (HTC): “Chitou (溪頭) Chu- / shan (竹山) Taiwan / 6–7.VII.1975 / H. Takizawa [p, w] // Paratype [h, red letter] // Proegmena / taiwana n. sp. [h] / para-T [h, red letters] 197[p]7.2[h] / Det. H. Takizawa [p, w] (on the back of the same card)”; 1♂ (HTC): “(male aedeagus glued on the card) // Chitou (溪頭) Chu- / shan (竹山) Taiwan / 6–7.VII.1975 / H. Takizawa [p, w] // PARATYPE (?) / Proegmena / taiwana [h, w] // 2021.X.15 / H. Takizawa / det. [p, w] (on the back of the same card). However, both paratypes should be females based on the original description (Takizawa 1978).

#### Other material

**(*n* = 40).** Taiwan. Chiayi: 3♂, 1♀ (TARI), Alishan (阿里山), 29.V.2016, leg. Y.-T. Chung; 3♂, 2♀ (TARI), same but with “leg. B.-X. Guo; 1♀ (TARI), Fenchihu (奮起湖), 25.V.1981, leg. K. Sasagawa; 3♀ (HTC), same locality, 11–12.VII.1981, leg. H. Takizawa; 1♀ (TARI), Tutzuhu trail (杜仔湖步道), 1.VI.2014, leg. W.-C. Liao; Nantou: 1♂ (TARI), Hsitou (溪頭), 15.VI.2011, leg. C.-F. Lee; 2♂ (TARI), Tungfu (同富), 9.VI.2009, leg. C.-F. Lee; 1♂, 3♀ (HTC), Tongpu (= Tungpu, 東埔), 5–10.VII.1977, leg. H. Takizawa; 2♀ (HTC), same but with “6–8.VII.1981”; 2♂, 6♀ (HTC), same but with “16–18.VII.1995”; 3♂, 5♀ (TARI), same locality, 19–23.VII.1982, leg. L. Y. Chou & T. Lin; 1♀ (TARI), same locality, 20–24.VI.1983, leg. K. C. Chou & C. Y. Wong.

#### Diagnosis.

**Figure 6.**
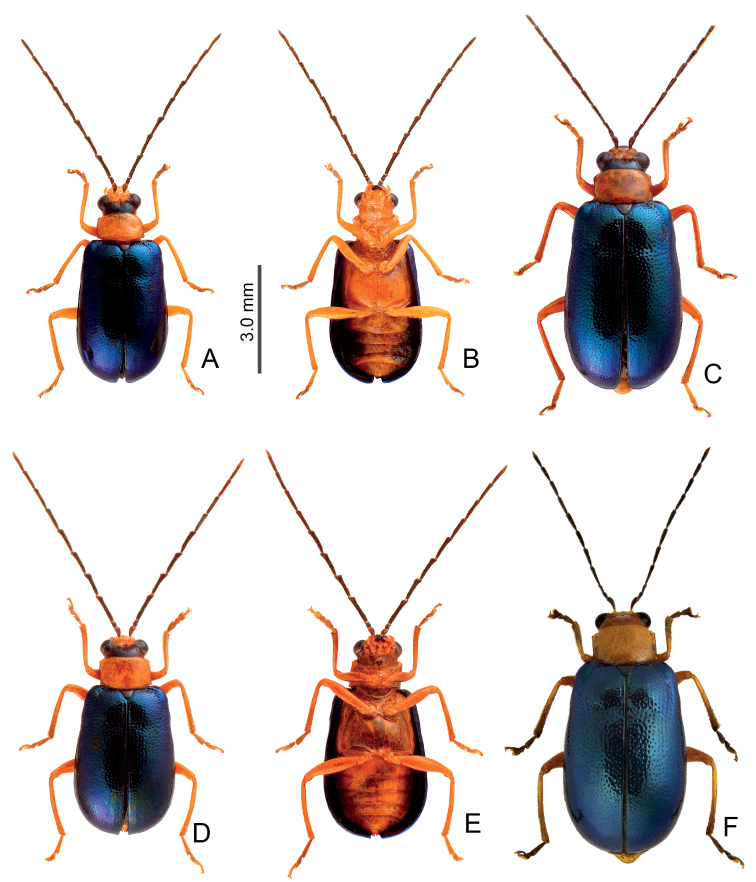
Habitus of *Arthrotushirashimai* Kimoto and *A.yuae* sp. nov. **A***A.hirashimai*, male, dorsal view **B***A.hirashimai*, male, ventral view **C***A.hirashimai*, female, dorsal view **D***A.yuae* sp. nov., male, dorsal view **E***A.yuae* sp. nov., male, ventral view **F***A.yuae* sp. nov., female, dorsal view.

**Figure 7.**
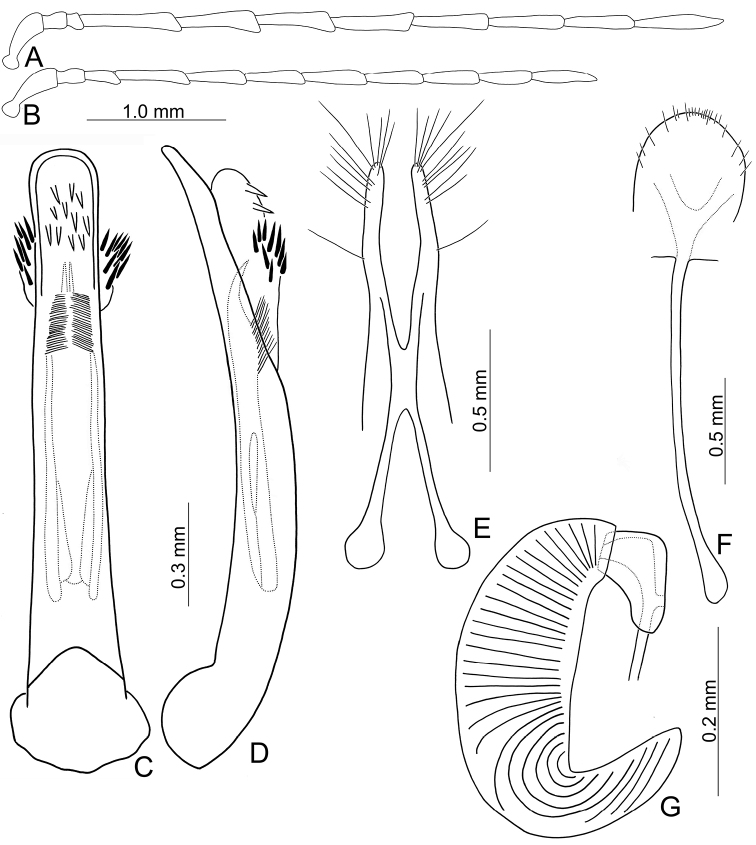
Diagnostic characters of *Arthrotushirashimai* Kimoto **A** antenna, male **B** antenna, female **C** aedeagus, dorsal view **D** aedeagus, lateral view **E** gonocoxae **F** abdominal ventrite VIII **G** spermatheca.

**Figure 8.**
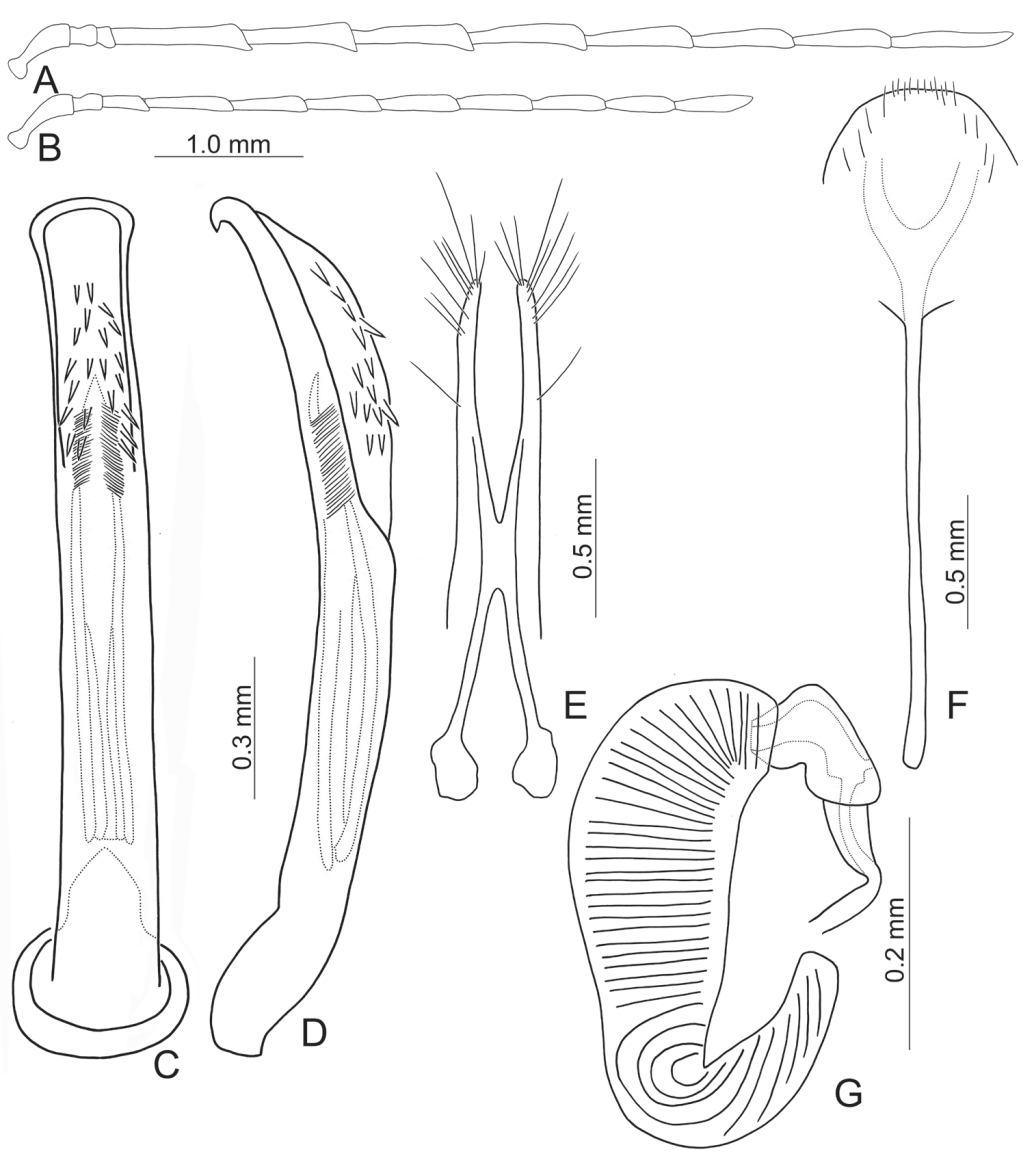
Diagnostic characters of *Arthrotusyuae* sp. nov. **A** antenna, male **B** antenna, female **C** aedeagus, dorsal view **D** aedeagus, lateral view **E** gonocoxae **F** abdominal ventrite VIII **G** spermatheca.

Adults of *Arthrotushirashimai* Kimoto (Fig. 6A–C) are similar to those of *A.abdominalis* (Chûjô) (Fig. 1A–C), *A.gressitti* Kimoto (Fig. 1D–F), and *A.yuae* sp. nov. (Fig. 6D–F) in possessing metallic blue elytra with transverse depression at basal 1/3 (various elytra without transverse depression in other congeners), and straight lateral margins of the pronotum (rounded lateral margins of the pronotum in other congeners). Adults of *A.hirashimai* (Fig. 6A–C) and *A.yuae* sp. nov. (Fig. 6D–F) are recognized by their yellowish-brown heads, thoraces, and legs (metallic blue head, thorax, and legs in *A.abdominalis* (Fig. 1A–C); black head, thorax, and legs in *A.gressitti* (Fig. 1D–F)); and less slender antennae, anternnomeres V–VIII < 5.5 × longer than wide (> 5.5 × longer than wide in *A.abdominalis*). Males of *A.hirashimai* are different from those of *A.yuae* sp. nov. by the tectum of the aedeagus being covered with short needle-shaped setae laterally and stout teeth apically (Fig. 7C) (covered with stout teeth in *A.yuae* sp. nov. (Fig. 8C)), and apex of aedeagus curved (Fig. 7D) (apex of aedeagus recurved in *A.yuae* sp. nov. (Fig. 8D)). In addition, both species are allopatric. Adults of *A.hirashimai* are found at mid-elevations (1000–2500 m) of central Taiwan while those of *A.yuae* sp. nov. are restricted to lowlands (below 1500 m) of southern Taiwan (Fig. 5).

#### Redescription.

Body color yellowish brown, elytra metallic blue, antennae black, vertex darkened in most of individuals (Fig. 6A–C). Pronotum with median transverse depression; dull, with reticulate microsculpture; with sparse, fine punctures confused with a few coarse punctures; lateral margins straight, basally narrowed; apical and basal margins slightly concave. Elytra with rounded lateral margins, widest at apical 1/3, disc shiny, without reticulate microsculpture, and with dense, coarse punctures, with distinct transverse depression at basal 1/3.

**Male.** Length 5.6–6.4 mm, width 2.6–2.7 mm. Antennae filiform (Fig. 7A), antennomere III modified, much shorter than II, IV–VII apically widened, length ratios of antennomeres I–XI 1.0: 0.4: 0.3: 1.6: 1.4: 1.3: 1.4: 1.3: 1.3: 1.2: 1.5, length to width ratios of antennomeres I–XI 2.9: 1.6: 0.9: 4.8: 3.6: 3.8: 4.4: 4.5: 5.2: 5.4: 6.1. Pronotum 1.5–1.6 × wider than long. Elytra 1.6 × longer than wide. Aedeagus (Fig. 7C, D) extremely slender, ~ 10.2 × longer than wide, parallel-sided, slightly narrowed at apical 1/4, basally widened, apex widely rounded; tectum membranous, sides covered with densely, well-sclerotized, stout setae; weakly curved in lateral view, apex recruved; primary endophallic sclerite elongate, ~ 0.8 × as long as aedeagus, apex pointed, with a cluster of dense setae near apex, deeply bifurcate from middle to base; a pair of dorsal slclerite longitudinal and apically connected with primary sclerite.

**Female.** Length 6.1–7.2 mm, width 2.8–3.4 mm. Antennae (Fig. 7B) much shorter than in males, antennomere III slightly longer than II, length ratios of antennomeres I–XI 1.0: 0.4: 0.5: 1.1: 1.0: 0.9: 1.0: 0.9: 0.9: 0.8: 1.0, length to width ratios of antennomeres I–XI 3.2: 1.9: 2.4: 5.1: 4.4: 4.2: 4.6: 4.3: 4.2: 4.1: 5.3. Pronotum 1.5–1.6 × wider than long. Elytra 1.6–1.7 × longer than wide. Ventrite VIII (Fig. 7F) membranous, apical widely rounded, with scattered long setae at sides and along apical margin, and dense short setae at apical margin; spiculum extremely slender. Receptacle of spermatheca (Fig. 7G) slightly swollen, undivided from pump; pump narrow and moderately curved, apex truncate; sclerotized proximal spermathecal duct wide and short, shallowly projecting into receptaculum. Gonocoxae (Fig. 7E) narrowly connected at middle, ~ 6.2 × longer than wide, curved inwards at apical 1/3, with one long seta at apical 1/3, nine additional setae at apical areas.

#### Food plants.

Unknown.

#### Distribution.

Adults are restricted to several localities at mid-elevations of central Taiwan, including Hsitou (溪頭), Tungfu (同富), and Tungpu (東埔) in south Nantou county; Alishan (阿里山), Fenchihu (奮起湖), and Tutzuhu trail (杜仔湖步道) in Chiayi county (Fig. 5).

### ﻿ Arthrotus yuae sp. nov.

Taxon classificationAnimaliaColeopteraChrysomelidae

5E5C1CF5-CC0A-5ABE-B2A0-5330ABDD1728

http://zoobank.org/0F41557E-694E-4820-AEF8-2D77436D6D27

Figs 5 , 6D–F , 8

#### Type series

**(*n* = 160). *Holotype*** ♂ (TARI): Taiwan. Pingtung: Tahanshan (大漢山), 25.V.2013, leg. Y.-T. Chung. Paratypes. Taiwan. Kaohsiung: 1♀ (TARI), Chungchihkuan (中之關), 13.X.2012, leg. L-P. Hsu; 1♂ (KMNH), 溪南山 (= Shinanshan), 20.IV.1991, leg. W. Chen; 1♀ (TARI), Tona trail (多納林道), 13.X.2012, leg. W.-C. Liao; 2♂ (HTC), Wukongshan (五公山), 2.V.1996, leg. H. Takizawa; 1♀ (TARI), Wutai (霧台), 19.V.2009, leg. U. Ong; Pingtung: 1♀ (TARI), T(D)ahanshan (大漢山), 24.VI.2007, leg. C.-F. Lee; 2♂, 7♀ (TARI), same but with “18.VII.2007”; 4♂, 1♀ (TARI), same but with “25.V.2008”; 1♂ (TARI), same but with “4.IV.2009”; 16♂, 13♀ (TARI), same but with “6.VI.2012”; 2♂, 18♀ (TARI), same but with “19.VII.2012”; 2♀ (TARI), same locality, 18.VII.2007, leg. M.-H. Tsou; 2♀ (TARI), same but with “20.VII.2007”; 3♂, 6♀ (TARI), same but with “4.VII.2008”; 1♀ (TARI), same locality, 18.VII.2007, leg. S.-F. Yu; 2♀ (TARI), same but with “20.VII.2007”; 3♀ (TARI), same but with “4.VII.2008”; 1♀ (TARI), same locality, 18.V.2009, leg. M.-L. Jeng; 2♀ (TARI), same locality, 28.VI.2009, leg. Y(I).-T. Chung; 2♀ (TARI), same but with “14.VIII.2011”; 1♀ (TARI), same but with “6.VII.2012”; 1♀ (TARI), same but with “5.VIII.2012”; 1♂ (TARI), same but with “16.IV.2013”; 2♂ (TARI), same but with “24.IV.2013”; 4♂, 1♀ (TARI), same but with “5.V.2013”; 1♂ (TARI), same but with “10.V.2013”; 2♂, 5♀ (TARI), same but with 25.V.2013”; 1♂, 1♀ (TARI), same but with “2.VI.2013”; 3♀ (TARI), same but with “2.VII.2013”; 3♀ (TARI), same but with “9–10.VII.2013”; 1♀ (TARI), same but with “30.VII.2013”; 5♂ (TARI), same but with “30.V.2014”; 1♂ (TARI), same but with “6.VI.2014”; 1♀ (TARI), same but with “17.VIII.2014”; 1♀ (TARI), same but with “14.IX.2014”; 1♀ (TARI), same but with “4.X.2014”; 1♀ (TARI), same but with “6.VI.2015”; 1♀ (TARI), same but with “29.VI.2018”; 5♀ (TARI), same locality, 14.VIII.2011, leg. Y.-T. Wang; 1♀ (TARI), same locality, 4–5.VI.2013, leg. K. Takahashi; 1♂, 3♀ (TARI), same locality, 29.VI.2013, leg. B.-X. Guo; 3♀ (TARI), same but with “3.VII.2013”; 2♀ (TARI), same locality, 13.VI.2015, leg. W.-C. Liao; 1♀ (TARI), same but with “28.VI.2015”; Taitung: 1♂ (TARI), Lichia (trail) (利嘉(林道)), 19.V.2009, leg. U. Ong; 1♀ (TARI), same locality, 15.VII.2014, leg. B.-X. Guo; 2♀ (TARI), same locality, 17.VII.2014, leg. W.-T. Wang; 2♀ (TARI), same locality, 25.VII.2015, leg. Y.-T. Chung, P.-H. Kuo (= B.-X. Guo) & S.-P. Wu; 2♀ (TARI), same locality, 1.VII.2016, leg. C.-C. Chen; 1♀ (TARI), same but with “leg. B.-X. Guo”; 1♀ (TARI), Liyuan (栗園), 23.VI.2010, leg. M.-H. Tsou; 1♂ (TARI), same locality, 19.VI.2013, leg. C.-F. Lee; 2♀ (TARI), Tulanshan (都蘭山), leg. S.-P. Wu; 1♀ (TARI), Yanping trail (延平林道), 5.VII.2016, leg. S.-P. Wu.

#### Diagnosis.

Adults of *Arthrotusyuae* sp. nov. (Fig. 6D–F) are similar to those of *A.abdominalis* (Chûjô) (Fig. 1A–C), *A.gressitti* Kimoto (Fig. 1D–F), and *A.hirashimai* Kimoto (Fig. 6A–C) in possessing metallic blue elytra with a transverse depression at basal 1/3 (various elytra without transverse depression in other congeners), and straight lateral margins of the pronotum (rounded lateral margins of the pronotum in other congeners). Adults of *A.yuae* sp. nov. (Fig. 6D–F) and *A.hirashimai* (Fig. 6A–C) are recognized by their yellowish brown heads, thoraxes, and legs (metallic blue head, thorax, and legs in *A.abdominalis* (Fig. 1A–C); black head, thorax, and legs in *A.gressitti* (Fig. 1D–F)); and less slender antennae, anternnomeres V–VIII < 5.5 × longer than wide (> 5.5 × longer than wide in *A.abdominalis*). Males of *A.yuae* sp. nov. differ from those of *A.hirashimai* by the tectum of the aedeagus being covered with stout teeth (Fig. 8C) (covered with short needle-shape laterally and stout teeth apically in *A.hirashimai* (Fig. 7C)), and apex of aedeagus recurved (Fig. 8D) (apex of aedeagus curved in *A.hirashimai* (Fig. 7D)). In addition, both species are allopatric. Adults of *A.yuae* sp. nov. are restricted to lowlands (below 1500 m) of south Taiwan while those of *A.hirashimai* are found at mid-elevations (1000–2500 m) of central Taiwan (Fig. 5).

#### Description.

Color (Fig. 6D–F) yellowish brown, elytra metallic blue, antennae black, vertex darker in a few individuals. Pronotum with median transverse depression reduced, dull, with reticulate microsculpture; with sparse fine punctures confused with a few coarse punctures; lateral margins straight, basally narrowed; apical and basal margins slightly concave. Elytra with rounded lateral margins, widest at apical 1/3; disc shiny, without reticulate microsculpture, and with dense, coarse punctures, with distinct transverse depression at basal 1/3.

**Male.** Length 5.7–7.0 mm, width 2.8–3.4 mm. Antennae filiform (Fig. 8A), antennomere III modified, much shorter than II, IV–VII apically widened, length ratios of antennomeres I–XI 1.0: 0.4: 0.2: 1.8: 1.6: 1.6: 1.6: 1.5: 1.4: 1.4: 1.8, length to width ratios of antennomeres I–XI 3.1: 1.3: 0.7: 4.8: 3.7: 4.3: 4.2: 5.1: 5.8: 5.8: 8.4. Pronotum 1.5–1.6 × wider than long. Elytra 1.5–1.6 × longer than wide. Aedeagus (Fig. 8C, D) extremely slender, ~ 8.8 × longer than wide, parallel-sided, slightly narrowed at apical 1/4, basally widened, apex widely rounded; tectum membranous, covered with dense, well-sclerotized, stout setae, and clustered elongate setae laterally; weakly curved in lateral view, apex narrowly rounded; primary endophallic sclerite elongate, ~ 0.5 × as long as aedeagus, apex pointed, with a cluster of dense setae near apex, deeply bifurcate from middle to base; a pair of dorsal sclerites longitudinally and apically connected with primary sclerite.

**Female.** Length 6.0–7.7 mm, width 3.3–3.9 mm. Antennae much shorter than in males, antennomere III a little longer than II (Fig. 4C), length ratios of antennomeres I–XI 1.0: 0.4: 0.6: 1.2: 1.0: 1.0: 1.1: 1.0: 1.0: 0.8: 1.1, length to width ratios of antennomeres I–XI 3.5: 1.7: 3.4: 6.3: 5.1: 4.8: 4.8: 4.6: 4.6: 4.1: 4.9. Pronotum 1.5 × wider than long. Elytra 1.5 × longer than wide. Ventrite VIII (Fig. 8F) membranous, apical margin widely rounded, with scattered long setae at sides and along apical margin, and dense, short setae at apical margin; spiculum extremely slender. Receptacle of spermatheca (Fig. 8G) slightly swollen, undivided from pump; pump narrow and moderately curved, apex broadly rounded; sclerotized proximal spermathecal duct wide and short, shallowly projecting into receptaculum. Gonocoxae (Fig. 8E) narrowly connected at middle, ~ 5.4 × longer than wide, curved inwards at apical 1/3, with one long seta at apical 1/3, ten additional setae at apical areas.

#### Food plants.

Leaves of *Achyranthesbidentata* Blume (Amaranthaceae), and Prunusphaeostictavar.phaeosticta (Hance) Maxim. (Rosaceae).

#### Distribution.

Adults are restricted to several localities at lowlands of southern Taiwan including Chungchihkuan (中之關), Shinanshan (溪南山), Tona trail (多納林道), and Wutai (霧台) in Kaohsiung county; Tahanshan (大漢山) in Pingtung county; Lichia trail (利嘉林道), Liyuan (栗園), Tulanshan (都蘭山), and Yanping trail (延平林道) in Taitung county (Fig. 5).

#### Etymology.

Dedicated to Mrs Su-Fang Yu (余素芳) who was the first member of TCRT to collect specimens of this new species.

### ﻿ Arthrotus fulvus

Taxon classificationAnimaliaColeopteraChrysomelidae

Chûjô, 1938

90A5C025-ED90-5D00-ABAF-0EAFC3AC1B01

**Figure 9.**
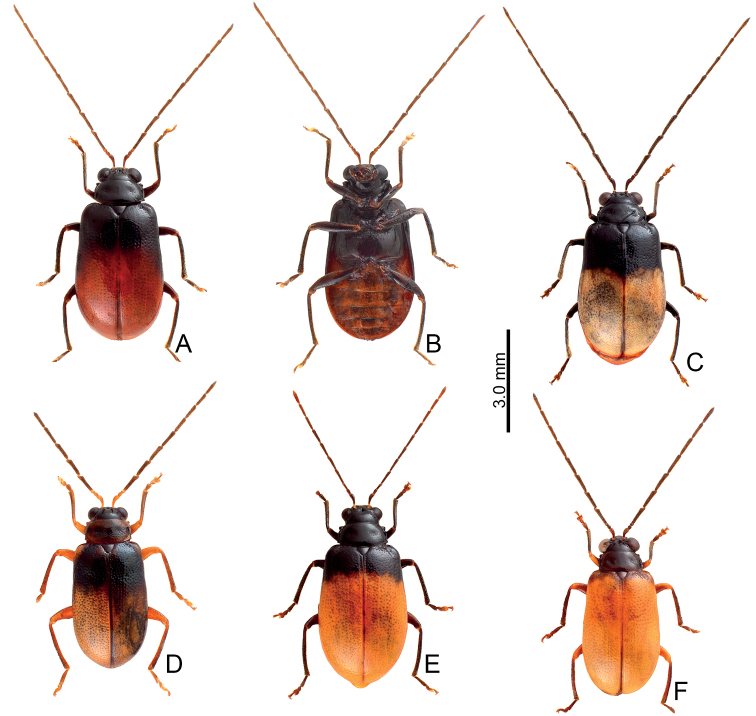
Habitus of *Arthrotusfulvus* Chûjô **A** collected from Kuanwu (觀霧), male, dorsal view **B** collected from Kuanwu (觀霧), male, ventral view **C** collected from Tzuchung (自忠), male, dorsal view **D** collected from Tungfu (同富), male, dorsal view **E** collected from Motien (摩天), female, dorsal view **F** collected from Motien (摩天), male, dorsal view.

**Figure 10.**
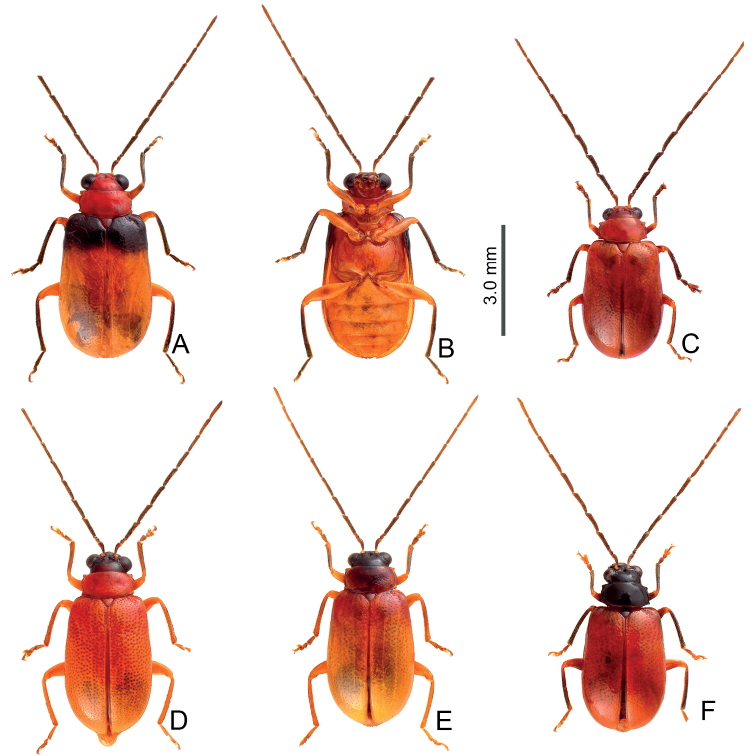
Habitus of *Arthrotusfulvus* Chûjô **A** collected from Liyuan (栗園), male, dorsal view **B** collected from Liyuan (栗園), male, ventral view **C** collected from Tengchih (藤枝), male, dorsal view **D** collected from Tahanshan (大漢山), male, dorsal view **E** collected from Tahanshan (大漢山), male, dorsal view **F** collected from Erhchituan (二集團), male, dorsal view.

**Figure 11.**
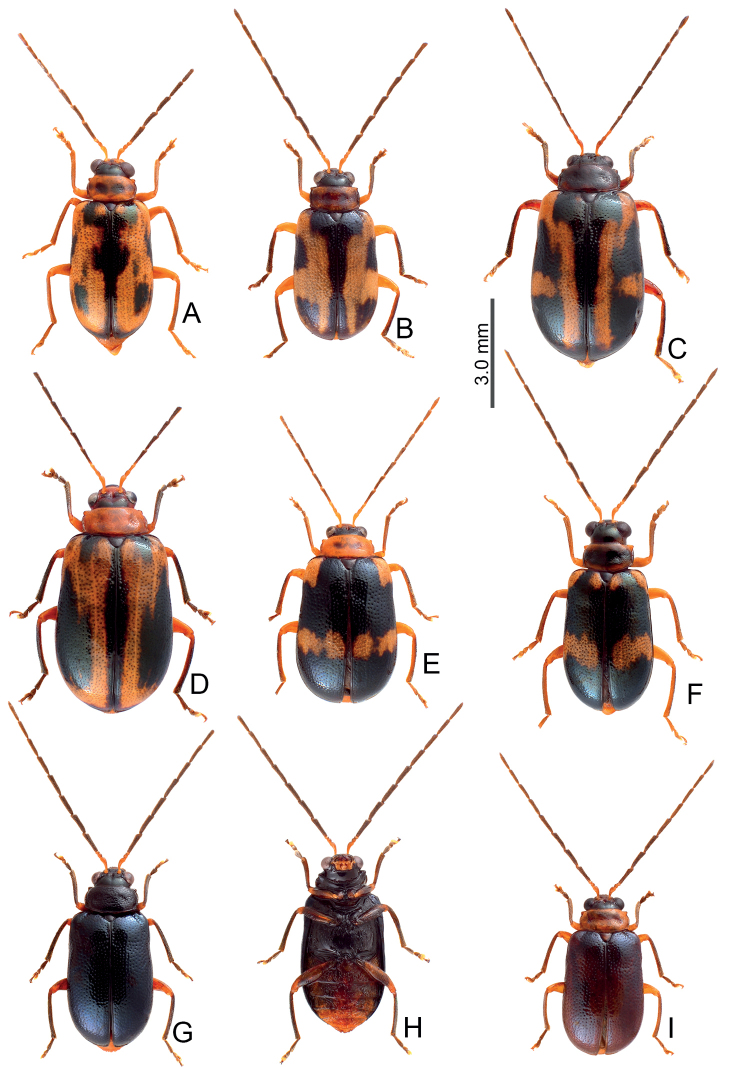
Habitus of *Arthrotusfulvus* Chûjô **A** collected from Motien (摩天), male, dorsal view **B** collected from Chenghsipao (鎮西堡), male, dorsal view **C** collected from Pilu (碧綠), female, dorsal view **D** collected from Taipingshan (太平山), female, dorsal view **E** collected from Wufeng (五峰), female, dorsal view **F** collected from Chungchihkuan (中之關), male, dorsal view **G** collected from Tsuifeng (翠峰), male, dorsal view **H** collected from Tsuifeng (翠峰), male, venral view **I** collected from Meifeng (梅峰), male, dorsal view.

**Figure 12.**
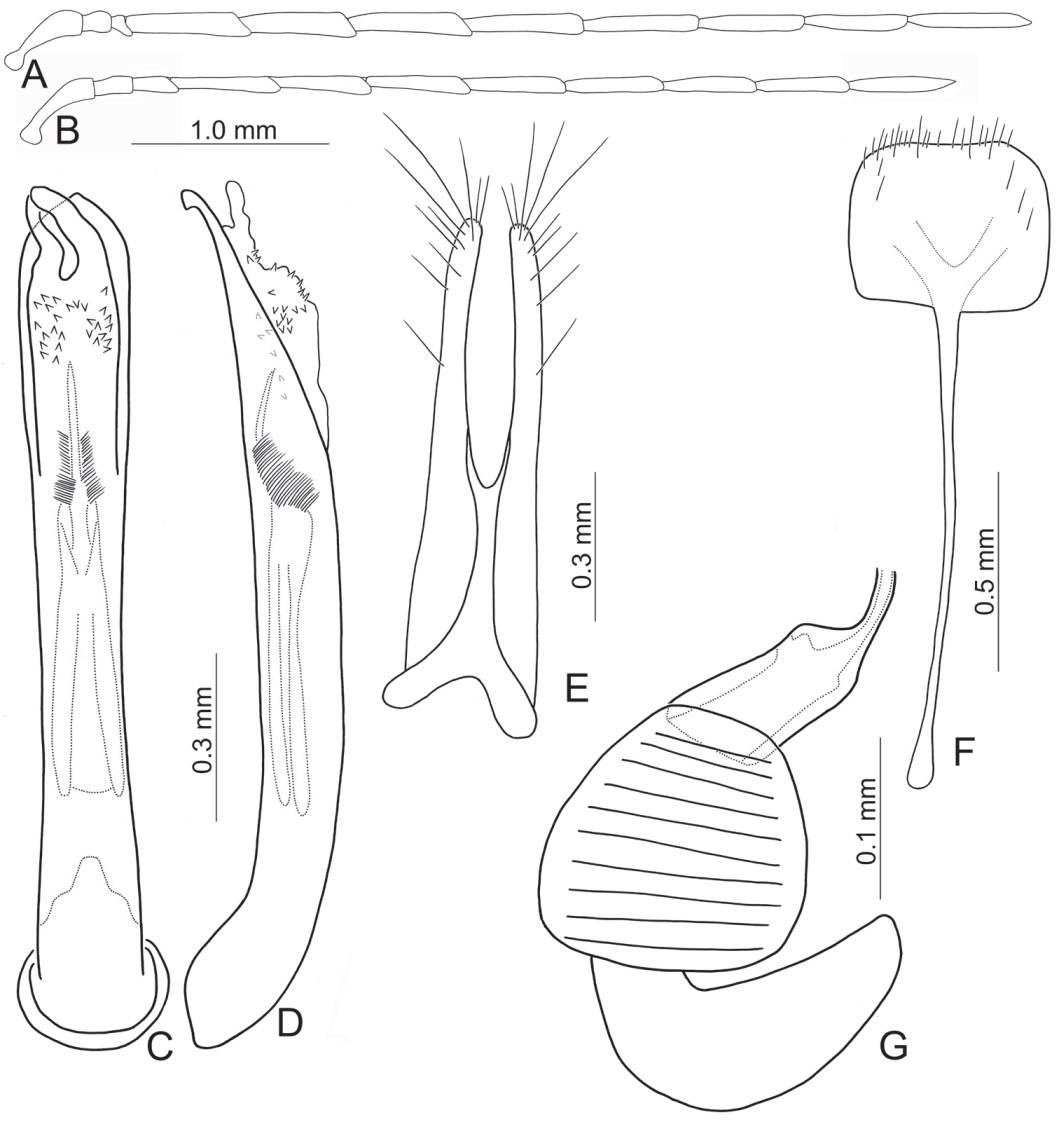
Diagnostic characters of *Arthrotusfulvus* Chûjô **A** antenna, male **B** antenna, female **C** aedeagus, dorsal view **D** aedeagus, lateral view **E** gonocoxae **F** abdominal ventrite VIII **G** spermatheca.

**Figure 13.**
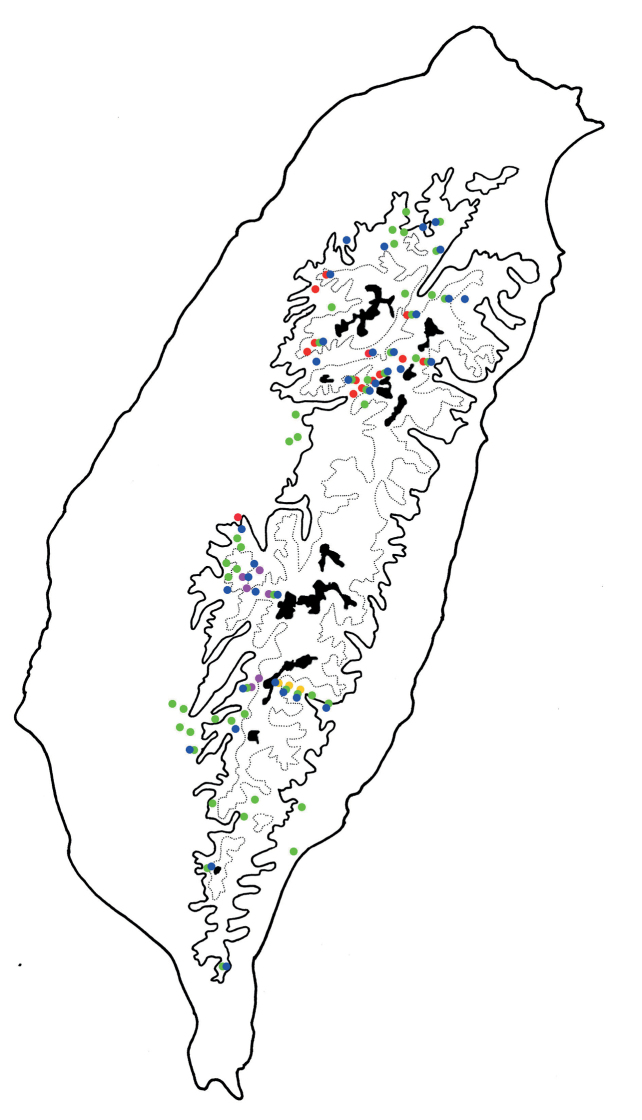
Distribution map of *Arthrotusfulvus* Chûjô; solid line: 1000 m, broken line: 2000 m, black areas: 3000 m. Key: red dots Form A, pink dots Form B, yellow dots Form C, green dots yellowish or reddish brown elytra, blue dots maculate or metallic blue elytra.

Figs 9 , 10 , 11 , 12 , 13

Arthrotus fulvus Chûjô, 1938: 139; Kimoto 1987: 190 (additional records); Kimoto 1989: 259 (additional records); Kimoto 1991: 16 (additional records).
Arthrotus testaceus : Kimoto 1969: 60 (part).
Dercetina nakanei Kimoto, 1969: 65. syn. nov.
Arthrotus nakanei : Lee and Bezdĕk 2013: 28 (transferred from Dercetina).

#### Types.

*Arthrotusfulvus*. Lectotype ♀ (TARI) here designated for clarifying its species identity which was confused with *A.testaceus*, labeled: “Chipon (= Chihpen, 知本) [h] / FORMOSA [p] / 25.III.1935 [h] / COL. M. CHUJO [p, w]”; CO / Type [p, w, circle label with yellow letters and yellow border] // Arthrotus / fulvus / Chûjô [h] / DET. M. CHUJO [p, w] // 1369 [p, w]”. Paralectotypes. 1♀ (TARI): “Formosa / Koshun (= Henchun, 恆春), 1918 / IV 25–V 25. / J. Sonan, [p, w] // CO / Type [p, w, circle label with yellow letters and yellow border] // Arthrotus / fulvus / Chûjô [h] / DET. M. CHUJO [p, w] // 2589 [p, w]”; 1♂ (TARI, lacking head): “Formosa / Shinchiku (= Hsinchu, 新竹), -18 / VII 1–30, / J. Sonan, // CO / Type [p, w, circle label with yellow letters and yellow border] // Arthrotus / fulvus / Chûjô [h] / DET. M. CHUJO [p, w] // 1495 [p, w]”

*Dercetinanakanei*. Types were studied by Lee and Bezdĕk (2013).

#### Other material.

A total of 603 specimens was examined (Suppl. material 2).

#### Diagnosis.

Adults of *Arthrotusfulvus* Chûjô are similar to those of *A.tricolor* (Chûjô) in possessing rounded lateral margins of their pronota (straight lateral margins in *A.abdominalis* (Chûjô), *A.gressitti* Kimoto, *A.hirashimai* Kimoto, and *A.yuae* sp. nov.), the less transverse pronotum and elytra, 1.7–2.0 × wider and long in pronotum and 1.5–1.6 × longer than wide in elytra (more transverse pronotum and elytra, 2.1–2.2 × wider and long and 1.4 × longer than wide in elytra of *A.testaceus* Gressitt & Kimoto and *A.yangi* sp. nov.), the more transverse antennomere III in males, 0.7–0.8 × longer than wide (Fig. 12A) (the less transverse antennomere III in males, 1.1 × longer than wide in *A.saigusai* Kimoto (Fig. 16A)). Adults of *A.fulvus* (Figs 9–11) are different from those of *A.tricolor* in the absence of the characterstic color patterns of *A.tricolor* (Fig. 19), tectum of aedeagus with one pair of apical tube-like processes and disc covered with scattered short setae (Fig. 12C, D) (without pairs of apical tube-like processes and disc covered with clustered stout setae in *A.tricolor* (Fig. 20C, D).

#### Redescription.

Some color patterns characteristic and restricted to particular areas. **Form A** (described as *A.nakanei*) (Fig. 9A, B): color black, abdomen and apical 2/3 of elytra reddish brown. **Form B** (Fig. 9C): similar to Form A, but reddish brown areas replaced with white ones; some with reddish brown head and pronotum (Fig. 9D); some specimens with black areas replaced with reddish brown; some specimens with yellowish brown legs, apical and basal areas of pronotum, one pair of yellow spots on humeral calli, sometimes surrounding scutellum, which is also yellow. **Form C** (Fig. 9E): similar to Form B, but white areas replaced with orange spots; some adults similar to Form C but with yellowish brown femora and almost entirely yellow elytra except basal area (Fig. 9F); some similar to Form C, but head, pronotum, and scutellum reddish brown, legs entirely black or yellow except tibiae and tarsi (Fig. 10A, B). **Yellowish or reddish brown elytra**: some adults with entirely yellow or reddish brown bodies except antenna (Fig. 10C); sometimes heads darker (Fig. 10D); some with dark head, pronotum dark brown centrally, elytra reddish brown basally (Fig. 10E); some adults with black heads, pronota, and scutella, and tibiae and tarsi darker (Fig. 10F). **Maculate or metallic blue elytra**: Pale individuals have dark brown vertex, blackish brown spots on sides of pronotum, some extending onto most of the pronotum, black stripes along lateral and basal margins of elytra, with additional dark spots near base, and near lateral margin at basal and basal 1/3, near suture at middle (Fig. 11A); sometimes dark spots near suture at middle reduced and black stripes along suture widened (Fig. 11B); sometimes anterior and posterior spots at sides widened (Fig. 11C), some individuals with both (Fig. 11D). Dark individuals with metallic blue elytra with one or two pairs yellow spots along basal margin and transverse yellow stripes at middle (Fig. 11E, F). Some entirely metallic blue, but antennae (except three basal antennomeres) and legs blackish brown and abdomen yellow (Fig. 11G, H); some with yellowish brown bodies, but elytra entirely metallic blue; antennae except three basal antennomeres, tibiae, and tarsi dark or blackish brown (Fig. 11I). Pronotum with median transverse depression; shiny, without reticulate microsculpture; with spare coarse punctures confused; lateral margins rounded, widest at middle; apical and basal margins slightly concave. Elytra parallel-sided; disc shiny, without reticulate microsculpture, and with dense, coarse punctures.

**Male.** Length 5.1–5.4 mm, width 2.5–2.8 mm. Antennae filiform (Fig. 12A), antennomere III shorter than II, IV–VII relatively wider, length ratios of antennomeres I–XI 1.0: 0.3: 0.2: 1.3: 1.3: 1.3: 1.4: 1.3: 1.2: 1.1: 1.4, length to width ratios of antennomeres I–XI 3.5: 1.4: 0.7: 4.5: 4.3: 4.8: 5.6: 7.2: 6.7: 6.4: 8.4. Pronotum 1.9–2.0 × wider than long. Elytra 1.5–1.6 × longer than wide. Aedeagus (Fig. 12C, D) extremely slender, ~ 10.8 × longer than wide, slightly narrowed medially; tectum membranous, covered with weakly sclerotized, tiny setae; weakly curved in lateral view, apex recurved; primary endophallic sclerite elongate, ~ 0.5 × as long as aedeagus, with a cluster of dense setae near apex; deeply bifurcate from middle to base.

**Female.** Length 5.4–6.3 mm, width 2.6–3.3 mm. Antennae (Fig. 12B) much more slender than in males, antennomere III slightly longer than II, length ratios of antennomeres I–XI 1.0: 0.4: 0.4: 1.2: 1.1: 1.1: 1.1: 1.1: 1.0: 1.0: 1.2, length to width ratios of antennomeres I–XI 4.2: 2.9: 2.4: 6.2: 5.6: 5.6: 6.2: 6.2: 6.3: 6.3: 6.8. Pronotum 1.7–1.9 × wider than long. Elytra 1.5–1.6 × longer than wide. Ventrite VIII (Fig. 12F) weakly sclerotized, apical margin truncate, with sparse, short setae along apical margin, and sparse, long setae in inner transverse row; spiculum extremely slender. Receptacle of spermatheca (Fig. 12G) strongly swollen, divided from pump; pump narrow and moderately curved, apex narrowly rounded; sclerotized proximal spermathecal duct wide and short, shallowly projecting into receptaculum. Gonocoxae (Fig. 12E) connected from base to middle, ~ 5.0 × longer than wide, curved inwards apically, with one short seta at apical 1/3, nine additional setae at apical areas.

#### Remarks.

One specimen collected from Penpuchi (本部溪) was misidentified as *A.testaceus* by Kimoto (1969).

#### Host plants.

Leaves of AcerinsulareHayatavar.caudatifolium (Hayata) S.Y. Lu & Y.P. Yang (Sapindaceae), *Alnusformosana* (Burkill) Makino (Betulaceae), *Stachyurushimalaicus* Hook. f. & Thomson (Stachyuraceae), and *Persicariachinensis* (L.) H. Gross (Polygonaceae).

#### Distribution.

*Arthrotusfulvus* is widespread from lowlands to mid-elevations of Taiwan. Adult color forms A, B, and C are allopatric. Members of form A were collected from central Taiwan, including Hsinchu, Ilan, Hualien, north Nantou, and Taichung counties; color form B from southwest Taiwan, including south Nantou, Chiayi, and Kaoshiung counties; color form C from southeast Taiwan only, including Taitung county. Most adults with yellowish or reddish brown elytra were collected from lowlands, while most adults with maculate or metallic blue elytra were form mid-elevations (Fig. 13).

### ﻿ Arthrotus saigusai

Taxon classificationAnimaliaColeopteraChrysomelidae

Kimoto, 1969

22C7D88C-3FB3-517A-972B-566D337E467D

**Figure 14.**
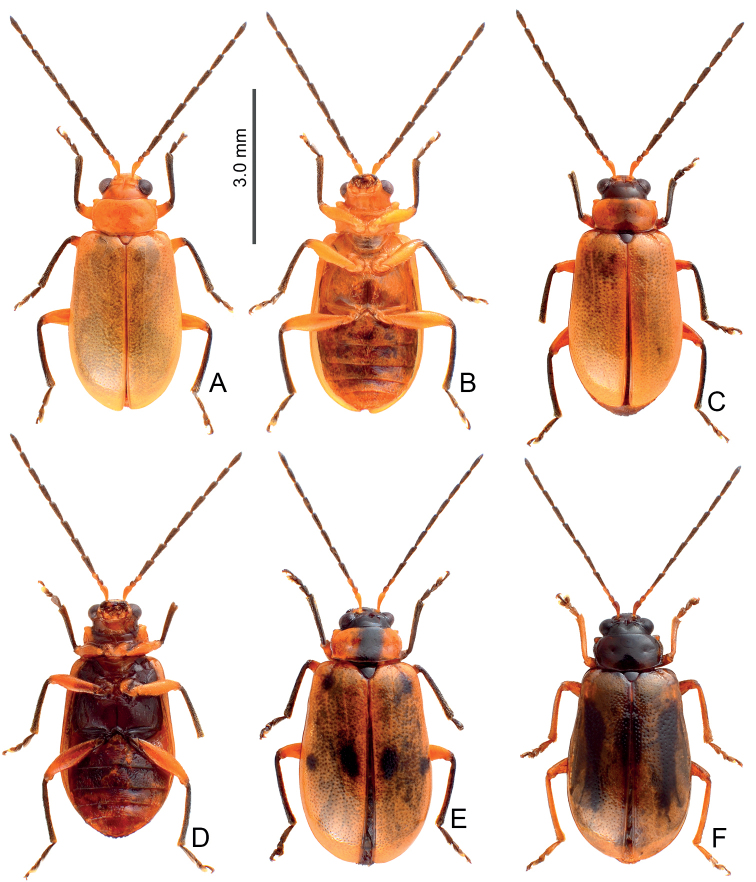
Habitus of *Arthrotussaigusai* Kimoto **A** collected from Kuanshan Wind Gap (關山啞口), male, dorsal view **B** collected from Kuanshan Wind Gap (關山啞口), male, ventral view **C** collected from Kuanshan Wind Gap (關山啞口), male, dorsal view **D** collected from Kuanshan Wind Gap (關山啞口), male, ventral view **E** collected from Tienchih (天池), female, dorsal view **F** collected from Huakang (華岡), female, dorsal view.

**Figure 15.**
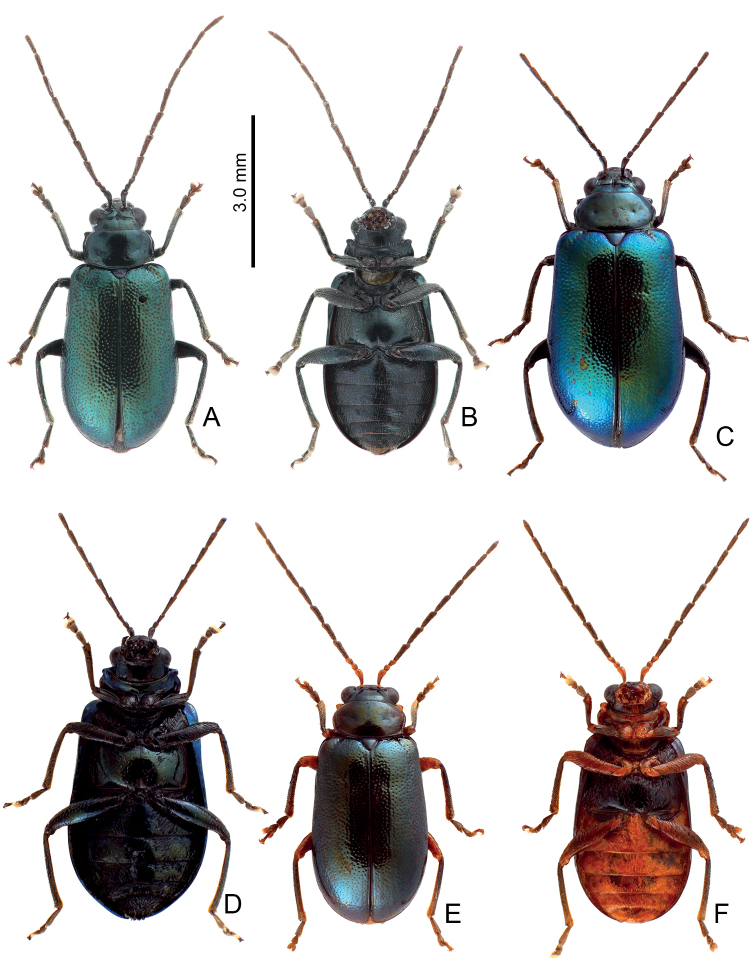
Habitus of *Arthrotussaigusai* Kimoto **A** collected from Hsiaofengko (小風口), male, dorsal view **B** collected from Hsiaofengko (小風口), male, ventral view **C** collected from Huakang (華岡), female, dorsal view **D** collected from Huakang (華岡), female, ventral view **E** collected from Hsiangyangshan (向陽山), male, dorsal view **F** collected from Hsiangyangshan (向陽山), male, ventral view.

**Figure 16.**
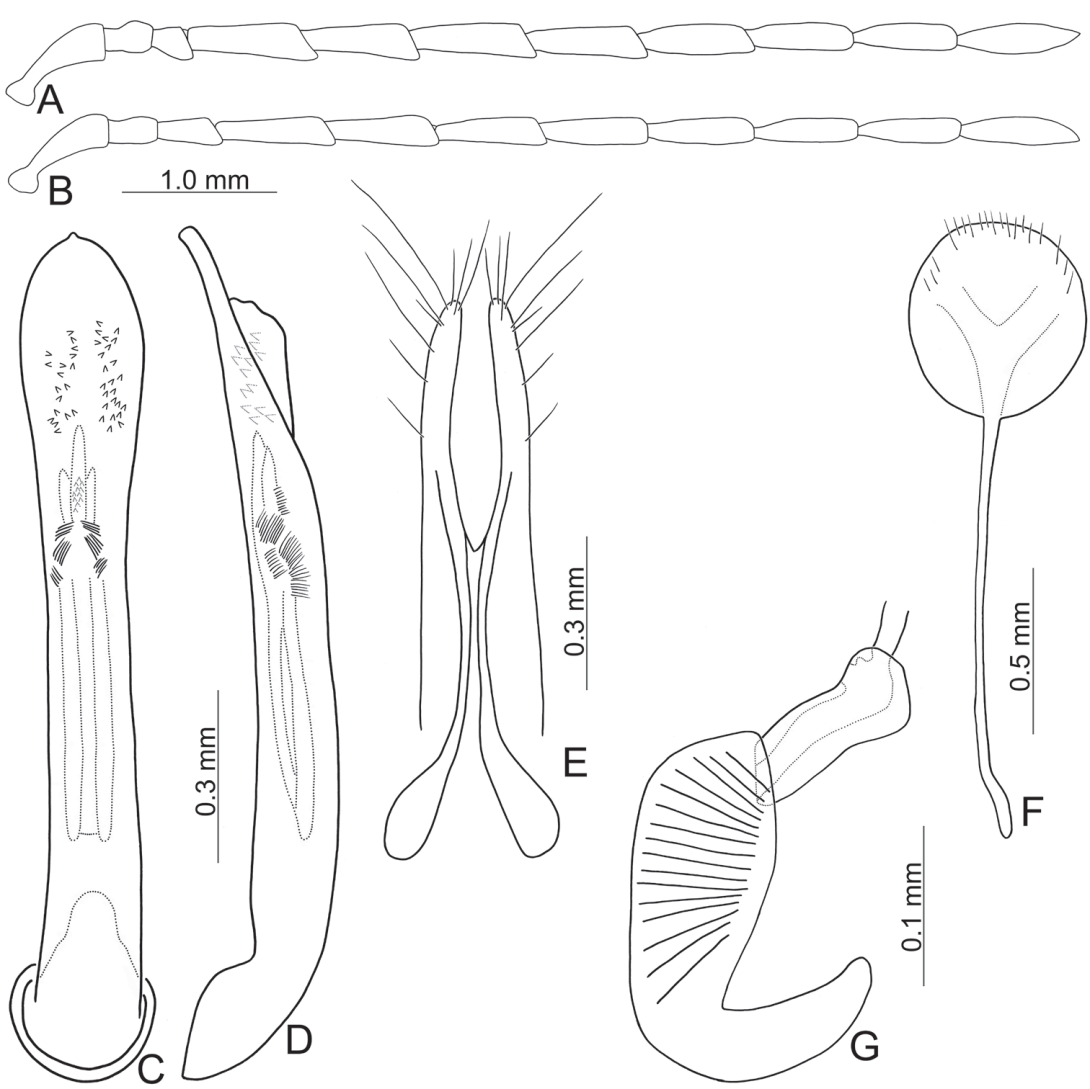
Diagnostic characters of *Arthrotussaigusai* Kimoto **A** antenna, male **B** antenna, female **C** aedeagus, dorsal view **D** aedeagus, lateral view **E** gonocoxae **F** abdominal ventrite VIII **G** spermatheca.

**Figure 17.**
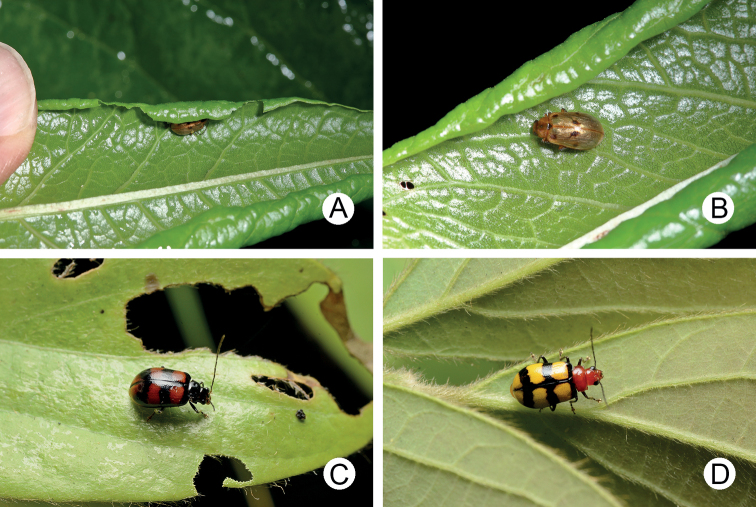
File photographs of *Arthrotus* species **A** adult of *Arthrotussaigusai* conceal under curled leaves of *Reynoutriajaponica***B** some adult come out from the curled leaves **C** blackened female of *A.tricolor* at Tahsuehshan (大雪山) **D** typical female of *A.tricolor* at the same locality.

**Figure 18.**
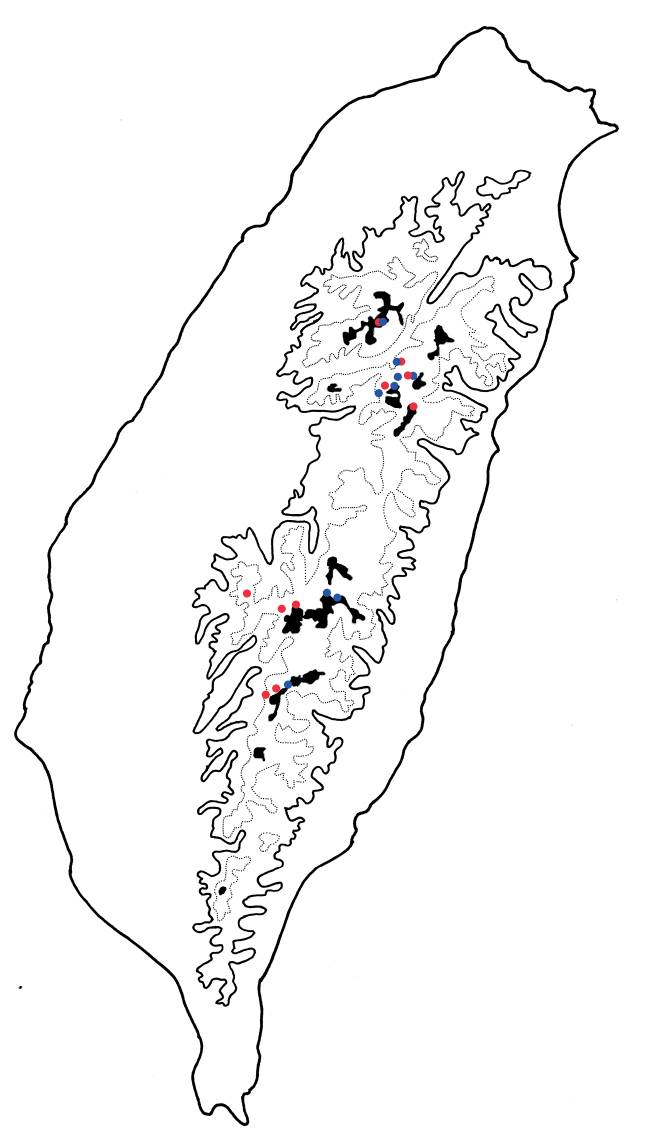
Distribution map of *Arthrotussaigusai* Kimoto; solid line: 1000 m, broken line: 2000 m, black areas: 3000 m. Key: red dots yellowish brown form, blue dots metallic blue form.

Figs 14 , 15 , 16 , 17A, B , 18

Arthrotus saigusai Kimoto, 1969: 62; Kimoto 1987: 190 (additional records).
Arthrotus fulvus : Kimoto 1989: 259 (part).

#### Types.

***Holotype*** ♂ (KUEC): “[Formosa] / Tung-pu (東埔) / Tzu-chung (自忠) / 10.IV.1965 . T. Saigusa [h, w] // HOLOTYPE [p, r] // Arthrotus / saigusai / Kimoto, n. sp. [h, w]”. ***Paratype***: 1♂ (KMNH): “(TAIWAN) / Tonp;ogoe (= Tungpu, 東埔) / 2500 m / Kagi-ken / 2. IV. 1967 / T. Shirozu [p, w] // Arthrotus / saigusai / Kimoto, n. sp. [h, w] // PARATYPE [p, b]”.

#### Other material.

**Yellowish brown form (*n* = 114)**: Taiwan. Chiayi: 1♀ (HTC), 阿里山 (Alishan), 23–24.IV.1928, leg. Matsumura; 1♀ (KMNH), Yushan (玉山), 19.V.1981, leg. N. Ito; Hualien: 3♀ (HTC), Taruling (= Tayuling, 大禹嶺), 9.VII.1983, leg. H. Takizawa; 3♂ (NMNS), same locality, 25.VI.2008, leg. H.-H. Lin; Kaohsiung: 26♂, 11♀ (TARI), Kuanshan Wind Gap (關山啞口), 24.IX.2015, leg. C.-F. Lee; 4♂, 2♀ (TARI), same locality, 25.X.2015, leg. B.-X. Guo; 1♂, 4♀ (TARI), Tienchih (天池), 1.IV.2015, leg. C.-F. Lee; Nantou: 1♂ (TARI), Hohuanhsi trail (合歡溪步道), 15.V.2017, leg. J.-C. Chen; 3♂ (TARI), same (= Huakang, 華岡) but with “23.IV.2019”; 1♂, 3♀ (TARI), same but with “24.IV.2021”; 3♂, 1♀ (TARI), Kunyang (昆陽), 17.IV.2021, leg. W.-C. Liao; 1♂, 2♀ (NMNS), Nanhuashan (南華山), 6.V.1992, leg. W.-T. Yang & K.-W. Huang; 16♀ (TARI), Tatachia (塔塔加), 9.VI.2009, leg. T.-H. Lee; 2♂, 5♀ (TARI), same locality, 21.IX.2009, leg. C.-F. Lee; 11♂, 5♀ (TARI), same but with “leg. M.-H. Tsou”; 1♀ (TARI), same locality, 30.X.2009, leg. C.-F. Lee; 3♀ (TARI), same but with “13.V.2015”; Taichung: 1♂ (TARI), Hsuehshan (雪山), 26.VI.2017, leg. W.-B. Yeh.

#### Metallic blue form

**(*n* = 17)**: Taiwan. Hualien: 2♀ (TARI), Malichiananshan (馬利加南山), 2.VI.2020, leg. J.-C. Chen; 1♀ (TARI), Mapolassushan (馬博拉斯山), 31.V.2020, leg. J.-C. Chen; 1♂ (TARI), Tayuling (大禹嶺), 12–15.IX.1980, leg. K. S. Lin & C. H. Wang; Nantou: 1♀ (KMNH), Hehuanshan (合歡山), 7.IX.1986, leg. K. baba; 1♀ (TARI), same locality, 23.VI.2018, leg. H.-F. Lu; 1♀ (NMNS), Hsiaofengko (小風口), 23.VI.–24.VIII.2009, leg. W.-T. Yang & K.-W. Huang; 1♂ (NMNS), same but with “24.IX.–22.X.2009”; 1♀ (TARI), Huakang (華岡), 24.IV.2019, leg. J.-C. Chen; 1♀ (HTC), Tsuifeng (翠峰), 20–21.VII.1995, leg. H. Takizawa; Taichung: 1♀ (TARI), Hsuehshan (雪山) 1.IV.2010, leg. W.-B. Yeh; 2♀ (TARI), same but with “7–8.IV.2011”; 1♀ (TARI), same but with “10.VI.2011”; 1♀ (TARI), same but with “8.X.2011”; 2♀ (TARI), same locality, 1.V.2012, leg. T.-H. Lee; Taitung: 1♀ (TARI), Hsiangyangshan (向陽山), 19.VI.2014, leg. J.-C. Chen; 1♂ (TARI), same but with “6.VIII.2015”.

#### Diagnosis.

Adults of *Arthrotussaigusai* Kimoto are similar to those of *A.tricolor* (Chûjô) and *A.fulvus* Chûjô in possessing rounded lateral margins of pronota (straight lateral margins of pronotum in *A.abdominalis* (Chûjô), *A.gressitti* Kimoto, *A.hirashimai* Kimoto, and *A.yuae* sp. nov.), the less transverse pronotum and elytra, 1.7–2.0 × wider and long in pronotum and 1.5–1.6 × longer than wide in elytra (the more transverse pronotum and elytra, 2.1–2.2 × wider and long and 1.4 × longer than wide in elytra of *A.testaceus* Gressitt & Kimoto and *A.yangi* sp. nov.). Adults of *A.saigusai* are different from those of *A.fulvus* and *A.tricolor* by the less transverse antennomere III in male, 1.1 × longer than wide (Fig. 16A) (the more transverse antennomere III in male, 0.7–0.8 × longer than wide in *A.fulvus* (Fig. 12A) and *A.tricolor* (Fig. 20A), the less slender antennae, antennomeres IV–VI 3.0–3.5 × in male longer than wide and VII–XI in male and IV–XI in female < 4.3 × longer than wide (Fig. 16A, B) (more slender antennae, antennomeres IV–VI in male > 3.7 × longer than wide, VII–XI in male and IV–XI in female > 4.3 × longer than wide in *A.fulvus* (Fig. 12A, B) and *A.tricolor* (Fig. 20A, B), the slightly curved apex of aedeagus (Fig. 16D) (the recurved apex of aedeagus in *A.fulvus* (Fig. 12D) and *A.tricolor* (Fig. 20D)).

#### Redescription.

Pale individuals have yellowish brown bodies with black antennae except antennomeres I, tibiae, and tarsi (Fig. 14A, B), some with vertex darker. Pronotum with median longitudinal wide black stripe from base to apex; scutellum black; thoracic ventrites blackish brown except hypomeron (Fig. 14C, D). Some similar to the previous form but differing in possessing three pairs of black spots on the elytra: one pair near base, two pairs on the transverse line at middle (Fig. 14E); some with longitudinal black stripes instead of black spots (Fig. 14F). Metallic blue individuals occurring in alpine habitats with entirely metallic blue bodies (Fig. 15A–D). One individual with metallic elytra but with blackish brown body. Pronotum with median transverse depression; shiny, without reticulate microsculpture; with sparse, coarse, confused punctures; lateral margins rounded, widest at middle; apical and basal margins slightly concave. Elytra with rounded lateral margin, widest at apical 1/3; disc shiny, without reticulate microsculpture, and with dense, coarse punctures (Fig. 15E, F).

**Male.** Length 5.0–5.2 mm, width 2.4–2.5 mm. Antennae filiform (Fig. 16A), antennomere III less modified, shorter than II, IV–VII relatively wider, length ratios of antennomeres I–XI 1.0: 0.4: 0.3: 1.1: 1.0: 1.0: 1.0: 1.0: 1.0: 0.9: 1.1, length to width ratios of antennomeres I–XI 3.2: 1.5: 1.1: 3.4: 3.0: 3.5: 3.7: 3.9: 4.2: 4.0: 4.3. Pronotum 1.9 × wider than long. Elytra 1.6 × longer than wide. Aedeagus (Fig. 7) extremely slender, ~ 11.3 × longer than wide, parallel-sided, slightly narrowed at apical 1/4, basally widened, apex narrowly rounded; tectum membranous, covered with weakly sclerotized, tiny setae; weakly curved in lateral view, apex curved and narrowly rounded; primary endophallic sclerite elongate, ~ 0.5 × as long as aedeagus, apex trilobed, with a cluster of dense setae near apex, and tiny teeth above clustered setae; deeply bifurcate from middle to base.

**Female.** Length 5.9–6.5 mm, width 3.0–3.3 mm. Antennae (Fig. 16B) much more slender than in males, antennomere III a little longer than II, length ratios of antennomeres I–XI 1.0: 0.4: 0.5: 1.0: 0.9: 0.9: 0.9: 0.9: 0.9: 0.9: 1.0, length to width ratios of antennomeres I–XI 3.5: 1.8: 2.0: 4.1: 3.5: 3.8: 4.0: 3.9: 4.2: 4.2: 4.2. Pronotum 1.8 × wider than long. Elytra 1.6 × longer than wide. Ventrite VIII (Fig. 16F) weakly sclerotized, apical margin widely rounded, with sparse, short setae along apical margin, and sparse, long setae at inner transverse row; spiculum extremely slender. Receptacle of spermatheca (Fig. 16G) slightly swollen, divided from pump; pump narrow and moderately curved, apex narrowly rounded; sclerotized proximal spermathecal duct wide and short, shallowly projecting into receptaculum. Gonocoxae (Fig. 16E) connected at one point, ~ 5.0 × longer than wide, curved inwards apically, with one short seta at apical 1/3, eight additional setae at apical areas.

#### Food plants.

Leaves of *Reynoutriajaponica* Houtt. (Polygonaceae). Adults were found hiding inside the curled tender leaves of the food plants (Fig. 17A, B).

#### Remarks.

One specimen collected from Ho Huan Shan (合歡山) by K. Baba was misidentified as *Arthrotusfulvus* by Kimoto (1989).

#### Distribution.

Adults of *Arthrotussaigusai* Kimoto are widespread in high-elevations (above 2500 m) of Taiwan (Fig. 18).

### ﻿ Arthrotus tricolor

Taxon classificationAnimaliaColeopteraChrysomelidae

(Chûjô, 1965)

6FE995F4-D786-575B-9ED4-9A4C67232496

**Figure 19.**
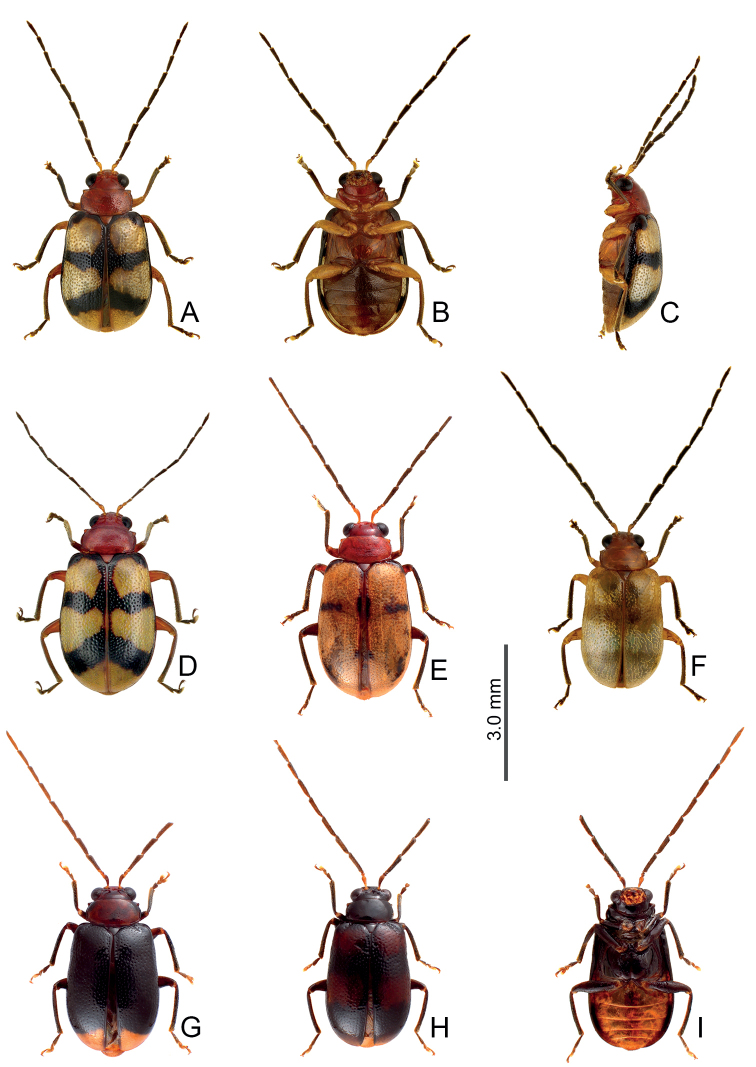
Habitus of *Arthrotustricolor* (Chûjô) **A** collected from Chutzuhu (竹子湖), male, dorsal view **B** collected from Chutzuhu (竹子湖), male, ventral view **C** collected from Chutzuhu (竹子湖), male, lateral view **D** collected from Chutzuhu (竹子湖), female, dorsal view **E** collected from Junghua (榮華), male, dorsal view **F** collected from Wulai (烏來), male, dorsal view **G** collected from Lilungshan (里龍山), male, dorsal view **H** collected from Wushihkeng (烏石坑), male, dorsal view **I** ditto, ventral view.

**Figure 20.**
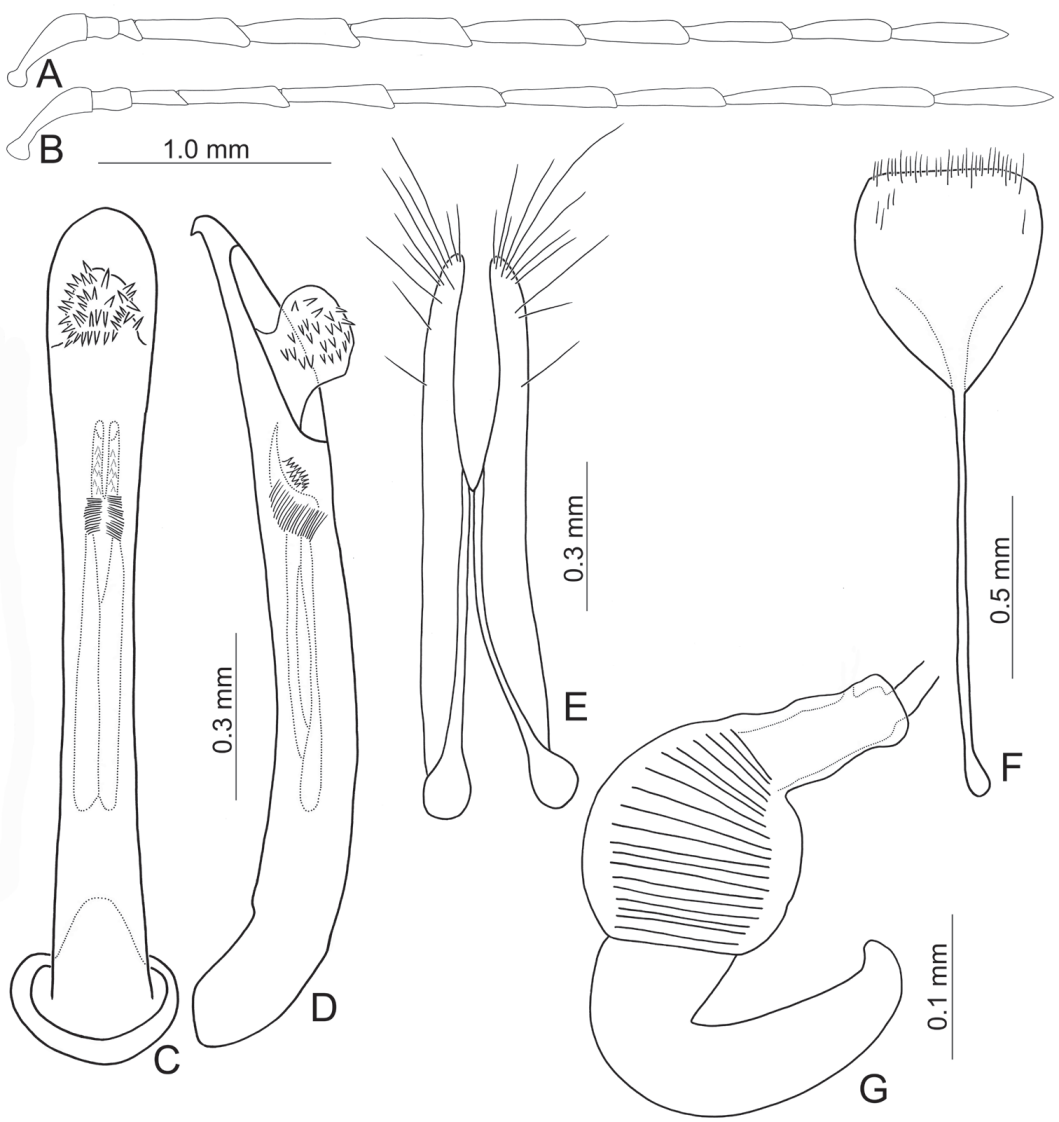
Diagnostic characters of *Arthrotustricolor* (Chûjô) **A** antenna, male **B** antenna, female **C** aedeagus, dorsal view **D** aedeagus, lateral view **E** gonocoxae **F** abdominal ventrite VIII **G** spermatheca.

**Figure 21.**
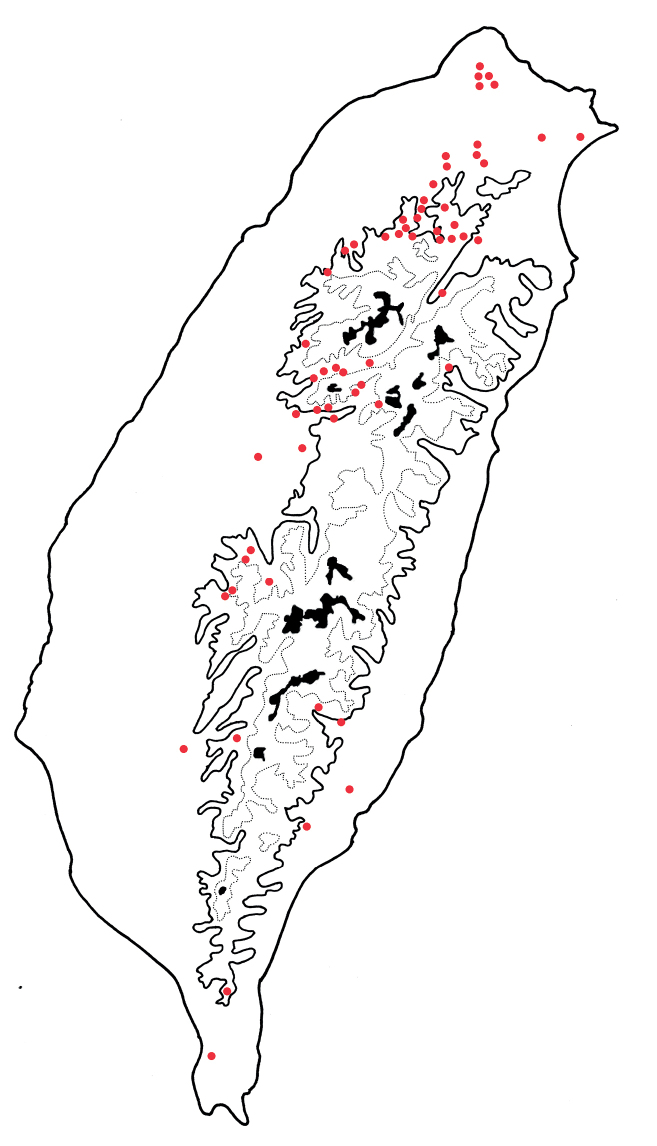
Distribution map of *Arthrotustricolor* (Chûjô), solid line: 1000 m, broken line: 2000 m, black areas: 3000 m.

Figs 17C, D , 19 , 20 , 21

Arthrotus fulvus Chûjô, 1938: 139 (part).
Dercetis tricolor Chûjô, 1965: 95.
Dercetina tricolor : Kimoto 1989: 280 (additional records).
Arthrotus tricolor : Kimoto 1969: 60 (transferred from Dercetis); Kimoto 1986: 58 (additional records); Kimoto 1991: 17 (additional records).

#### Types.

***Holotype*** ♀ (KUEC): “Rimogan (= Fushan, 福山) / Formosa / 10.VII.1961 / Coll. T. SHIROZU [h, w] // Dercetis / tricolor / Chûjô, ♀ [h] / Det. M. CHUJO, 196[p]2[h, w]”.

#### Other material.

A total of 299 specimens was examined (Suppl. material 3).

#### Diagnosis.

Adults of *Arthrotustricolor* (Chûjô) are similar to those of *A.fulvus* Chûjô in possessing rounded lateral margins of pronota (straight lateral margins of pronotum in *A.abdominalis* (Chûjô), *A.gressitti* Kimoto, *A.hirashimai* Kimoto, and *A.yuae* sp. nov.), the less transverse pronotum and elytra, 1.7–2.0 × wider and long in pronotum and 1.5–1.6 × longer than wide in elytra (more transverse pronotum and elytra, 2.1–2.2 × wider and long and 1.4 × longer than wide in elytra of *A.testaceus* Gressitt & Kimoto and *A.yangi* sp. nov.), a more transverse antennomere III in male, 0.7–0.8 × longer than wide (Fig. 20A) (less transverse antennomere III in male, 1.1 × longer than wide in *A.saigusai* Kimoto (Fig. 16A)). Adults of *A.tricolor* are different from those of *A.fulvus* (Figs 9–11) based on their characteristic color patterns (Fig. 19), tectum of aedeagus without pairs of apical tube-like processes and disc covered with clustered stout setae (Fig. 20C, D) (with one pair of apical tube-like processes and disc covered with scattered short setae in *A.fulvus* (Fig. 12C, D)).

#### Redescription.

Color yellowish brown; head, scutellum, and prothorax reddish brown, but antennae black; elytra with black stripes along basal margin, extending along entire suture, and lateral margins from base to apical 1/3, with two transverse black stripes at basal 1/3 and apical 1/3, legs black (Fig. 19A–D). Some specimens have reduced or paler black stripes on the elytra (Fig. 19E, F), some have black stripes expanding and the entire elytra black except apical 1/3 (Fig. 19G). Pronotum with median transverse depression reduced; shiny, without reticulate microsculpture; with sparse, coarse punctures confused; lateral margins rounded, widest at middle; apical and basal margins slightly concave. Elytra parallel-sided, disc shiny, without reticulate microsculpture, and with dense, coarse punctures.

**Male.** Length 5.2–5.6 mm, width 2.4–2.8 mm. Antennae filiform (Fig. 20A), antennomere III modified, much shorter than II, IV–VII relatively wider, length ratios of antennomeres I–XI 1.0: 0.3: 0.2: 1.0: 1.1: 1.1: 1.2: 1.1: 1.0: 1.0: 1.2, length to width ratios of antennomeres I–XI 3.5: 1.5: 0.8: 3.7: 4.0: 4.0: 4.3: 4.7: 5.1: 4.8: 6.2. Pronotum 1.9–2.0 × wider than long. Elytra parallel-sided, 1.4–1.6 × longer than wide. Aedeagus (Fig. 20C, D) extremely slender, ~ 12.5 × longer than wide, parallel-sided, slightly narrowed at apical 1/4, basally widened, apex widely rounded; tectum membranous, strongly swollen, covered with densely, well-sclerotized, stout setae; weakly curved in lateral view, apex recurved; primary endophallic sclerite elongate, ~ 0.5 × as long as aedeagus, apex bifurcate, with a cluster of dense setae near apex, and tiny teeth above clustered setae; deeply bifurcate from middle to base.

**Female.** Length 5.4–6.4 mm, width 3.1–3.8 mm. Antennae (Fig. 20B) much slender than in males, antennomere III a little longer than II, length ratios of antennomeres I–XI 1.0: 0.4: 0.5: 1.0: 1.1: 1.1: 1.1: 1.1: 1.1: 1.0: 1.2, length to width ratios of antennomeres I–XI 3.9: 1.9: 3.4: 5.1: 4.6: 5.7: 5.7: 5.6: 5.4: 5.3: 6.3. Pronotum 1.9–2.0 × wider than long. Elytra 1.4 × longer than wide. Ventrite VIII (Fig. 20F) weakly sclerotized, apically truncate, with dense, short setae along apical margin, and sparse, long setae at inner transverse line; spiculum extremely slender. Receptacle of spermatheca (Fig. 20G) strongly swollen, divided from pump; pump narrow and moderately curved, apex narrowly rounded; sclerotized proximal spermathecal duct wide and short, shallowly projecting into receptaculum. Gonocoxae (Fig. 20E) connected at middle at one point, ~ 5.2 × longer than wide, curved inwards at apical 1/3, with one long seta at apical 1/3, nine or ten additional setae at apical areas.

#### Variations.

Some specimens collected from Tahsuehshan (大雪山) and nearby Wushihkeng (烏石坑) have a distinctive color form (Fig. 17C, D): body almost black except yellowish brown abdomen and yellow spots on the elytra, as typical form (Fig. 19H, I)

#### Remarks.

Two types of *Arthrotusfulvus* [1♀ (TARI): “Formosa / Karenko (= Hualien, 花蓮), -19 / VII 20-VIII 4. / T. Okumi, // CO / Type [p, w, circle label with yellow letters and yellow border] // Arthrotus / fulvus / Chûjô [h] / DET. M. CHUJO [p, w] // 2588 [p, w]” and 1♀ (TARI): “Kuaru (= Kueitzuchiao, 龜子角) [h] / FORMOSA [p] / 12.VI.1937 [h] / COL. M. CHUJO [p, w]”; CO / Type [p, w, circle label with yellow letters and yellow border] // Arthrotus / fulvus / Chûjô [h] / DET. M. CHUJO [p, w] // 1370 [p, w]”] are misidentified. They should belong to *A.tricolor*.

#### Food plants.

Leaves of Styraxformosanusvar.formosanus Matsum. (Styracaceae), *Cryptocaryachinensis* (Hance) Hemsl. (Lauraceae), and *Actinidiarufa* (Siebold & Zucc.) Planch. ex Miq. (Actinidiaceae).

#### Distribution.

Adults of *Arthrotustricolor* (Chûjô) are widespread in lowlands (below 1500 m) of Taiwan. They are more common in northern Taiwan (Fig. 21).

### ﻿ Arthrotus testaceus

Taxon classificationAnimaliaColeopteraChrysomelidae

Gressitt & Kimoto, 1963

0AFCA5D2-60D7-5441-8C6D-607AD0B8185D

**Figure 22.**
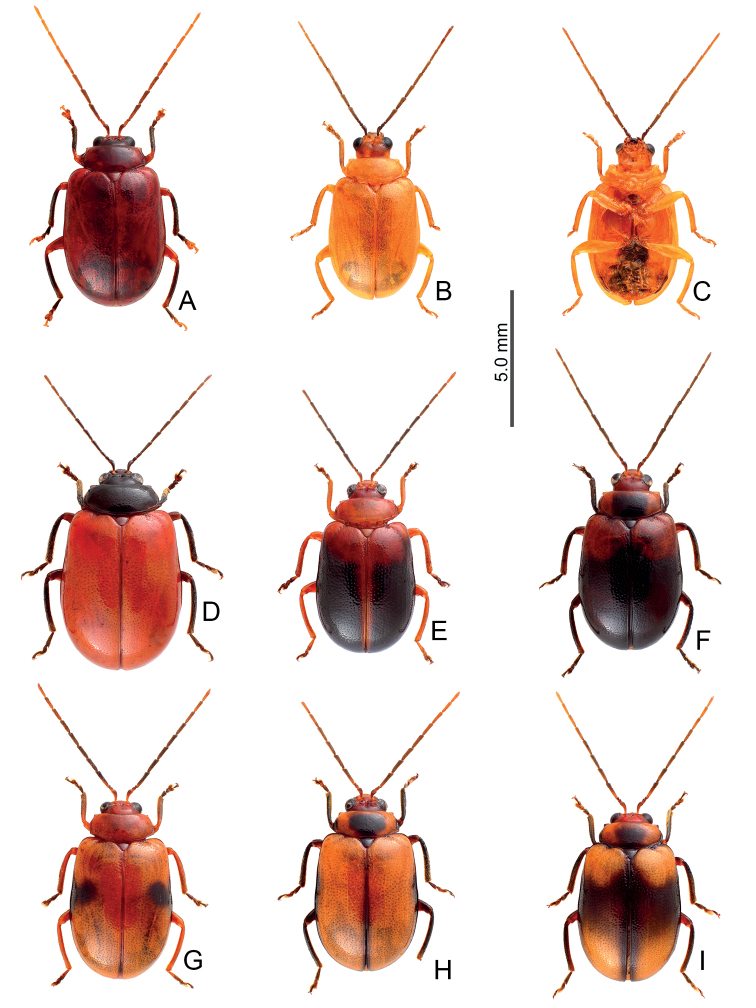
Habitus of *Arthrotustestaceus* Gressitt & Kimoto **A** collected from Tengchih (藤枝), male, dorsal view **B** collected from Peitawushan (北大武山), male, dorsal view **C** ditto, ventral view **D** collected from Tahanshan (大漢山), female, dorsal view **E** collected from Tahanshan (大漢山), male, dorsal view **F** collected from Tahanshan (大漢山), male, dorsal view **G** collected from Tahanshan (大漢山), male, dorsal view **H** collected from Tahanshan (大漢山), male, dorsal view **I** collected from Tahanshan (大漢山), male, dorsal view.

**Figure 23.**
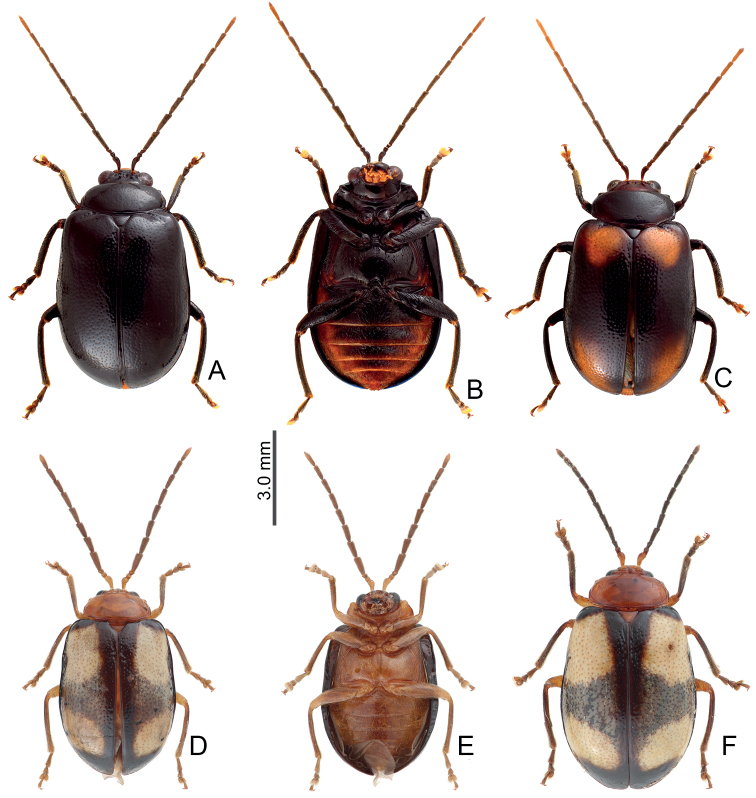
Habitus of *Arthrotustestaceus* Gressitt & Kimoto and *A.yangi* sp. nov. **A***A.testaceus*, collected from Tahanshan (大漢山), male, dorsal view **B***A.testaceus*, collected from Tahanshan (大漢山), male, ventral view **C***A.testaceus*, collected from Tahanshan (大漢山), male, dorsal view **D***A.yangi* sp. nov., collected from Hualuhsi (華綠溪), male, dorsal view **E***A.yangi* sp. nov., collected from Hualuhsi (華綠溪), male, ventral view **F***A.yangi* sp. nov., collected from Hualuhsi (華綠溪), female, dorsal view.

**Figure 24.**
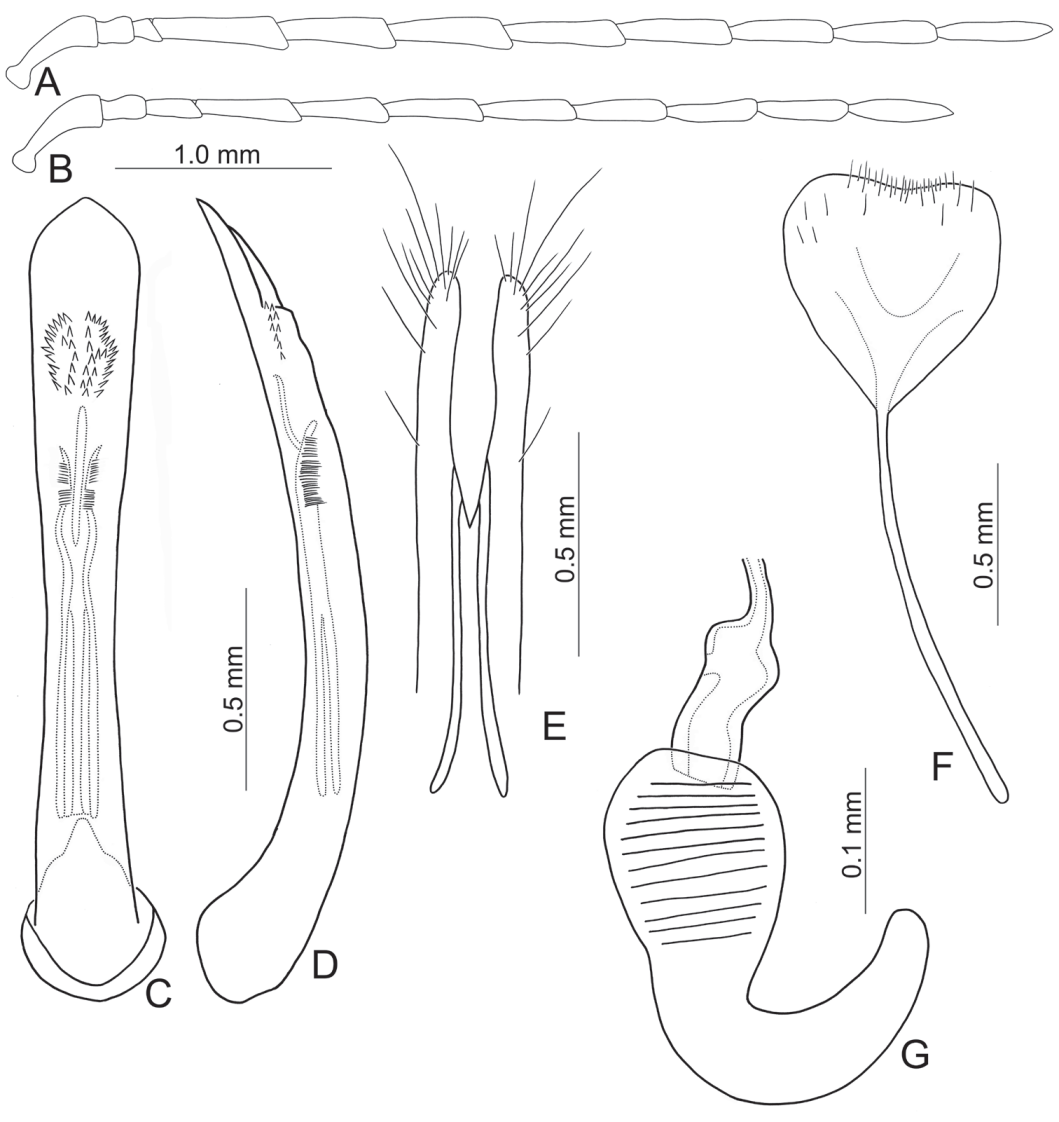
Diagnostic characters of *Arthrotustestaceus* Gressitt & Kimoto **A** antenna, male **B** antenna, female **C** aedeagus, dorsal view **D** aedeagus, lateral view **E** gonocoxae **F** abdominal ventrite VIII **G** spermatheca.

**Figure 25.**
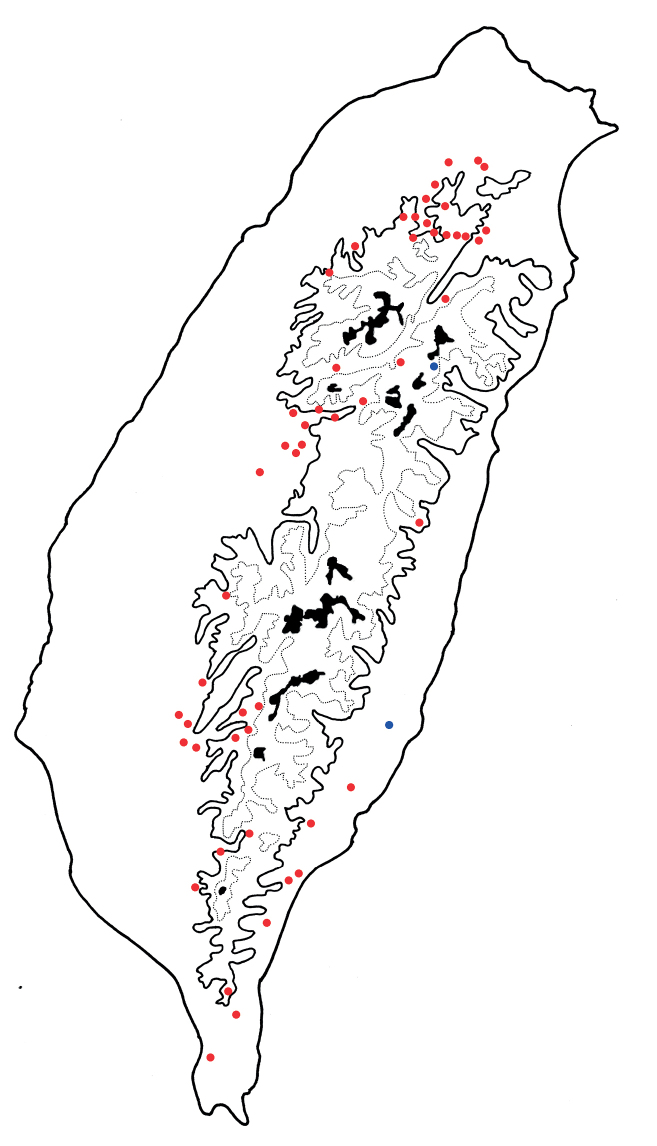
Distribution map of *Arthrotustestaceus* Gressitt & Kimoto and *A.yangi* sp. nov., solid line: 1000 m, broken line: 2000 m, black areas: 3000 m. Key: red dots *A.testaceus*, blue dots *A.yangi* sp. nov.

Figs 22 , 23A–C , 24 , 25

Arthrotus testaceus Gressitt & Kimoto, 1963: 702 (China); Kimoto 1969: 60 (Taiwan).
Arthrotus shibatai Kimoto, 1984: 55 (Taiwan); Kimoto 1989: 259 (additional records); Kimoto 1991: 16 (additional records). syn. nov.

#### Types.

*Arthrotustestaceus*. ***Paratypes*.** 1♂ (KMNH): “W. HUPEH, China / Lichuan, Hsiaoho / VIII[p]-10-[h]1948 [p, w] // Gressitt & / Djou Collrs. [p, w] // Arthrotus / testaceus / G & K [h] / Gressitt & Kimoto det. 196[p]2 [p, w] // PARATYPE [p, b]”; 1♂ (KMNH): “CHINA, / W. HUPEH, / Sulho, Lichuan / IX-14-1948 [p, w] // Gressitt & / Djou Collrs. [p, w] // Arthrotus / testaceus / G & K [h] / Gressitt & Kimoto det. 196[p]2 [p, w] // PARATYPE [p, b]”; 1♂ (BPBM): “Suisapa, 1000 M. / Lichuan Distr. / W. Hupeh, China / VIII- [p] 21 [h] -48 [p, w] // J. L. Gressitt / Collector [p, w] // PARATYPE [p] / Arthrotus / testaceus [h] / Gressitt & Kimoto [p, y] // Arthrotus / testaceus / G & K [h] / Gressitt & Kimoto det. 196[p]2 [p, w]”; 1♀ (CAS), same but with “VIII- [p] 22 [h] -48 [p, w]”; 1♂ (CAS), same but with one additional card, labeled: “Ex / Liquidamo. / formosana [p, w]”; 1♀ (CAS), same but with “VIII- [p] 23 [h] -48 [p, w]”; 1♀ (CAS), same but with “VIII- [p] 25 [h] -48 [p, w]”; 1♂ (CAS), same but with “VIII- [p] 30 [h] -48 [p, w]”; 1♂ (BPBM): “FUKIEN, S. China / Chungah: Upper / Kuatun 1400 m. / T. C. Maa [p, w] // Apr.12.1943 [h, w] // PARATYPE [p] / Arthrotus / testaceus [h] / Gressitt & Kimoto [p, y] // Arthrotus / testaceus / G & K [h] / Gressitt & Kimoto det. 196[p]2 [p, w]”; 1♂ (CAS): “Mokansan China / Che Kiang Pr. [p] / IX-18-27 [h, w] // Mrs. Dora / E. Wright / Collector [p, w] // PARATYPE [p] / Arthrotus / testaceus [h] / Gressitt & Kimoto [p, y] // Arthrotus / testaceus / G & K [h] / Gressitt & Kimoto det. 196[p]2 [p, w]”.

*Arthrotusshibatai*. ***Holotype*** ♂ (OMNH): “NANSHANCHI (南山溪) / TAIWAN / 1.IV.1981 / F. KIMURA [p, y] // ♂ [p, w, with black border] // Arthrotus / shibatai / Kimoto [h, w] // HOLOTYPE [p, r]”. Paratypes: 1♀ (KMNH): “NANSHANCHI (南山溪) / TAIWAN / 31.III.1981 / F. KIMURA [p, y] // PHOTO [p, r]”; 1♂ (KMNH): “NANSHANCI [sic!] (南山溪) / TAIWAN / 3.IV.1971 / H. NOMURA [p, y] // ♂ [p, w, with black border]”; 1♀ (KMNH): “APR 9. 1967 / 松安 (= Sungan) / B-S. CHANG [h, w]”; 1♀ (KMNH): “(Taiwan) / Huanshan (環山) / Hsuehshan Mo (雪山山脈) / Taichung Hs. [p, w] // Jun [p] 1 [h] .1971 / K Kanmiya [p, w]”. Each paratype bears two additional labels: “Arthrotus / shibatai / Kimoto [h, w] // PARATYPE [p, b]”.

#### Other material.

A total of 301 specimens was examined (Suppl. material 4).

#### Diagnosis.

**Figure 26.**
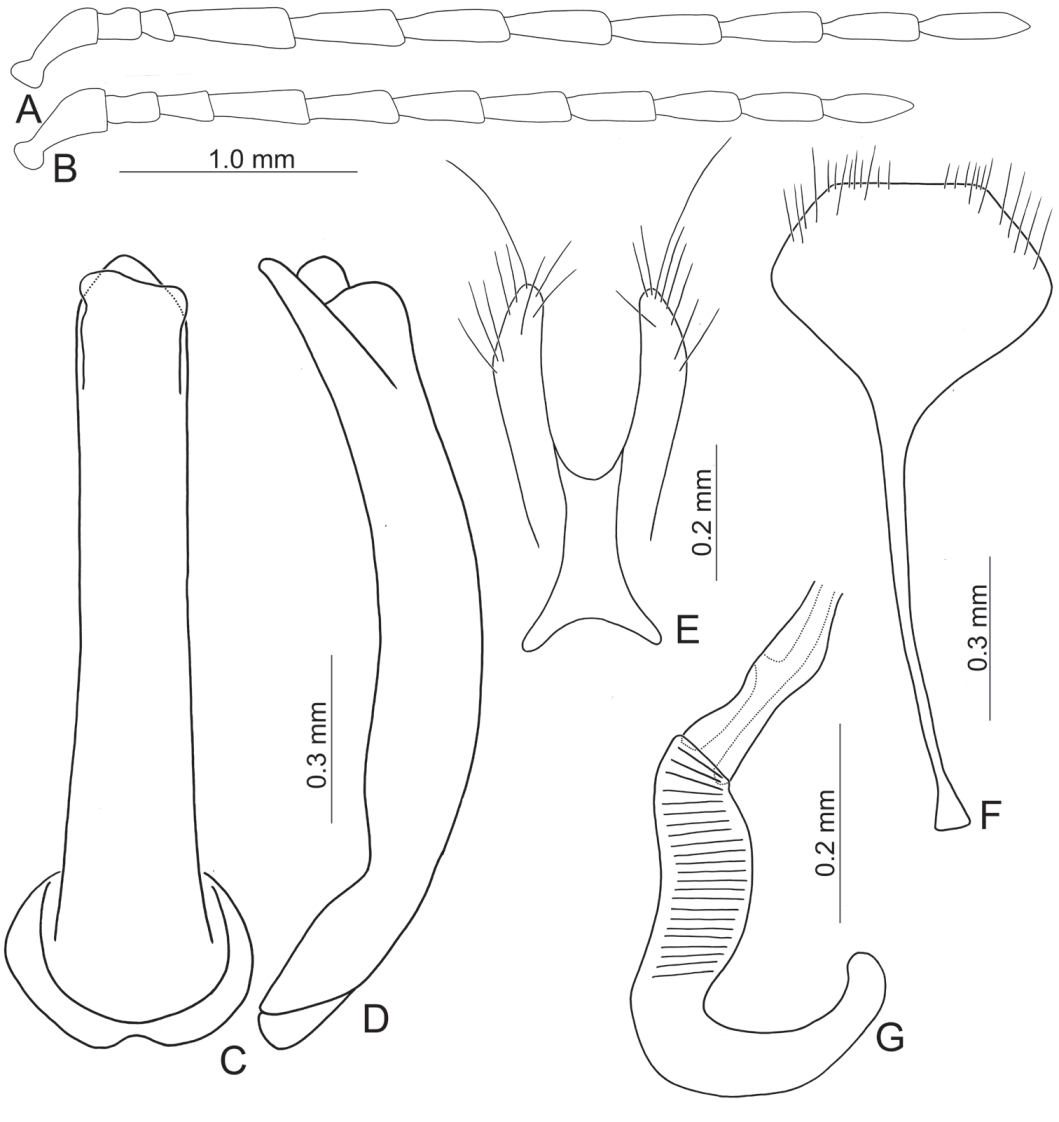
Diagnostic characters of *Arthrotusyangi* sp. nov. **A** antenna, male **B** antenna, female **C** aedeagus, dorsal view **D** aedeagus, lateral view **E** gonocoxae **F** abdominal ventrite VIII **G** spermatheca.

Adults of *Arthrotustestaceus* Gressitt & Kimoto (Figs 22, 23A–C) and *A.yangi* sp. nov. (Fig. 23D–F) are characterized by the more transverse pronotum and elytra, 2.1–2.2 × wider and long in pronotum and 1.4 × longer than wide in elytra (less transverse pronotum and elytra, 1.7–2.0 × wider and long in pronotum and 1.5–1.6 × longer than wide in elytra of others). Adults of *A.testaceus* (Figs 22, 23A–C) are different from *A.yangi* sp. nov. in lacking the characteristic color pattern of *A.yangi* sp. nov. (Fig. 23D–F); more slender antennae, antennomeres IV–VII 3.6–4.2 × longer than wide and VII–X 4.5–4.8 × longer than wide in males (Fig. 24A), IV–X > 4.0 × longer than wide in females of *A.testaceus* (Fig. 24B) (less slender antennae, antennomeres IV–VII 2.9–3.1 × longer than wide and VIII–X 3.4–3.7 × longer than wide in males (Fig. 26A), IV–X < 3.0 × longer than wide in females of *A.yangi* sp. nov. (Fig. 26B)); more slender aedeagus, 11.3 × longer than wide (Fig. 24C, D) (less slender aedeagus, 8.0 × longer than wide in *A.yangi* sp. nov. (Fig. 26C, D)), tectum with clustered stout setae (Fig. 24A) (tectum with setae almost reduced in *A.yangi* sp. nov. (Fig. 26C)); slender gonocoxae, 4.8 × longer than wide (Fig. 24E) (wide gonocoxae, 1.9 × longer than wide in *A.yangi* sp. nov. (Fig. 26E)); and slightly swollen receptacle of spermathecal (Fig. 24G) (slender swollen receptacle of spermatheca in *A.yangi* sp. nov. (Fig. 26G)).

#### Redescription.

Adults from China yellowish brown, but extremely variable in Taiwan, some similar to those of China (Fig. 22B, C) reddish brown with black antennae (except three basal antennomeres), tibiae, and tarsi (Fig. 22A); some with dark brown or black heads and pronota (Fig. 22D); some with black elytra but base and suture not darkened (Fig. 22E); some with base of elytra not darkened but pronotum with wide longitudinal black band (Fig. 22F); some with black lateral margin on elytra starting from basal 1/3, extending inwards at basal 1/3 (Fig. 22G); some with centrally darkened pronotum, elytra with black suture and lateral margin starting from basal 1/3, and black scutellum, and dark brown head (Fig. 22H); some similar to previous forms but with wide transverse dark band at basal 1/3 of elytra (Fig. 22I). Some with black body form with yellow abdomens (Fig. 23A, B); some with two additional, large, pale spots on elytra, one pair near base, the other near apex (Fig. 23C). Pronotum without median transverse depression; shiny, without reticulate microsculpture; with sparse, coarse punctures confused; lateral margins rounded, widest at middle; apical and basal margins strongly concave. Elytra parallel-sided, disc shiny, without reticulate microsculpture, and with dense, coarse punctures.

**Male.** Length 6.5–7.1 mm, width 3.9–4.0 mm. Antennae filiform (Fig. 24A), antennomere III shorter than II, IV–VII relatively wider, length ratios of antennomeres I–XI 1.0: 0.4: 0.2: 1.3: 1.1: 1.2: 1.2: 1.1: 1.0: 1.0: 1.1, length to width ratios of antennomeres I–XI 3.4: 1.3: 0.9: 3.9: 3.6: 3.8: 4.2: 4.5: 4.5: 4.8: 6.0. Pronotum 2.2 × wider than long. Elytra 1.4 × longer than wide. Aedeagus (Fig. 24C, D) extremely slender, ~ 11.3 × longer than wide, slightly narrowed medially, basally widened, apex narrowly rounded; tectum membranous, covered with a pair of clusters of stout setae; moderately curved in lateral view, apex curved and acute; primary endophallic sclerite elongate, ~ 0.5 × as long as aedeagus, apex trilobed, with a cluster of dense setae near apex; deeply bifurcate from middle to base.

**Female.** Length 7.2–7.9 mm, width 4.3–4.4 mm. Antennae (Fig. 24B) much more slender than in males, antennomere III a little longer than II, length ratios of antennomeres I–XI 1.0: 0.4: 0.5: 0.9: 0.9: 0.9: 0.9: 0.9: 0.9: 0.9: 1.0, length to width ratios of antennomeres I–XI 3.5: 1.8: 2.6: 4.2: 4.0: 4.2: 4.4: 4.6: 4.5: 4.5: 4.6. Pronotum 2.2 × wider than long. Elytra 1.4 × longer than wide. Ventrite VIII (Fig. 24F) weakly sclerotized, with dense, short setae along apical margin, and sparse, long setae at inner transverse row; spiculum extremely slender. Receptacle of spermatheca (Fig. 24G) strongly swollen, divided from pump; pump narrow and moderately curved, apex narrowly rounded; sclerotized proximal spermathecal duct wide and short, shallowly projecting into receptaculum. Gonocoxae (Fig. 24E) connected at one point, ~ 4.8 × longer than wide, curved inwards apically, with one short seta at apical 1/3, nine or ten additional setae apically.

#### Food plants.

Leaves of *Sapindusmukorossi* Gaertn. (Sapindaceae), AcerinsulareHayatavar.caudatifolium (Hayata) S.Y. Lu & Y.P. Yang (Sapindaceae), *Aceralbopurpurascens* Hayata (Sapindaceae), *Alniphyllumpterospermum* Matsum. (Styracaceae), *Alnusformosana* (Burkill) Makino (Betulaceae), and *Lithocarpushancei* (Benth.) Rehder (Fagaceae).

#### Distribution.

Adults of *Arthrotustestaceus* Gressitt & Kimoto are widespread in lowlands (below 1500 m) of Taiwan (Fig. 25).

### ﻿ Arthrotus yangi sp. nov.

Taxon classificationAnimaliaColeopteraChrysomelidae

1BA8DF8D-87C6-554C-9A44-B90C3E002D4D

http://zoobank.org/CD028F70-32D7-4825-960B-AA39A8E78073

Figs 23D–F , 25 , 26

#### Type series

**(*n* = 3). *Holotype*** ♂ (NMNS): Taiwan. Hualien: Hualuhsi (華綠溪), 19.IV.-1.VI.2011, leg. W.-T. Yang & K. W. Huang, with Malaise trap. ***Paratypes*.** 1♀ (NMNS), same data as holotype; 1♀ (NMNS): Taiwan. Taitung: Shinkangshan (新港山), 24.III.-19.V.2009, leg. W.-T. Yang & K. W. Huang, with Malaise trap.

#### Diagnosis.

Adults of *Arthrotusyangi* sp. nov. (Fig. 23D–F) and *A.testaceus* Gressitt & Kimoto (Figs 22, 23A–C) are characterized by the more transverse pronotum and elytra, pronotum 2.1–2.2 × wider than long and elytra 1.4 × longer than wide (less transverse pronotum and elytra, pronotum 1.7–2.0 × wider than long and elytra 1.5–1.6 × longer than wide in others). Adults of *A.yangi* sp. nov. are different from *A.testaceus* based on the characteristic color pattern (Fig. 23D–F) (lacking characteristic color pattern in *A.testaceus* (Figs 22, 23A–C)); less slender antennae, antennomeres IV–VII 2.9–3.1 × longer than wide, and VIII–X 3.4–3.7 × longer than wide in males (Fig. 26A), IV–X < 3.0 × longer than wide in females (Fig. 26B) (more slender antennae, antennomeres IV–VII 3.6–4.2 × longer than wide and VII–X 4.5–4.8 × longer than wide in males (Fig. 24A), IV–X > 4.0 × longer than wide in females of *A.yangi* sp. nov. (Fig. 24B)); less slender aedeagus, 8.0 × longer than wide (Fig. 26C) (more slender aedeagus, 11.3 × longer than wide in *A.yangi* sp. nov. (Fig. 24C)), tectum with setae almost reduced (tectum with clustered stout setae in *A.yangi* sp. nov.); wide gonocoxae, 1.9 × longer than wide (Fig. 26E) (slender gonocoxae, 4.8 × longer than wide in *A.yangi* sp. nov. (Fig. 24E)); and slender receptacle of spermathecal (Fig. 26G) (slightly swollen swollen receptacle of spermatheca in *A.yangi* sp. nov. (Fig. 24G)).

#### Description.

Color (Fig. 23D–F) yellowish brown, antennae black except two basal tarsomeres; tibiae and tarsi darkened; elytra black, with two pairs of large white spots near base and at apical 1/3 respectively, areas mixed with white between anterior and posterior white spots. Pronotum without median transverse depression; shiny, without reticulate microsculpture; with sparse fine punctures; lateral margins rounded, widest at middle; apical margin strongly concave; basal margin truncate. Elytra with lateral margin slightly rounded, widest behind middle; disc without reticulate microsculpture, but with dense, coarse punctures.

**Male.** Length 5.5 mm, width 3.3 mm. Antennae filiform (Fig. 26A), antennomere III modified, much shorter than II, IV–VII much wider than others, length ratios of antennomeres I–XI 1.0: 0.4: 0.3: 1.2: 1.1: 1.1: 1.1: 1.1: 1.1: 1.0: 1.1, length to width ratios of antennomeres I–XI 2.6: 1.3: 0.9: 3.1: 2.9: 3.1: 3.0: 3.7: 3.5: 3.4: 4.5. Pronotum 2.2 × wider than long. Elytra 1.4 × longer than wide. Aedeagus (Fig. 26C, D) slender, ~ 8.0 × longer than wide, parallel-sided, apex widely rounded; tectum membranous, covered with extremely tiny and setae; moderately curved in lateral view, apex narrowly rounded; endophallic sclerites omitted.

**Female.** Length 6.7 mm, width 4.1 mm. Antennae (Fig. 26B) much shorter than in males, antennomere III subequal to II, IV–VI a little wider than others, length ratios of antennomeres I–XI 1.0: 0.5: 0.5: 0.9: 0.9: 0.8: 0.9: 0.8: 0.8: 0.8: 0.8, length to width ratios of antennomeres I–XI 2.2: 1.8: 1.7: 2.8: 2.6: 2.5: 2.1: 2.8: 2.9: 3.0: 3.3. Pronotum 2.1 × wider than long. Elytra 1.4 × longer than wide. Ventrite VIII (Fig. 4H) weakly sclerotized, apical margin truncate, with scattered long setae at sides, and several short setae at sides of apical margin; spiculum extremely slender. Receptacle of spermatheca (Fig. 26G) slightly swollen and slender, undivided from pump; pump selnder and moderately curved, apex broadly rounded; sclerotized proximal spermathecal duct wide and long, shallowly projecting into receptaculum. Gonocoxae (Fig. 26E) basally connected from middle, ~ 1.9 × longer than wide, with one long seta at apex, nine additional setae at apical areas.

#### Food plants.

Unknown.

#### Distribution.

Adults are collected from only two localities in East Taiwan (Fig. 25).

#### Etymology.

The specific name is dedicated to Mr Wan-Tsun Yang (楊萬琮) who collected type specimens using Malaise traps.

### ﻿Key to Taiwanese species of *Arthrotus*

**Table:** 

1	Lateral margins of pronotum straight; elytra metallic blue, with transverse depression at basal 1/ 3 (Figs 1, 6)	**2**
–	Lateral margins of pronotum rounded; color patterns of elytra variable, without transverse depression (Figs 9–11, 14, 15, 22, 23)	**5**
2	Head, pronotum, underside of thorax, and legs metallic blue (Fig. 1A–C)	***A.abdominalis* (Chûjô)**
–	Head, pronotum, underside of thorax, and legs blackish or yellowish brown (Figs 1D–F, 6)	**3**
3	Head, pronotum, underside of thorax, and legs blackish brown (Fig. 1D–F)	***A.gressitti* Kimoto**
–	Head, pronotum, underside of thorax, and legs yellowish brown (Fig. 6)	**4**
4	Tectum of aedeagus covered with short needle-shape setae laterally and stout teeth apically, apex curved in lateral view (Fig. 7C, D); central Taiwan, including south Nantou, and Chiayi counties (Fig. 5)	***A.hirashimai* Kimoto**
–	Tectum of aedeagus with scattered, stout setae, apex recurved in lateral view (Fig. 8C, D); south Taiwan, including Kaohsiung, Pingtung, and Taitung counties (Fig. 5)	***A.yuae* sp. nov.**
5	Pronotum and elytra more transverse, pronotum 2.1–2.2 × wider than long and elytra 1.4 × longer than wide (Figs 22, 23)	**6**
–	Pronotum and elytra less transverse, prontum1.7–2.0 × wider than and elytra 1.5–1.6 × longer than wide (Figs 9–11, 14, 15, 19)	**7**
6	Color pattern of elytra characteristic (Fig. 23D–F); antennae less slender, antennomeres IV–VII 2.9–3.1 × longer than wide and VIII–X 3.4–3.7 × longer than wide in males (Fig. 26A), IV–X < 3.0 × longer than wide in females (Fig. 26B)	***A.yangi* sp. nov.**
–	Color pattern on elytra variable but lacking above color pattern (Figs 22, 23A–C); antennae more slender, antennomeres IV–VII 3.6–4.2 × longer than wide and VII–X 4.5–4.8 × longer than wide in males (Fig. 24A), IV–X > 4.0 × longer than wide in females (Fig. 24B)	***A.testaceus* Gressitt & Kimoto**
7	Color pattern on elytra characteristic (Figs 14, 15); antennae less slender, antennomeres IV–VI 3.0–3.5 × longer than wide in male; VII–XI in male and IV–XI in female < 4.3 × longer than wide (Fig. 16A, B); antennomere III less transverse in male, 1.1 × longer than wide (Fig. 16A)	***A.saigusai* Kimoto**
–	Color pattern on elytra variable but lacking above color pattern (Figs 9–11, 19); antennae more slender, antennomeres IV–VI > 3.7 × longer than wide in male, VII–XI in male and IV–XI in female > 4.3 × longer than wide (Figs 12A, B, 20A, B); antennomere III in male more transverse, 0.7–0.8 × longer than wide (Figs 12A, 20A)	**8**
8	Color pattern on elytra characteristic (Fig. 19); tectum of aedeagus without pairs of apical tube-like processes and disc covered with clustered stout setae (Fig. 20C, D)	***A.tricolor* (Chûjô)**
–	Color pattern on elytra variable but lacking above color pattern (Figs 9–11); tectum of aedeagus with one pair of apical tube-like processes and disc covered with scattered short setae (Fig. 12C, D)	***A.fulvus* Chûjô**

## ﻿Discussion

Taxonomic studies on *Arthrotus* species of Taiwan are difficult due to females being confused with those of *Dercetina* and the great variation of color patterns of most species. Taiwanese species of *Dercetina* were revised recently (Lee and Bezdĕk 2013) and every species of *Arthrotus* can be delimited with sufficient material now. Five *Arthrotus* species (*A.abdominalis* (Chûjô), *A.gressitti* Kimoto, *A.hirashimai* Kimoto, *A.yuae* sp. nov., and *A.yangi* sp. nov.) have characteristic and consistent adult color patterns with one exception, *A.hirashimai* Kimoto and *A.yuae* sp. nov., which are identical but allopatric. Color patterns of *A.tricolor* (Chûjô) and *A.saigusai* Kimoto are variable but characteristic within each species. Color patterns of the other two species, *A.testaceus* Gressitt & Kimoto and *A.fulvus* Chûjô, are extremely variable, but they can be identified by body shape. Therefore, all Taiwanese species of *Arthrotus* can be recognized by color patterns, geographic distributions, and body shapes.

**Figure 27.**
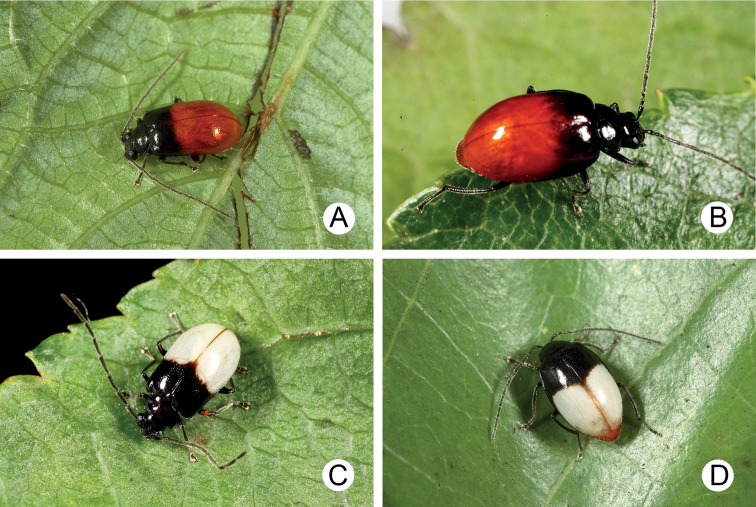
File photographs of *Arthrotusfulvus* Chûjô and *Neochyanitidissima* (Chûjô) **A** color form A of *A.fulvus* collected from Meifeng (梅峰) **B** similar color pattern of *N.nitidissima* collected from Kuanwu (觀霧) **C** color form B of *A.fulvus* collected from Tatachia (塔塔加) **D** similar color pattern of *N.nitidissima* collecte from Tatachia (塔塔加)

Five *Arthrotus* species are widespread in Taiwan, *A.abdominalis*, *A.testaceus*, *A.saigusai*, *A.tricolor*, and *A.fulvus*. Of these, only *A.saigusai* is alpine. Populations of *Arthrotusgressitti*, *A.hirashimai*, *A.yuae* sp. nov., and *A.yangi* sp. nov. are localized and have similar color patterns and body shapes but allopatric distributions. In addition, one interesting phenomenon was noticed, the two bicolored patterns (black and white, black and red) of *A.fulvus* (Fig. 27A, C) have similar distributions as *Neochyanitidissima* (Chûjô, 1935), which also presents these two color forms (Lee 2020) (Fig. 27B, D). Could such a phenomenon result from convergent evolution and occur to other chrysomelids? This question requires further studies.

## Supplementary Material

XML Treatment for Arthrotus abdominalis

XML Treatment for Arthrotus gressitti

XML Treatment for Arthrotus hirashimai

XML Treatment for Arthrotus yuae

XML Treatment for Arthrotus fulvus

XML Treatment for Arthrotus saigusai

XML Treatment for Arthrotus tricolor

XML Treatment for Arthrotus testaceus

XML Treatment for Arthrotus yangi

## Supplementary materials

Supplementary material 1 *Arthrotusabdominalis* (Chûjô, 1962) Data type docx file Explanation note *Arthrotusabdominalis* (Chûjô, 1962) https://binary.pensoft.net/file/667393This dataset is made available under the Open Database License (http://opendatacommons.org/licenses/odbl/1.0/). The Open Database License (ODbL) is a license agreement intended to allow users to freely share, modify, and use this Dataset while maintaining this same freedom for others, provided that the original source and author(s) are credited.Author: Chi-Feng Lee

Supplementary material 2 *Arthrotusfulvus* Chûjô, 1938 Data type docx file Explanation note *Arthrotusfulvus* Chûjô, 1938 https://binary.pensoft.net/file/667394This dataset is made available under the Open Database License (http://opendatacommons.org/licenses/odbl/1.0/). The Open Database License (ODbL) is a license agreement intended to allow users to freely share, modify, and use this Dataset while maintaining this same freedom for others, provided that the original source and author(s) are credited.Author: Chi-Feng Lee

Supplementary material 3 *Arthrotustricolor* (Chûjô, 1965) Data type docx file Explanation note *Arthrotustricolor* (Chûjô, 1965) https://binary.pensoft.net/file/667395This dataset is made available under the Open Database License (http://opendatacommons.org/licenses/odbl/1.0/). The Open Database License (ODbL) is a license agreement intended to allow users to freely share, modify, and use this Dataset while maintaining this same freedom for others, provided that the original source and author(s) are credited.Author: Chi-Feng Lee

Supplementary material 4 *Arthrotustestaceus* Gressitt & Kimoto, 1963 Data type docx file Explanation note *Arthrotustestaceus* Gressitt & Kimoto, 1963 https://binary.pensoft.net/file/667396This dataset is made available under the Open Database License (http://opendatacommons.org/licenses/odbl/1.0/). The Open Database License (ODbL) is a license agreement intended to allow users to freely share, modify, and use this Dataset while maintaining this same freedom for others, provided that the original source and author(s) are credited.Author: Chi-Feng Lee
