# Supplementary material for: ﻿Revision of the genus Arthrotus Motschulsky, 1858 (Coleoptera, Chrysomelidae, Galerucinae) of Taiwan, with notes on color polymorphism
Source: Zookeys. 2022 Apr 1;1091:161–208. doi: 10.3897/zookeys.1091.79486 (PMC9005468; doi:10.3897/zookeys.1091.79486)
Supplement: Supplementary material 1 — Arthrotusabdominalis (Chûjô, 1962) [file zookeys-1091-161-s001.docx]

**Supplementary file 1. *Arthrotus abdominalis* (Chûjô, 1962)**

**Other material (*n =* 316).** TAIWAN. Chiayi: 1♀ (TARI), Alishan (阿里山), 29.V.2016, leg. Y.-T. Chung; 1♂ (TARI), Chowushan (卓武山), 9.V.2019, leg. B.-X. Guo; 4♂ (TARI), Tulishan (獨立山), 1.III.2007, leg. S.-S. Li; 1♂ (TARI), Tutzuhu (杜仔湖), 14.III.2015, leg. W.-C. Liao; Hsinchu: 1♀ (TARI), Chenghsipao (鎮西堡), 16.V.2014, leg. Y.-F. Hsu; 1♀ (TARI), Litungshan (李棟山), 16.VI.2010, leg. Y.-L. Lin; 1♀ (TARI), Lupi (魯壁), 10.III.2009, leg. S.-F. Yu; 1♂ (TARI), same locality, 4.VI.2011, leg. M.-H. Tsou; 1♀ (TARI), same locality, 9.VIII.2014, leg. Y.-L. Lin; 1♀ (TARI), Shihlu trail (石鹿步道), 18.VII.2013, leg. Y.-L. Lin; Hualien: 1♂, 1♀ (TARI), Huitouwan (迴頭彎), 10.VII.2007, leg. C.-F. Lee; 1♂, 2♀ (TARI), Kuanyuan (關原), 7.V.2006, leg. Y.-F. Hsu; 1♂ (TARI), Loshao (洛韶), 19.II.2016, leg. J.-C. Chen; 1♀ (TARI), Pilu (碧綠), 17.V.2009, leg. C.-F. Lee; Ilan: 1♀ (TARI), Chilan (棲蘭), 17.III.2007, leg. M.-H. Tsou; 2♂ (TARI), same but with “leg. S.-F. Yu”; 1♀ (TARI), Chiuchihtse (鳩之澤), 7.XII.2008, leg. M.-H. Tsou; 2♂ (TARI), Fushan Botanical Park (福山植物園), 20.III.2009, leg. C.-F. Lee; 1♀ (TARI), Hsinliao (新寮), 19.I.2010, leg. S.-F. Yu; 3♀ (TARI), Mingchi (明池), 5.IV.2009, leg. M.-H. Tsou; 1♀ (NMNS), Tungshan (冬山), 17.IX.1992, leg. W.-T. Yang; Kaohsiung: 2♀ (TARI), Chiasien (甲仙), 10–13.V.1981, leg. C. C. Chen & C. C. Pan; 1♂ (TARI), Chungchihkuan (中之關), 17.IV.2012, leg. L.-P. Hsu; 2♂ (TARI), Erhchituan (二集團), 8.III.2013, leg. B.-X. Guo; 8♀ (TARI), Hsiaokuanshan (小關山), 15.V.2016, leg. B.-X. Guo; 1♂, 1♀ (HTC), Liukei (= Liukui, 六龜), 29.IV–9.V.1982, leg. H. Takizawa; 1♂, 2♀ (KMNH), 3.IV.1986, leg. K. Baba; 1♀ (KMNH), Tsaitiehku (?), near Liukui, 2.VI.1980, leg. K. Baba; 1♂ (TARI), Maolin (茂林), 28.III.1982, leg. K. C. Chou & C. C. Pan; 1♀ (TARI), Sanping (扇平), 22.III.2014, leg. W.-C. Liao; 1♀ (TARI), same but with “6.VI.2015”; 1♀ (TARI), Tengchih (藤枝), 1.V.2010, leg. U. Ong; 1♂ (TARI), same locality, 29.V.2013, leg. Y.-T. Chung; 1♂ (TARI), same locality, 28.III.2015, leg. W.-C. Liao; 1♂ (TARI), Tona (多納), 24.III.2009, leg. U. Ong; 1♀ (TARI), same but with “20.III.2010”; 1♀ (TARI), same but with “5.IV.2010”; 1♂, 1♀ (TARI), same locality, 12.III.2013, leg. B.-X. Guo; Miaoli: 1♀ (TARI), Sanyi (三義), 2.V.2013, leg. B.-X. Guo; 1♀ (TARI), same but with “leg. Y.-T. Chung”; 2♂ (TARI), Suyuehsueh trail (四月雪步道), 16.III.2020, leg. M.-H. Tsou; 2♂ (TARI), Tahu (大湖), 7.IV.2013, leg. D. Lu; Nantou: 3♂ (TARI), Aowanta (奧萬大), 7.III.2011, leg. C.-F. Lee; 1♀ (NMNS), Checheng (車埕), 6.V.1998, leg. M.-M. Yang & S.-Y. Yang; 1♂ (NMNS), Fenghuangku (鳳凰谷), 22–23.III.1994, leg. C.-S. Lin & W.-T. Yang; 1♀ (NMNS), Huisun Experimental Forest Station (惠蓀林場), 11.VII.1992, leg. W.-T. Yang; 1♂ (TARI), same locality, 20.III.2014, leg. F.-S. Huang; 1♀ (TARI), same locality, 26.IV.2014, leg. B.-X. Guo; 1♂ (TARI), Jihyuehtan (日月潭), 12.III.2015, leg. C.-S. Lin; 1♂ (TARI), Lienhuachi (蓮華池), 23–26.V.1980, leg. K. S. Lin & B. H. Chen; 1♀ (TARI), same locality, 3.VII.2008, leg. J.-F. Tsai; 2♀ (NMNS), Lushan (廬山), 21.V.2021, leg. Tsai, Lai, Liu; 1♂ (NMNS), Meifeng (梅峰), 19.V.2021, leg. J.-F. Tsai; 1♂ (TARI), Penpuhsi (本部溪), III.1971, leg. T.-C. Maa; 1♀ (TARI), Shalisien trail (沙里仙林道), 9.VI.2013, leg. Y.-T. Wang; 2♀ (KMNH), Hsitou (溪頭), 31.III.1986, leg. K. Sugiyama; 4♂ (TARI), same locality (= Shitou), 18.III.2004, leg. C.-F. Lee; 2♀ (TARI), same but with “22.IV.2004”; 1♂ (TARI), same locality, 17.III.2004, leg. H.-Y. Lee; 1♀ (TARI), Sungkang (松崗), 20.VI.2017, leg. B.-X. Guo; 1♀ (TARI), Tatachia (塔塔加), 25.VI.2016, leg. B.-X. Guo; 1♀ (TARI), Tungpu (東埔), 20–22.VI.1980, leg. C. C. Chen; 1♂ (TARI), same locality, 18.IV. –2.V.1981, leg. T. Lin & C. J. Lee; 1♀ (KMNH), same locality, 22.V.1981, K. Kuzugami; 2♂ (TARI), Tunyuan (屯原), 10.III.2010, leg. Y.-F. Hsu; 1♂ (TARI), same locality, 27.IV.2014, leg. M.-H. Tsou; 1♀ (TARI), Wanfengtsun (萬豐村), 12.IV.2007, leg. W.-T. Liu; 1♀ (TARI), same but with “2.IV.2008”; 1♂ (TARI), Wushe, (霧社), 19–22.IV.1983, leg. K. C. Chou & S. P. Huang; Pingtung: 1♂ (TARI), Chiahsing (佳興), 28.IV.2016, leg. Y.-T. Chung; 1♂, 1♀ (TARI), Chiukulou (舊古樓), 17.IV.2021, leg. T.-Y. Chung; 1♂ (KMNH), Kenting Park (墾丁公園), 15.III.1968, leg. Y. Arita; 1♂, 1♀ (KMNH), same locality, 1.IV.1972, leg. Y. Miyake; 2♂ (NMNS), same locality, 13.V.2004, leg. C.-S. Lin & W.-T. Yang; 1♀ (NMNS), Lanjenchi (欖仁溪), 10.VII.1996, leg. M.-L. Chan; 1♀ (TARI), Lilungshan (里龍山), 15.VIII.2009, leg. M.-H. Tsou; 1♀ (TARI), same locality, 2.III.2012, leg. J.-C. Chen; 1♀ (TARI), same locality, 24.III.2014, leg. Y.-T. Chung; 2♀ (TARI), same but with “26.V.2014”; 1♂ (TARI), same but with “16.III.2015”; 2♀ (TARI), same but with “23.III.2016”; 1♀ (TARI), same locality, 21.VIII.2015, leg. S.-P. Wu; 1♂ (TARI), same locality, 17.IV.2018, leg. C.-F. Lee; 1♀ (TARI), Mutantsun (牡丹村), 14.III.1984, leg. K. C. Chou & C. C. Pan; 1♂ (TARI), Nanjenshan (南仁山), 27.III.–5.IV.2010, leg. M.-L. Jeng; 1♀ (TARI), Neiwen (內文), 20.VII.2016, leg. B.-X. Guo; 4♂ (TARI), Peitawushan (北大武山), 21.III.2011, leg. J.-C. Chen; 1♂, 10♀ (TARI), same locality, 4.IV.2013, leg. Y.-T. Chung; 1♂, 2♀ (TARI), same but with “22.IV.2014”; 1♂, 5♀ (TARI), same but with “8.V.2014”; 1♀ (TARI), same but with “28.V.2014”; 2♂, 1♀ (TARI), same but with “21.III.2015”; 4♀ (TARI) Shantimen (三地門), 28.III.–1.IV.1981, leg. C. C. Chen & C. C. Pang; 1♀ (TARI), Sheting (社頂), 20.IV.2015, leg. Y.-T. Chung; 1♂, 3♀ (TARI), Shuangliu (雙流), 19.VII.2007, leg. M.-H. Tsou; 1♀ (TARI), same locality, 18.V.2009, leg. U. Ong; 1♂ (TARI), same locality, 14.III.2018, leg. Y.-T. Chung; 1♂ (TARI), same but with “12.IV.2018”; 1♂, 2♀ (TARI), Suling (四林), 15.IV.2009, leg. U. Ong; 2♀ (TARI), same but with “19.IV.2009”; 1♀ (TARI), Tahanshan (大漢山), 24.VI.2007, leg. C.-F. Lee; 1♀ (TARI), same but with “20.VII.2007”; 1♀ (TARI), same but with “5.IV.2009”; 4♀ (TARI), same but with “26.III.2013”; 1♂ (TARI), same but with “12.IV.2012”; 1♂ (TARI), same locality, 5.IV.2009, leg. Y.-T. Chung; 1♂ (TARI), same but with “14.III.2013”; 2♂, 1♀ (TARI), same but with “3.IV.2013”; 2♂, 2♀ (TARI), same but with “16.IV.2013”; 1♀ (TARI), same but with “10.V.2013”; 2♀ (TARI), same but with “25.V.2013”; 1♀ (TARI), same but with “21.VII.2013”; 1♀ (TARI), same but with “3.IX.2013”; 1♂ (TARI), same but with “17.III.2014”; 1♂, 1♀ (TARI), same but with “6.IV.2014”; 1♀ (TARI), same but with “30.V.2014”; 1♀ (TARI), same but with “6.VI.2014”; 1♂ (TARI), same but with “2.IV.2015”; 2♀ (TARI), same but with “19.V.2015”; 1♀ (TARI), same but with “6.VI.2015”; 1♀ (TARI), same but with “28.III.2016”; 1♀ (TARI), same but with “18.III.2016”; 4♂, 2♀ (TARI), same but with “8.IV.2016”; 1♂, 3♀ (TARI), same but with “4.IV.2017”; 1♂ (TARI), same but with “22.IV.2017”; 1♀ (TARI), same locality, 8.V.2009, leg. U. Ong; 1♂ (TARI), same locality, 31.III.2012, leg. W.-C. Liao; 2♀ (TARI), Tamei (大梅), 25.II.2016, leg. Y.-T. Chung; 4♂, 3♀ (TARI), same but with “12.III.2016”; 1♀ (TARI), Wutai (霧台), 12.IV.2009, leg. U. Ong; 1♀ (TARI), same but with “9.V.2009”; Taichung: 1♂ (TARI), Anmashan (鞍馬山), 24.IV.2012, leg. C.-F. Lee; 1♂ (TARI), same locality, 1.V.2012, leg. W.-T. Liu; 1♀ (TARI), same locality, 28.V.2016, leg. Y.-L. Lin; 3♀ (TARI), Kukuan (谷關), 20–22.VI.1978, leg. K. S. Lin & K. C. Chou; 1♀ (NMNS), same locality, 11–12.IV.1986, leg. C.-S. Lin; 1♂ (TARI), same locality, 19.III.2014, leg. C.-F. Lee; 1♂ (KMNH), Lishan (梨山), 29.VII.1970, leg. Y. Miyake; 1♀ (TARI), Wuling (武陵), 1.VII.2008, leg. M.-H. Tsou; 1♂ (TARI), same locality, 4.IV.2013, leg. J.-C. Chen; Tainan: 1♀ (TARI), Kantoushan (崁頭山), 20.X.2013, leg. W.-C. Liao; 1♂ (TARI), same locality, 1.IV.2018, leg. B.-X. Guo; 1♀ (TARI), Meiling (梅嶺), 4.VI.2010, leg. U. Ong; 2♂ (TARI), same but with “24.III.2011”; 3♀ (TARI), same locality, 24.IV.2013, leg. B.-X. Guo; 1♂, 1♀ (TARI), same locality, 19.IV.2014, leg. W.-C. Liao; Taipei: 6♂ (TARI), Chutzuhu (竹子湖), 6.III.2008, leg. S.-F. Yu; 1♂ (TARI), Erhkoshan (二格山), 14.III.2014, leg. N.-Y. Tsai; 1♂, 3♀ (TARI), Fushan (福山), 5.IV.2007, leg. S.-F. Yu; 1♀ (TARI), same locality, 27.III.2008, leg. H.-J. Chen; 1♂ (TARI), Hsiaoyukeng (小油坑), 24.V.2008, leg. M.-H. Tsou; 1♀ (TARI), Hsiungkung (熊空), 25.I.2007, leg. S.-F. Yu; 1♂ (TARI), Hushan (虎山), 31.III.2006, leg. H.-T. Cheng; 1♂ (TARI), Kueitzukeng (貴仔坑), 25.III.2007, leg. H.-T. Cheng; 1♀ (TARI), Shihting (石碇), 8.V.2008, leg. H.-J. Chen; 1♀ (KMNH), Wulai (烏來), 24.III.1968, leg. Y. Arita; 1♀ (KMNH), same locality (= Urai), 1.VI.1976, leg. H. Makihara; 2♂, 3♀ (TARI), same locality, 27.III.2007, leg. S.-F. Yu; 4♂, 3♀ (TARI), same but with “13.III.2008”; 2♀ (TARI), same locality, 19.VI.2007, leg. M.-H. Tsou; 1♀ (TARI), same locality, 22.VI.2007, leg. H.-J. Chen; 1♂ (TARI), same locality, 19.III.2008, leg. C.-F. Lee; 1♀ (TARI), same but with “17.III.2010”; 1♀ (TARI), same locality, 8.VI.2008, leg. Y.-L. Lin; 1♀ (TARI), same locality, 4.IV.2011, leg. C.-H. Hsieh; 3♀ (TARI), Yangmingshan (陽明山), 19.IV.2007, leg. C.-F. Lee; 1♀ (TARI), same locality, 29.IV.2007, leg. S.-F. Yu; Taitung: 2♀ (TARI), Chinfeng (金峰), 7.IV.2010, leg. W.-T. Liu; 1♀ (KMNH), Kueitien (歸田), 17.VI.1976, leg. H. Makihara; 1♀ (TARI), Lichia (利嘉), 13.VI.2010, leg. Y.-L. Lin; 1♀ (TARI), same locality, 15.VII.2014, leg. Y.-T. Chung; 1♀ (TARI), same locality, 10.V.2018, leg. B.-X. Guo; 1♂ (TARI), Taimali (太麻里), 20.III.2008, leg. P.-F. Wang; 3♀ (TARI), same locality, 12.V.2009, leg. W.-T. Liu; 1♂ (TARI), Wulu (霧鹿), 29.III.2011, leg. M.-H. Tsou; Taoyuan: 1♀ (TARI), Fuhsing (復興), 6.V.1983, leg. K. C. Chou & C. C. Pan; 1♂ (TARI), Hsiaowulai (小烏來), 2.III.2010, leg. S.-F. Yu; 1♂ (TARI), Hsuanyuan (萱源), 16.III.2008, leg. M.-H. Tsou; 1♀ (TARI), 12.VI.2008, leg. H. Lee; 2♀ (TARI), same locality, 30.III.2010, leg. H.-J. Chen; 1♂, 1♀ (TARI), same locality, 13.V.2010, leg. S.-F. Yu; 1♀ (TARI), Kaoi (高義), 19.VI.2010, leg. H.-J. Chen; 1♂, 1♀ (TARI), Lalashan (拉拉山), 16.III.2008, leg. M.-H. Tsou; 1♂ (TARI), same but with “2.V.2009”; 2♂ (TARI), same locality, 8.III.2009, leg. C.-F. Lee; 1♂ (TARI), same locality, 2.IV.2009, leg. H.-J. Chen; 1♀ (KMNH), Paling (巴陵), 25.IV.1982, leg. N. Ohbayashi; 1♂ (NMNS), Sanming (三民), 28.II.1992, leg. C.-Y. Lee; 1♂ (TARI), same locality, 22.III.2015, leg. M.-H. Tsou; 1♂, 2♀ (TARI), Suleng (四稜), 30.III.2010, leg. H.-J. Chen.
