# Supplementary material for: ﻿Revision of the genus Arthrotus Motschulsky, 1858 (Coleoptera, Chrysomelidae, Galerucinae) of Taiwan, with notes on color polymorphism
Source: Zookeys. 2022 Apr 1;1091:161–208. doi: 10.3897/zookeys.1091.79486 (PMC9005468; doi:10.3897/zookeys.1091.79486)
Supplement: Supplementary material 2 — Arthrotusfulvus Chûjô, 1938 [file zookeys-1091-161-s002.docx]

**Supplementary file 2. *Arthrotus fulvus* Chûjô, 1938**

**Other material.** **Form A (*n =* 140)**: Hsichu: 6♀ (TARI), Kuanwu (觀霧), 30.IV.2010, leg. M.-H. Tsou; 2♀ (TARI), same but with “leg. C.-F. Lee”; 5♂, 2♀ (TARI), same locality, 1.V.2010, leg. M.-H. Tsou; 1♀ (TARI), same locality, 6.IV.2010, leg. L.-H. Sun; 1♀ (TARI), same but with “7.VI.2010”; Hualien: 1♀ (TARI), Pilu (碧綠), 16.VI.2008, leg. C.-F. Lee; 3♀ (TARI), same locality, 31.V.2011, leg. M.-H. Tsou; Ilan: 1♀ (TARI), Suyuan (思源), 28.IV.2009, leg. M.-H. Tsou; Miaoli: 1♂ (TARI), Peikenghsi trail (北坑溪古道), 4–5.IV.2018, leg. K. Masumoto; Nantou: 5♀ (TARI), Chingching (清境), 4.IV.2010, leg. Y.-T. Wang; 3♂, 3♀ (TARI), same locality, 9.IX.2018, leg. W.-C. Liao; 1♂ (NMNS), Fenghuangku (鳳凰谷), 15.IV.1994, leg. M.-L. Chan & W.-T. Yang; 1♀ (TARI), Meifeng (梅峯), 10.V.1979, leg. K. C. Chou; 2♀ (TARI), same locality, 20–22.VI.1979, leg. K. S. Lin & B. H. Chen; 1♀ (TARI), same locality, 2–4.VI.1980, leg. L. Y. Chou & C. C. Chen; 2♀ (TARI), same locality, 5–8.VI.1980, leg. C. C. Chen; 1♀ (TARI), same locality, 8.VI.1980, leg. K. S. Lin & B. H. Chen; 1♂, 1♀ (TARI), same locality, 26.VIII.1980, leg. K. S. Lin & C. H. Wang; 1♂, 1♀ (TARI), same locality, 5–9.X.1980, leg. C. C. Chen & C. C. Chien; 2♂, 8♀ (TARI), same locality, 7–9.V.1981, leg. K. S. Lin & S. C. Lin; 2♀ (TARI), same locality, 24–26.VI.1981, leg. W. S. Tang; 2♀ (TARI), same locality, 22.V.1982, leg. L. Y. Chou; 2♀ (TARI), same locality, 4-7.X.1982, leg. K. C. Chou; 2♀ (TARI), same locality, 19–21.IV.1983, leg. K. C. Chou & S. P. Huang; 1♀ (TARI), same locality, 30.VII.1983, leg. L. Y. Chou; 2♀ (TARI), 8–11.V.1984, leg. K. C. Chou & C. C. Pan; 1♂ (NMNS), same locality, 15–16.IV.1996, leg. C.-S. Lin & W.-T. Yang; 1♂ (NMNS), same but with “12.III.–9.IV.2002”; 1♂ (NMNS), same but with “15.X.–12.XI.2002”; 1♂ (NMNS), same but with “6.IX.–4.X.2005”; 3♂, 4♀ (TARI), same locality, 15.IX.2009, leg. S.-F. Yu; 3♂, 3♀ (TARI), same but with “leg. H. Lee”; 1♂ (NMNS), same locality, 21.V.2017, leg. J.-F. Tsai; 2♂, 2♀ (NMNS), same but with “9–10.IV.2018”; 1♂ (NMNS), same but with “1–2.X.2020”; 2♀ (NMNS), same but with “19.V.2021”; 1♀ (NMNS), same locality, 21.X.2020, leg. J.-F. Tsai & B.-C. Lai; 1♂, 1♀ (TARI), Peitungyanshan (北東眼山), 16.IX.2013, leg. F.-S. Huang; 1♂ (TARI), same but with “28.III.2014”; 1♀ (NMNS), Piluhsi (碧綠溪), 6.V.1997, leg. M.-M. Yang; 1♂ (TARI), same locality, 8.VII.2015, leg. C.-F. Lee; 1♀ (TARI), same locality, 21.V.2016, leg. Y.-L. Lin; 7♂, 9♀ (TARI), Sungkang (松崗), 13–15.IX.1984, leg. K. S. Lin & S. C. Lin; 3♀ (TARI), same locality, 4.IV.2010, leg. Y.-T. Wang; 1♂ (TARI), same locality, 18.IV.2015, leg. B.-X. Guo; 2♀ (TARI), same locality, 10.IV.2016, leg. Y.-T. Chung; 2♂, 4♀ (TARI), Tsuifeng (翠峯), 8.V.1981, leg. K. S. Lin & S. C. Lin; 3♀ (TARI), same locality, 25–27.VI.1981, leg. K. S. Lin & W. S. Tang; 1♀ (TARI), same locality, 23.V.1982, leg. L. Y. Chou; 1♂, 1♀ (TARI), same locality, 5.VI.2010, leg. Y.-T. Wang; Taichung: 1♂ (TARI), Anmashan (鞍馬山), 22.IX.2007, leg. M.-H. Tsou; 1♂ (TARI), same locality, 15.X.2009, leg. J.-C. Chen; 1♀ (TARI), same locality, 19.X.2011, leg. C.-F. Lee; 1♀ (TARI), same locality (= Tahsuehshan 大雪山), 24.IV.2012, leg. C.-F. Lee; 1♂ (TARI), Henglingshan (橫嶺山), 5.VI.2012, leg. J.-C. Chen; 2♂ (NMNS), Tachien (達見 = 德基水庫), 2.IX.1987, leg. I.-C. Hsu; **Form B (*n =* 35)**: Chiayi: 1♂, 2♀ (TARI), Alishan (阿里山), 5–9.VIII.1981, leg. L. Y. Chou & S. C. Lin; 1♂ (TARI), same locality, 28.II.2010, leg. U. Ong; 2♀ (TARI), same but with “28.IV.2011”; 3♀ (TARI), same locality, 22.IX.2011, leg. C.-F. Lee; 2♂ (TARI), Tzuchung (自忠), 21.IX.2009, leg. C.-F. Lee; 2♂ (TARI), same but with “leg. M.-H. Tsou”; 2♂, 2♀ (TARI), same locality, 9.IX.2015, leg. C.-F. Lee; 1♀ (TARI), Tzuchungshan (自忠山), 7.VI.2013, leg. Y.-T. Wang; Kaohsiung: 1♀ (TARI), Chungchihkuan (中之關), 25.IX.2015, leg. C.-F. Lee; 2♂, 1♀ (TARI), Tienchih (天池), 1.IV.2015, leg. C.-F. Lee; Nantou: 1♂ (TARI), Tatachia (塔塔加), 25.X.2007, leg. L.-C. Hung; 1♂, 1♀ (TARI), same locality, 5.X.2008, leg. M.-H. Tsou; 1♂ (TARI), same locality, 30.X.2009, leg. C.-F. Lee; 1♂, 1♀ (TARI), same locality, 29.XII.2009, leg. M.-H. Tsou; 1♂, 1♀ (TARI), same locality, 27.IV.2010, leg. C.-F. Lee; 1♂, 2♀ (TARI), same locality, 13.V.2015, leg. C.-F. Lee; 1♀ (TARI), same locality, 17.V.2016, leg. B.-X. Guo; 1♂ (TARI), Tungfu (同富), 8.V.2011, leg. C.-F. Lee; **Form C (*n =* 10)**: Taitung: 1♀ (TARI), Hsiangyang (向陽), 28.III.2014, leg. W.-C. Huang; 1♂ (TARI), Liyuan (栗園), 4.X.2010, leg. M.-H. Tsou; 1♀ (TARI), same locality, 9.VI.2011, leg. U. Ong; 2♀ (TARI), same locality, 24.VII.2013, leg. C.-F. Lee; 1♀ (TARI), same locality, 28.III.2014.leg. J.-C. Chen; 1♀ (TARI), same locality, 19.IV.2014, leg. W.-C. Huang; 1♂, 1♀ (TARI), Motien (摩天), 5.X.2010, leg. C.-F. Lee; 1♀ (TARI), same but with “23.V.2011”;

**Yellowish or reddish brown elytra (*n =* 333):** Chiayi: 1♀ (TARI), Fenchihu (奮起湖), 25.V.2013, leg. W.-C. Liao; 1♂ (TARI), Laichitashan (來吉塔山), 19.III.2009, leg. H. Lee; 1♀ (TARI), Shihpanku (石磐谷), 15.V.2017, leg. B.-X. Guo; Hsichu: 1♀ (TARI), Hsuehchien (雪見), 10.IX.2013, leg. W.-B. Yeh; 1♀ (TARI), Litungshan (李棟山), 23.III.2007, leg. M.-H. Tsou; 1♀ (TARI), same but with “15.III.2009”; 1♂ (TARI), same but with “leg. S.-F. Yu”; 1♂ (TARI), Lupi (魯壁), 26.VII.2008, leg. M.-H. Tsou; 1♀ (TARI), Mamei (馬美), 18.V.2008, leg. M.-H. Tsou; 1♀ (TARI), Taikang (泰崗), 12.IX.2015, leg. Y.-L. Lin; Hualien: 1♀ (TARI), Kuanyuan (關原), 7.V.2006, leg. Y.-F. Hsu; 3♀ (TARI), Pilu (碧綠), 31.V.2011, leg. M.-H. Tsou; 1♀ (NMNS), same locality, 29.IV.–1.VI.2011, leg. W.-T. Yang & K.-W. Huang; 1♂, 1♀ (NMNS), same but with “7.II.–17.IV.2012”; 1♀ (NMNS), same but with “17.IV.–28.V.2012”; 2♀ (TARI), same locality, 13.VI.2014, leg. C.-F. Lee; 1♂ (TARI), same but with “23.IV.2015”; 1♀ (TARI), same locality, 11.VI.2017, leg. B.-X. Guo; Ilan: 1♀ (TARI), Suchi (四季), 1,VIII.2009, leg. M.-H. Tsou; 2♂, 2♀ (TARI), Suyuan (思源), 25.IV.2009, leg. C.-F. Lee; 1♂, 4♀ (TARI), same but with “leg. M.-H. Tsou”; 6♂, 10♀ (TARI), same locality, 28.IV.2009, leg. M.-H. Tsou; 4♂ (TARI), same locality, 22.VII.2009, leg. Y.-L. Lin; 3♂ (TARI), same but with “29.VIII.2009”; 1♂, 2♀ (TARI), same locality, 6.V.2011, leg. S.-F. Yu; 4♂, 2♀ (TARI), same locality, 11.VIII.2014, leg. J.-C. Chen; 2♀ (TARI), same but with “leg. H. Lee”; 1♂ (TARI), Taipingshan (太平山), 22.IV.2007, leg. S.-S. Li; 2♀ (TARI), same locality, 24.IV.2007, leg. W.-T. Liu; 1♂, 1♀ (TARI), same but with “15.VIII.2007”; 1♀ (TARI), same locality, 22.VI.2009, leg. H. Lee; 1♀ (TARI), same locality (= Tsuifenghu, 翠峰湖), 4.VII.2010, leg. M.-H. Tsou; 1♀ (TARI), same locality, 21.V.2016, leg. C.-F. Lee; 1♂ (TARI), Yuanyanghu (鴛鴦湖), 19.VIII.2010, leg. M.-H. Tsou; 1♂, 1♀ (TARI), same locality, 23.VIII.2011, leg. M.-H. Tsou; 1♂, 1♀ (TARI), same but with “leg. H. Lee”; 8♂, 5♀ (TARI), same locality, 22.VIII.2011, leg. C.-F. Lee; 3♂, 1♀ (TARI), same locality, 20.VIII.2016, leg. J.-C. Chen; Kaohsiung: 1♀ (TARI), Chungchihkuan (中之關), 1.VII.2009, leg. S.-F. Yu; 2♂ (TARI), same locality, 14.IV.2012, leg. L.-P. Hsu; 1♂ (TARI), Erhchituan (二集團), 19.III.2013, leg. Y.-T. Chung; 2♂ (KMNH), Liukui (六龜), 3.IV.1986, leg. K. Baba; 1♂ (KMNH), Shaping (扇平), 10.IV.1986, leg. K. Baba; 1♀ (KMNH), Shinanshan (溪南山), 4.V.1986, leg. K. Baba; 1♀ (TARI), Takueihu (大鬼湖), 20.IV.2018, leg. W.-B. Yeh; 1♂ (NMNS), Tengchih (藤枝), 7.IX.1989, leg. K.-W. Huang; 4♀ (TARI), same locality, 2-5.VI.2008, leg. C.-F. Lee; 2♂, 1♀ (TARI), same but with “24.III.2009”; 1♀ (TARI), same but with “leg. H. Lee”; 1♀ (TARI), same but with “leg. M.-H. Tsou”; 1♀ (KMNH), same locality (= Shyk Shan, 石山), 4.V.1986, leg. K. Baba; 1♂ (TARI), same locality (= Shihshan trail, 石山林道), 18.VIII.2008, leg. C.-T. Yao; 1♂, 3♀ (TARI), same locality, 1-3.X.2008, leg. M.-H. Tsou; 1♀ (TARI), same locality, 26.V.2009, leg. S.-F. Yu; 3♂, 1♀ (TARI), same locality, 20.III.2013, leg. B.-X. Guo; 1♂ (TARI), same locality, 18.IV.2013, leg. Y.-T. Chung; 1♂ (TARI), same locality, 10.IX.2013, leg. J.-C. Chen; 8♂ (TARI), same locality, 9.XI.2013, leg. W.-C. Liao; 1♂, 4♀ (TARI), same but with “30.XI.2013”; 1♂, 2♀ (TARI), same but with “28.III.2015”; 1♀ (TARI), same but with “17.X.2015”; 1♂, 1♀ (TARI), same but with “2.IV.2016”; 1♀ (TARI), Tona trail (多納林道), 20.III.2010, leg. U. Ong; 2♀ (TARI), same locality, 17.IX.2014, leg. B.-X. Guo; 1♂, 1♀ (TARI), same but with “7.VII.2016”; 4♂ (TARI), same but with “2.VIII.2017”; Nantou: 2♂ (TARI), Huakang (華岡), 12.IX.2010, leg. C.-F. Lee; 2♂ (TARI), Meifeng (梅峰), 15.IX.2009, leg. S.-F. Yu; 1♀ (TARI), same but with “leg. H. Lee”; 1♂ (KMNH), Nanshanchi (南山溪), 3.IV.1981, leg. Y. Yamamoto; 1♀ (TARI), Nengkaoshan (能高山), 18.X.2011, leg. J.-C. Chen; 2♀ (TARI), Peitungyanshan (北東眼山), 14.V.2015, leg. C.-F. Lee; 1♂ (KMNH), Penpuchi (本部溪), 9.VII.1966, leg. H. Kamiya (identified as *A. testaceus* by Kimoto (1969)); 1♂ (TARI), Shanlinhsi (杉林溪), 9.IX.2019, leg. B.-X. Guo; 1♂ (KMNH), Sungkang (松崗), 21.VIII.1969, leg. Y. Maeda; 1♂ (TARI), same locality, 6.IV.2010, leg. Y.-T. Wang; 2♀ (TARI), Tatachia (塔塔加), 17.V.2010, leg. M.-H. Tsou; 1♀ (TARI), same locality, 9.V.2011, leg. C.-F. Lee; 1♀ (TARI), Tsuifeng (翠峰), 10.IX.2018, leg. W.-C. Liao; 1♂ (TARI), Wanfengtsun (萬豐村), 10.VII.2007, leg. W.-T. Liu; 1♀ (TARI), same but with “13.IV.2010”; 1♀ (TARI), same but with “20.IV.2011”; Pingtung: 2♂ (TARI), Haikou (海口), 7.V.2014, leg. Y.-T. Chung; 2♂ (TARI), Peitawushan (北大武山), 21.III.2011, leg. J.-C. Chen; 2♂ (TARI), same locality, 12.IV.2013, leg. Y.-T. Chung; 1♂ (TARI), same but with “11.VIII.2013”; 2♂ (TARI), same but with “27.VIII.2013”; 1♂ (TARI), same but with “28.X.2017”; 2♀ (TARI), same locality, 21.VII.2013, leg. W.-C. Liao; 1♀ (TARI), same locality, 22.IV.2015, leg. J.-C. Chen; 1♂ (TARI), Shahsi (沙溪), 20.VII.2017, leg. B.-X. Guo; 1♀ (TARI), Tahanshan (大漢山), 18.VII.2007, leg. S.-F. Yu; 2♀ (TARI), same locality, 4.VII.2008, leg. M.-H. Tsou; 2♀ (TARI), same locality, 10.IV.2009, leg. U. Ong; 1♀ (TARI), same but with “19.V.2009”; 1♂, 1♀ (TARI), same but with “1.VIII.2009”; 1♀ (TARI), same but with “8.VI.2010”; 1♂, 1♀ (TARI), same but with “23.III.2012”; 1♂ (TARI), same locality, 2.IX.2009, leg. J.-C. Chen; 1♂ (TARI), same but with “7.IX.2009”; 1♂ (TARI), same but with “23.IX.2009”; 2♀ (TARI), same but with “19.XI.2009”; 3♂ (TARI), same but with “24.II.2010”; 1♀ (TARI), same but with “12.VIII.2010”; 1♂ (TARI), same locality, 13.VIII.2011, leg. Y.-T. Wang; 7♂, 4♀ (TARI), same same but with “14.VIII.2011”; 1♂ (TARI), same locality, 3.VI.2012, leg. W.-C. Liao; 1♂, 3♀ (TARI), same but with “6.IV.2013”; 1♀ (TARI), same but with “27.IV.2014”; 1♂ (TARI), same but with “22.III.2015”; 1♂ (TARI), same but with “28.II.2016”; 1♀, same locality, 25.V.2008, leg. C.-F. Lee; 1♂, 4♀ (TARI), same (= 浸水營, Chinshuiying) but with “12.IV.2012”; 10♂, 3♀ (TARI), same but with “19.VII.2012”; 1♂, 3♀ (TARI), same but with “26.III.2013”; 1♀ (TARI), same locality, 5.IV.2009, leg. Y.-T. Chung; 1♀ (TARI), same but with “7.VI.2009”; 1♀ (TARI), same but with “28.VI.2009”; 1♂ (TARI), same but with “22.VI.2012”; 1♂ (TARI), same but with “13.VIII.2012”; 3♂ (TARI), same but with “26.II.2013”; 2♂ (TARI), same but with “7.III.2013”; 1♀ (TARI), same but with “16.IV.2013”; 1♂ (TARI), same but with “10.VII.2013”; 2♂ (TARI), same but with “21.VII.2013”; 1♂, 5♀ (TARI), same but with “30.VII.2013”; 1♀ (TARI), same but with “11.X.2013”; 1♂ (TARI), same but with “17.III.2014”; 1♀ (TARI), same but with “25.III.2015”; 1♂ (TARI), same but with “19.X.2015”; 2♂ (TARI), same but with “18.III.2016”; 1♂, 1♀ (TARI), same but with “28.III.2016”; 1♂, 2♀ (TARI), same but with “8.IV.2016”; 1♂ (TARI), same but with “28.VII.2016”; 1♂, 1♀ (TARI), same but with “4.IV.2017”; 1♂ (TARI), same but with “15.IV.2018”; 1♂ (TARI), same but with “23.IX.2019”; 1♂ (TARI), same but with “10.IV.2020”; 1♂ (TARI), same but with “14.IV.2020”; 1♂ (TARI), same but with “25.III.2021”; 2♀ (TARI), same locality, 25.III.2013, leg. B.-X. Guo; 1♀ (TARI), same locality, 4-6.IV.2015, leg. K. Masumoto; 1♀ (TARI), Wutai (霧台), 15-16.III.2009, leg. Y.-F. Hsu; 1♀ (TARI), same locality, 12.IV.2009, leg. U. Ong; 2♂ (TARI), same but with “27.III.2010”; 1♀ (TARI), same locality, 23.VI.2014, leg. J.-C. Chen; Taichung: 1♀ (NMNS), Anmashan (鞍馬山), 13.VII.2019, leg. J.-F. Tsai; 1♂ (NMNS), same but with “27.VIII.2020”; 1♂ (TARI), same locality, 10.X.2018, leg. H.-C. Liu; 1♀ (TARI), Pilu (畢祿), 18.VI.2010, leg. C.-F. Lee; Tainan: 1♀ (TARI), Meiling (梅嶺), 12.III.2011, leg. M.-L. Jeng; 4♀ (TARI), same locality, 19.IV.2014, leg. W.-C. Liao; 1♀ (TARI), Pichien trail (碧尖林道), 22.III.2010, leg. U. Ong; Taitung: 1♀ (TARI), Lichia (利嘉), 19.V.2009, leg. U. Ong; 1♀ (TARI), same locality, 16.VII.2014, leg. Y.-T. Wang; 1♀ (TARI), Litao (利稻), 23.VI.2010, leg. M.-H. Tsou; 1♂ (TARI), Liyuan (栗園), 4.X.2010, leg. M.-H. Tsou; 4♂, 1♀ (TARI), same locality, 24.VII.2013, leg. C.-F. Lee; 1♂ (TARI), same locality, 17.IX.2020, leg. Y.-T. Chung; 2♂, 1♀ (TARI), Motien (摩天), 5.X.2010, leg. C.-F. Lee; 1♀ (TARI), same but with “23.V.2011”; 1♀ (TARI), Taimali (太麻里), 20.III.2008, leg. P.-F. Wang; 1♂ (TARI), Wulu (霧鹿), 23.VI.2010, leg. M.-H. Tsou; 1♂ (TARI), same but with “29.III.2011”; Taoyuan: 1♂ (TARI), Lalashan (拉拉山), 7.VIII.2008, leg. H.-J. Chen; 1♀ (TARI), same but with “8.III.2009”; 1♂ (TARI), same locality, 26.X.2008, leg. M.-H. Tsou; 1♀ (TARI), same locality, 30.X.2008, leg. S.-F. Yu; 3♀ (TARI), same but with “4.V.2010”; 1♂ (TARI), same locality, 23.VII.2009, leg. H. Lee; Yunlin: 1♂ (TARI), Chiananyunfeng (嘉南雲峰), 29.IX.2013, leg. W.-C. Liao; 1♀ (TARI), Shihpishan (石壁山), 26.IV.2015, leg. W.-C. Liao.

**Maculate or metallic blue elytra (*n =* 130):** Chiayi: 1♀ (KMNH), Alishan (阿里山), 6.VII.1965, leg. R. Ishikawa; 1♂ (KMNH), same locality, 18.V.1981, leg. N. Ito; 1♂ (TARI), same locality, 22.IX.2011, leg. C.-F. Lee; 1♀ (KMNH), Tapan (達邦), 17.V.1974, leg. S. Takeda; 1♀ (TARI), Tefuyeh (特富野), 15.V.2020, leg. P.-H. Kao; Hsinchu: 1♂, 1♀ (TARI), Chenghsipao (鎮西堡), 26.VII.2014, leg. Y.-L. Lin; 1♀ (TARI), Kuanwu (觀霧), 30.IV.2009, leg. Y.-F. Hsu; 3♀ (TARI), same locality, 30.IV.2010, leg. M.-H. Tsou; 1♀ (TARI), Wufeng (五峰), 26.IX.2009, leg. Y.-L. Lin; Hualien: 4♀ (TARI), Pilu (碧綠), 31.V.2011, leg. M.-H. Tsou; 1♀ (TARI), same locality, 11.VI.2017, leg. B.-X. Guo; 1♀ (TARI), same locality, 27.IV.2018, leg. H.-F. Lu; 1♀ (TARI), Tayuling (大禹嶺), 4.V.2014, leg. J.-F. Tsai; Ilan: 3♀ (TARI), Suyuan (思源), 25.IV.2009, leg. M.-H. Tsou; 1♂ (TARI), same locality, 29.VIII.2009, leg. Y.-L. Lin; 1♀ (TARI), same locality, 6.V.2011, leg. S.-F. Yu; 1♀ (TARI), Taipingshan (太平山), 26.V.2007, leg. S.-S. Li; 2♀ (TARI), same locality (= Tsuifenghu, 翠峰湖), 4.VII.2010, leg. M.-H. Tsou; 2♀ (TARI), same locality, 10.VI.2011, leg. S.-F. Yu; 1♂ (TARI), same locality, 5.VIII.2015, leg. Y.-T. Chung; 1♀ (TARI), same locality, 21.V.2016, leg. C.-F. Lee; 1♀ (TARI), same locality (= Chienching trail, 見晴步道), 23.IV.2019, leg. M.-D. Chen; 1♀ (TARI), same locality, 1.VIII.2016, leg. S.-P. Wu; 1♀ (TARI), Tungshan (銅山), 31.III.2010, leg. Y.-F. Hsu; 6♂, 1♀ (TARI), Yuanyanghu (鴛鴦湖), 22.VIII.2011, leg. C.-F. Lee; 1♂ (TARI), same locality, 23.VIII.2011, leg. M.-H. Tsou; Kaohsiung: 1♂, 1♀ (TARI), Chungchihkuan (中之關), 15.IX.–10.X.2012, leg. L.-P. Hsu; 1♂ (TARI), same locality, 20.IV.2016, leg. C.-F. Lee; 1♂ (KMNH), Thuyunshan (出雲山), 23.VII.1986, leg. K. Baba; 1♀ (TARI), Tona trail (多納林道), 7.VII.2016, leg. B.-X. Guo; Nantou: 1♂, 1♀ (TARI), Fenghuangshan (鳳凰山), 10.VIII.2011, leg. M.-H. Tsou; 1♂ (TARI), same locality (= Hsitou, 溪頭), 11.XI.2013, leg. H.-T. Yeh; 1♂ (HTC), Habonsan (合望山 = 北東眼山), 7.IX.1983, leg. K. Ra; 1♂ (HTC), same locality, 2.VIII.1985, leg. H. Takizawa; 1♀ (TARI), Huakang (華岡), 12.IX.2010, leg. C.-F. Lee; 1♂, 2♀ (TARI), Meifeng (梅峰), 15.IX.2009, leg. S.-F. Yu; 2♂ (TARI), same but with “leg. H. Lee”; 1♀ (NMNS), same locality, 28.VII.-25.IX.1997, leg. C.-S. Lin & W.-T. Yang; 1♀ (NMNS), same but with “15.IV.–7.V.2003”; 1♂ (NMNS), same but with “2.VIII.-8.IX.2005”; 1♂ (NMNS), same locality, 1–3.XI.2017, leg. J.-F. Tsai; 1♀ (NMNS), same but with “28.VIII.2020”; 1♀ (NMNS), same but with “1–2.X.2020”; 1♀ (NMNS), same but with “19.V.2021”; 1♀ (TARI), Sungkang (松崗), 4.IV.1971, leg. H. Nomura; 2♂ (HTC), same locality, 8.VIII.1983, leg. K. Ra; 1♂ (HTC), same locality, 1.VIII.1985, leg. H. Takizawa; 1♀ (TARI), Tatachia (塔塔加), 27.IV.2010, leg. C.-F. Lee; 2♀ (TARI), same but with “17.V.2010”; 1♀ (TARI), same but with “13.V.2015”; 1♀ (TARI), same locality, 9.V.2011, leg. M.-H. Tsou; 1♀ (TARI), same locality, 17.V.2016, leg. B.-X. Guo; 1♂, 1♀ (HTC), Tongpo (= Tungpu, 東埔), 16-18.VII.1995, leg. H. Takizawa; 2♂, 1♀ (TARI), Tsuifeng (翠峰), 9.IV.2014, leg. C.-F. Lee; 1♂ (TARI), same but with “21.IV.2015”; Pingtung: 1♀ (TARI), Peitawushan (北大武山), 23.VI.2011, leg. J.-C. Chen; 1♀ (TARI), same but with “11.IX.2015”; 1♀ (TARI), Tahanshan (大漢山), 14.VIII.2011, leg. Y.-T. Wang; 1♂ (TARI), same locality, 1.VIII.2020, leg. Y.-T. Chung; 1♀ (TARI), same but with “12.IV.2021”; 1♀ (TARI), same but with “18.IV.2021”; 4♀ (TARI), same but with “23.IV.2021”; Taichung: 1♀ (TARI), Anmashan (鞍馬山), 7.VI.2010, leg. C.-F. Lee; 1♀ (NMNS), Pahsienshan (八仙山), 23.VII.1991, leg. C.-C. Chiang; 1♂ (NMNS), Tachien (達見 = 德基水庫), 2.IX.1987, leg. I.-C. Hsu; Taitung: 1♀ (TARI), Hsiangyang (向陽), 8.VII.2010, leg. J.-C. Chen; 7♂ (TARI), same but with “5.IV.2012”; 1♂, 2♀ (TARI), same but with “9.V.2013”; 1♀ (TARI), same but with “19.IV.2014”; 1♀ (TARI), same but with “17.V.2014”; 2♀ (TARI), same locality, 14.VIII.2012, leg. C.-F. Lee; 1♀ (TARI), same but with “18.VI.2013”; 1♂, 1♀ (TARI), same locality, 28.III.2014, leg. W.-C. Huang; 3♀ (TARI), Liyuan (栗園), 29.VI.2013, leg. C.-F. Lee; 5♂, 3♀ (TARI), Motien (摩天), 5.X.2010, leg. C.-F. Lee; 1♂, 7♀ (TARI), same but with “23.V.2011”; 2♀ (TARI), same but with “19.VI.2011”; 1♀ (TARI), Wulu (霧鹿), 30.IV.2010, leg. M.-H. Tsou; 1♀ (TARI), same but with “24.VI.2010”; 1♂ (TARI), same but with “5.X.2010,”; Taoyuan: 1♀ (TARI), Lalashan (拉拉山), 4.V.2010, leg. S.-F. Yu; 1♀ (TARI), same locality, 9.VI.2012, leg. M.-H. Tsou; 1♂ (TARI), Paling (巴陵), 2.IX.2009, leg. H. Lee.
