# Supplementary material for: ﻿Revision of the genus Arthrotus Motschulsky, 1858 (Coleoptera, Chrysomelidae, Galerucinae) of Taiwan, with notes on color polymorphism
Source: Zookeys. 2022 Apr 1;1091:161–208. doi: 10.3897/zookeys.1091.79486 (PMC9005468; doi:10.3897/zookeys.1091.79486)
Supplement: Supplementary material 3 — Arthrotustricolor (Chûjô, 1965) [file zookeys-1091-161-s003.docx]

**Supplementary file 3. *Arthrotus tricolor* (Chûjô, 1965)**

**Other material (*n =* 299).** TAIWAN. Chiayi: 1♀ (TARI), Fenchihu (奮起湖), 18.V.2014, leg. W.-C. Liao; 2♀ (TARI), Mihu trail (迷糊步道), 28.IV.2013, leg. W.-C. Liao; Hsinchu: 1♀ (TARI), Chienshih (尖石), 12.IV.2014, leg. H.-Y. Ku; 1♂ (TARI), Chingchuan (清泉), 15.VIII.2015, leg. Y.-L. Lin; 1♀ (TARI), Litungshan (李棟山), 23.III.2007, leg. M.-H. Tsou; 1♂ (TARI), same but with “15.III.2009”; 1♀ (TARI), Lupi (魯壁), 20.VII.2008, leg. M.-H. Tsou; 1♂, 4♀ (TARI), same but with “26.VII.2008”; 1♂ (TARI), same locality, 10.III.2009, leg. S.-F. Yu; 1♂ (TARI), same locality, 12.III.2009, leg. H. Lee; 1♂ (TARI), same but with “leg. S.-F. Yu”; 1♂, 2♀ (TARI), Mamei (馬美), 4.V.2008, leg. S.-F. Yu; 2♀ (TARI), same locality, 18.V.2008, leg. M.-H. Tsou; 1♂, 1♀ (TARI), Tahunshan (大混山), 8.IX.2009, leg. S.-F. Yu; 1♀ (TARI), Talu trail (大鹿林道), 17.V.2008, leg. Y.-L. Lin; 1♀ (TARI), same but with “22.VIII.2009”; 1♀ (TARI), Wufeng (五峰), 17.III.2009, leg. S.-F. Yu; 1♀ (TARI), Yulao (宇老), 6.IV.2015, leg. C.-C. Chen; Hualien: 1♂ (NMNS), Hualuhsi (華綠溪), 12.I.–26.III.2009, leg. W.-T. Yang & K.-W. Huang; 3♂ (NMNS), same but with “24.VIII.-24.IX.2009”; Ilan: 2♀ (TARI), Fushan Botanical Park (福山植物園), 1–2.IV.2008, leg. M.-H. Tsou; 1♀ (TARI), same but with “14.II.2009”; 1♂ (TARI), same locality, 13.IV.2011, leg. C.-F. Lee; 1♂ (TARI), same locality, 30.VI.2013, leg. Y.-T. Wang; 2♂, 7♀ (TARI), same but with “3-9.VII.2013”; 1♀ (TARI), Mingchi (明池), 29.VII.2007, leg. M.-H. Tsou; 3♀ (TARI), same but with “27.IV.2008”; 1♂, 2♀ (TARI), same but with “25.V.2008”; 2♂, 5♀ (TARI), same but with “16.VIII.2008”; 1♀ (TARI), Nanshan (南山), 9.IV.2011, leg. M.-H. Tsou; Kaohsiung: 1♀ (KMNH), Liukuei (六龜), 3.IV.1986, leg. K. Baba; 2♀ (KMNH), Tsai Tei Ku (?), near Liukui (六龜), 4.VI.1989, leg. K. Baba; 1♂, 1♀ (TARI), Tengchih (藤枝), 21.VIII.2012, leg. J.-C. Chen; 1♀ (TARI), same locality, 18.IV.2013, leg. B.-X. Guo; 1♀ (TARI), same but with “11.VIII.2013”; 1♂, 2♀ (TARI), same locality, 10.VIII.2013, leg. W.-C. Liao; 2♀ (TARI), same but with “8.II.2014”; 1♀ (TARI), same but with “28.III.2015”; 1♀ (TARI), same locality, 5.VIII.2018, leg. Y.-T. Chung; 1♂, 1♀ (TARI), same but with “3.IV.2020”; Nantou: 3♀ (NMNS), Aowanta (奧萬大), 22-23.VIII.2018, leg. J.-F. Tsai; 1♂ (NMNS), Chungyang (春陽), 11.VI.–9.VII.2002, leg. C.-S. Lin & W.-T. Yang; 1♂, 1♀ (NMNS), same but with “9.VII.–13.VIII.2002”; 2♂, 1♀ (NMNS), same but with “8.VII.–5.VIII.2003”; 1♀ (NMNS), same but with “5.VIII.–9.IX.2003”; 1♀ (NMNS), same but with “7.X.–4.XI.2003”; 3♂, 2♀ (NMNS), same but with “4.XI.–15.XII.2003”; 1♀ (NMNS), same but with “6.IV.–11.V.2004”; 1♂ (NMNS), same but with “13.VII.–10.VIII.2004”; 1♀ (NMNS), same but with “25.VII.–22.VIII.2006”; 1♀ (NMNS), same but with “17.X.–14.XI.2006”; 1♂ (NMNS), same but with “14.XI.–12.XII.2006”; 1♀ (NMNS), same but with “12.IV.–8.V.2007”; 1♀ (NMNS), same but with “7.VIII.–4.IX.2007”; 1♀ (TARI), Fenghuangshan (鳳凰山), 10.VIII.2011, leg. M.-H. Tsou; 1♀ (NMNS), Huisun Experimental Forest Station (惠蓀林場), 11.VII.1992, leg. W.-T. Yang; 1♂ (TARI), same locality, 26.VIII.2013, leg. F.-S. Huang; 1♂ (TARI), same but with “6.V.2014”; 1♂, 2♀ (TARI), same locality, 23.IV.2015, leg. Y.-T. Chung; 1♀ (NMNS), Lienhuachih (蓮華池), 4.III.–6.V.2003, leg. C.-S. Lin & W.-T. Yang; 1♂ (NMNS), same but with “6.X.–3.XI.2003”; 1♀ (NMNS), same but with “4.X.–15.XI.2004”; 1♂ (NMNS), Lixing Industrial road (力行產業道路) 4–5Km, 4.XI.2020, leg. J.-F. Tsai & W.-T. Yang; 2♂ (NMNS), Lixing Industrial road (力行產業道路) 11–12Km, 16.IX.2020, leg. J.-F. Tsai & B.-C. Lai; 5♂ (NMNS), Lushan (廬山), 29.VII.2019, leg. J.-F. Tsai; 1♀ (NMNS), same but with “17.IX.2020”; 1♀ (KMNH), Nanshanchi (南山溪), 31.III.1981, leg. Y. Yamamoto; 1♂ (KMNH), same locality, 22.IV.1983, leg. F. Kimura; 1♀ (KMNH), same locality, 7.V.1983, leg. N. Ito; 2♀ (TARI), Tungpu (東埔), 28.IV.-2.V.1981, leg. T. Ling & C. J. Lee; 1♂ (TARI), same locality, 5–8.X.1981, leg. T. Lin & W. S. Tang; 2♂, 1♀ (TARI), same locality, 19–23.VII.1982, leg. L. Y. Chou & T. Lin; 1♀ (TARI), same locality, 22–25.XI.1982, leg. K. C. Chou & S. P. Huang; 2♂, 4♀ (TARI), same locality, 20–24.VI.1983, leg. K. C. Chou & C. Y. Wong; 1♂, 1♀ (TARI), same locality, 16–20.IV.1984, leg. K. C. Chou & C. H. Yung; 12♂, 5♀ (TARI), same locality, 23–27.VII.1984, leg. K. C. Chou & C. H. Yang; 2♂ (TARI), Wushe (霧社), 19–22.IV.1983, leg. K. C. Chou & S. P. Huang; 1♀ (TARI), same locality, 7.V.1984, leg. K. C. Chou & C. C. Pan; 1♀ (TARI), same locality, 17.VIII.1984, leg. K. C. Chou; Pingtung: 1♀ (TARI), Lilungshan (里龍山), 27.III.2012, leg. J.-C. Chen; 2♀ (TARI), same but with “19.III.2013”; 2♂ (TARI), same but with “8.III.2014”; 1♂ (TARI), same but with “10.VI.2014”; 1♂, 1♀ (TARI), same locality, 17.VIII.2014, leg. Y.-T. Chung; 1♂ (TARI), Tahanshan (大漢山), 7.IX.2008, leg. W.-C. Wang; 1♂, 1♀ (TARI), same but with “18.IX.2008”; 1♂ (TARI), same locality, 27.VIII.2009, leg. J.-C. Chen; 1♂ (TARI), same but with “23.IX.2009”; 1♀ (TARI), same locality, 14.VIII.2011, leg. Y.-T. Wang; 1♂ (TARI), same locality (= Chinshuiying, 浸水營), 5.IV.2009, leg. C.-F. Lee; 1♀ (TARI), same but with “12.IV.2012”; 1♀ (TARI), same but with “26.III.2013”; 1♂, 1♀ (TARI), same locality, 3.IV.2013, leg. Y.-T. Chung; 1♀ (TARI), same but with “16.IV.2013”; 1♀ (TARI), same but with “2.VI.2013”; 1♀ (TARI), same but with “9.VI.2013”; 2♂, 1♀ (TARI), same but with “2-3.IX.2013”; 1♀ (TARI), same but with “6.VI.2015”; 1♀ (TARI), same but with “19.V.2015”; 3♂ (TARI), same but with “18.III.2016”; 1♂ (TARI), same but with “28.III.2016”; 1♂ (TARI), same but with “28.VII.2016”; 1♂ (TARI), same but with “4.IV.2017”; 2♂, 1♀ (TARI), same but with “10.IV.2017”; 1♂ (TARI), same but with “30.IV.2018”; 2♂, 1♀ (TARI), same but with “26.VII.2019”; 1♂, 1♀ (TARI), same locality, 19.VII.2014, leg. W.-C. Liao; Taichung: 2♂, 7♀ (TARI), Chiapaotai (佳保台), 14–18.X.1980, leg. K. S. Lin & C. H. Wang; 1♂, 1♀ (TARI), Kukuan (谷關), 14–17.X.1980, leg. K. S. Lin & C. H. Wang; 2♂ (TARI), same locality, 19.III.2014, leg. C.-F. Lee; 3♀ (KMNH), Pahsienshan (八仙山), 29.V.1971, leg. K. Kanmiya; 2♂ (NMNS), Tangmatanshan (唐麻丹山), 14.X.1987, leg. I.-C. Hsu; 1♀ (NMNS), Tachien (達見 = 德基水庫), 2.IX.1987, leg. I.-C. Hsu; 1♀ (TARI), Tahsuehshan (大雪山 = 鞍馬山), 8.V.2021, leg. C.-W. Tsai; 1♂ (TARI), Wushihkeng (烏石坑), 13.VII.2008, leg. M.-H. Tsou; 1♀ (TARI), same but with “leg. C.-F. Lee”; 1♀ (TARI), same locality, 29.VIII.2014, leg. C.-F. Lee; Taipei: 1♀ (TARI), Erhkoshan (二格山), 3.V.2008, leg. M.-H. Tsou; 1♀ (TARI), same but with “leg. S.-F. Yu”; 1♀ (TARI), same locality, 2.XI.2008, leg. M.-H. Tsou; 1♂ (TARI), Erhtzuping (二子坪), 7.VIII.2010, leg. M.-H. Tsou; 1♀ (TARI), Fushan (福山), 20.VI.2008, leg. H.-J. Chen; 2♂, 3♀ (TARI), Menghuanhu (夢幻湖), 9.VII.2007, leg. S.-F. Yu; 5♂, 3♀ (TARI), same locality, 22.VII.2007, leg. M.-H. Tsou; 1♂ (NMNS), Mientienshan (面天山), 2.VIII.1987, leg. I.-C. Hsu; 1♂ (TARI), Tatungshan (大桶山), 3.IV.2008, leg. H. Lee; 1♂ (TARI), Tsaikungkengshan (菜公坑山), 28.VI.2009, leg. S.-F. Yu; 1♀ (TARI), Wulai (烏來), 24.VI.2007, leg. S.-F. Yu; 1♀ (NMNS), Yangmingshan (陽明山), 3.IV.1998, leg. M.-L. Chan; 2♂ (TARI), same locality, 10.III.2007, leg. M.-H. Tsou; 1♀ (TARI), same locality, 19.IV.2007, leg. C.-F. Lee; 1♀ (TARI), same locality, 29.IV.2007, leg. S.-F. Yu; 1♂ (TARI), Yingtzuling (鶯子嶺), 25.VI.2016, leg. Y.-L. Lin; Taitung: 2♂, 1♀ (TARI), Lichia (利嘉), 15.VII.2014, leg. Y.-T. Chung; 2♂, 1♀ (TARI), same but with “leg. B.-X. Guo”; 2♂ (TARI), same but with “leg. C.-C. Chen”; 8♂, 3♀ (TARI), same locality, 16–17.VII.2014, leg. Y.-T. Wang; 1♂ (TARI), same locality, 25.VII.2015, leg. Y.-T. Chung; B.-X. Guo & S.-P. Wu; 3♂, 1♀ (TARI), same locality, 1.VII.2016, leg. B.-X. Guo; 1♂ (TARI), Liyuan (栗園), 29.III.2011, leg. C.-F. Lee; 1♂ (TARI), Tulanshan (都蘭山), 5.VI.2010, leg. J.-C. Chen; 1♂ (TARI), Wulu (霧鹿), 23.VI.2010, leg. M.-H. Tsou; Taoyuan: 1♀ (TARI), Fufushan (夫婦山), 5.IV.2015, leg. M.-H. Tsou; 1♂ (TARI), Hsiaowulai (小烏來), 29.IX.2009, leg. M.-H. Tsou; 1♀ (TARI), Hsuanyuan (萱源), 13.V.2010, leg. S.-F. Yu; 1♂ (TARI), Hsuehwunao (雪霧鬧), 10.IV.2011, leg. M.-H. Tsou; 2♂ (TARI), same locality, 7.III.2015, leg. Y.-L. Lin; 2♂, 1♀ (TARI), Junghua (榮華), 28.II.2021, leg. H.-Y. Ku; 2♂ (KMNH), Palin (巴陵), 14.VIII.1987, leg. K. Baba; 1♂, 1♀ (NMNS), Shangpaling (上巴陵), 26.VIII.1987, leg. I.-C. Hsu; 2♂, 2♀ (TARI), Tamanshan (塔曼山), 3.VIII.2008, leg. M.-H. Tsou; 1♀ (TARI) Tungman (東滿), 27.V.2008, leg. H. Lee; 1♂ (TARI), Tungyanshan (東眼山), 20.IX.2007, leg. S.-F. Yu; 1♂ (TARI), same locality, 10.V.2009, leg. M.-H. Tsou; 2♀ (KMNH), Tzudran (池端), 28.IV.1982, leg. N. Ohbayashi; Yunlin: 1♀ (TARI), Shihpishan (石壁山), 26.IV.2015, leg. W.-C. Liao.
