# Supplementary material for: ﻿Revision of the genus Arthrotus Motschulsky, 1858 (Coleoptera, Chrysomelidae, Galerucinae) of Taiwan, with notes on color polymorphism
Source: Zookeys. 2022 Apr 1;1091:161–208. doi: 10.3897/zookeys.1091.79486 (PMC9005468; doi:10.3897/zookeys.1091.79486)
Supplement: Supplementary material 4 — Arthrotustestaceus Gressitt & Kimoto, 1963 [file zookeys-1091-161-s004.docx]

**Supplementary file 4. *Arthrotus testaceus* Gressitt & Kimoto, 1963**

**Other material (*n =* 301).** CHINA. Zhejiang: 1♂ (TARI), Qingyuan (庆元), Baishanzu (百山祖), 25.IV.1994, leg. H. Wu; TAIWAN. Chiayi: 1♀ (TARI), Mihu trail (迷糊步道), 28.IV.2013, leg. W.-C. Liao; 1♀ (TARI), Tsengwen Reservoir (曾文水庫), 28.VI.2015, leg. U. Ong; 2♂ (TARI), same but with “2.IV.2016”; 1♀ (TARI), same but with “2.VII.2016”; Hsinchu: 1♂ (TARI), Litungshan (李棟山), 15.III.2009, leg. M.-H. Tsou; 1♀ (TARI), Mamei (馬美), 18.V.2008, leg. M.-H. Tsou; 4♂ (TARI), Wufeng (五峰), 17.III.2009, leg. S.-F. Yu; 1♂ (TARI), Tahunshan (大混山), 1.III.2009, leg. S.-F. Yu; 1♀ (TARI), Talu trail (大鹿林道), 31.VII.2010, leg. Y.-L. Lin; 3♂ (TARI), same but with “17.III.2012”; 1♀ (TARI), same but with “3.IV.2015”; 1♀ (TARI), same but with “19.VIII.2017”; Hualien: 1♀ (TARI), Hutoushan (虎頭山), 21.IV.2018, leg. H.-F. Lu; Ilan: 1♀ (NMNS), Chihtuan (池端), 18.IV.1990, leg. C.-C. Chiang; 1♀ (TARI), Chilan (棲蘭), 30.III.2010, leg. H.-J. Chen; 1♂ (TARI), Fushan Botanical Park (福山植物園), 20.III.2009, leg. C.-F. Lee; 1♂ (TARI), same locality, 1.VII.2013, leg. Y.-T. Wang; 2♂, 2♀ (TARI), Mingchi (明池), 16.VIII.2008, leg. M.-H. Tsou; 1♀ (TARI), same but with “27.IV.2008”; 1♀ (TARI), Nanshan (南山), 9.IV.2011, leg. M.-H. Tsou; Kaohsiung: 1♀ (TARI), Chungchihkuan (中之關), 22.IV.2016, leg. C.-F. Lee; 1♂ (KMNH), Chuyunshan (出雲山), 10.V.1986, leg. K. Baba; 1♀ (TARI), Ertchituan (二集團), 1.V.2009, leg. U. Ong; 3♂ (TARI), same locality, 1.IV.2015, leg. B.-X. Buo; 1♂ (KMNH), Liukui (六龜), 3.IV.1986, leg. K. Baba; 1♀ (KMNH), Nanfengshan (南鳳山), 18.IV.1986, leg. K. Baba; 1♀ (KMNH), Shaping (扇平), 10.IV.1986, leg. K. Baba; 1♂ (TARI), Tengchih (藤枝), 4.VIII.2012, leg. J.-C. Chen; 1♂ (TARI), same locality, 7.IX.2012, leg. W.-C. Liao; 1♂ (TARI), same but with “13.IV.2013”; 2♂ (TARI), same but with “28.III.2015”; 1♀ (TARI), same but with “2.IV.2016”; 1♀ (TARI), same locality, 18.IV.2013, leg. B.-X. Guo; 2♂, 1♀ (TARI), same but with “leg. Y.-T. Chung”; 1♀ (TARI), same locality, 1.VIII.2016, leg. B.-X. Guo; 2♀ (TARI), same locality, 8.IV.2020, leg. Y.-T. Chung; Nantou: 2♂, 2♀ (NMNS), Aowanta (奧萬大), 22-23.VIII.2018, leg. J.-F. Tsai; 1♂, 1♀ (TARI), Huakang (華岡), 14.IX.2010, leg. C.-F. Lee; 1♀ (TARI), Huisun Experimental Forest Station (惠蓀林場), 26.IV.2014, leg. B.-X. Guo; 1♂, 2♀ (TARI), same but with “22.IV.2015”; 5♀ (TARI), same locality, 23.IV.2015, leg. Y.-T. Chung; 1♂ (KMNH), Jiuyuehtan (日月潭), 28.VI.1965, leg. T. Shirôzu (identified as *A. testaceus* by Kimoto 1969); 1♀ (KMNH), same locality (= Sun Mooth Lake), 3.VII.1986, leg. K. Baba; 1♀ (TARI), Kuantaoshan (關刀山), 19.IV.2014, leg. Y.-L. Lin; 1♀ (NMNS), Meifeng (梅峰), VI.2019, leg. J.-F. Tsai & S,-R. Wei; 1♂ (KMNH), Nanshanchi (南山溪), 12.VII.1966, leg. H. Kamiya (identified as *A. testaceus* by Kimoto (1969)); 2♂ (HTC), same locality, 7–12.VII.1983, leg. H. Takizawa; 1♀ (HTC), same but with “31.VII.1985”; 1♂, 1♀ (KMNH), Penpuchi (本部溪), 13.VII.1966, leg. H. Kamiya (identified as *A. testaceus* by Kimoto (1969)); 1♀ (TARI), 23.IV.2015, leg. B.-X. Guo; 1♂ (TARI), Wanfengtsun (萬豐村), 20.IV.2011, leg. W.-T. Liu; 3♀ (TARI), Wushe (霧社), 14.IV.2010, leg. W.-T. Liu; Pingtung: 1♂ (TARI), Lilungshan (里龍山), 6.VIII.2018, leg. B.-X. Guo; 1♀ (TARI), Machia (瑪家), 2.VIII.2016, leg. Y.-T. Chung; 2♂, 2♀ (TARI), Peitawushan (北大武山), 11.IV.2010, leg. J.-C. Chen; 1♀ (TARI), same but with “21.VII.2010”; 1♂ (TARI), same but with “21.III.2011”; 1♀ (TARI), same locality, 22.XII.2012, leg. W.-C. Liao; 1♀ (TARI), same locality, 11.VIII.2013, leg. Y.-T. Chung; 1♀ (TARI), same but with “19.VIII.2013”; 1♂ (TARI), same but with “22.IV.2014”; 1♂ (TARI), same but with “24.III.2015”; 3♂, 1♀ (TARI), same but with “1.VIII.2015”; 1♀ (TARI), same but with “28.X.2017”; 1♂ (TARI), Shouka (壽卡), 1.VI.2009, leg. U. Ong; 1♂ (TARI), same locality, 27.II.2015, leg. J.-C. Chen; 1♀ (TARI), Tahanshan (大漢山), 25.V.2008, leg. C.-F. Lee; 2♂, 1♀ (TARI), same but with “5.IV.2009”; 6♀ (TARI), same but with “12.IV.2012”; 1♀ (TARI), same but with “6.VI.2012”; 1♂, 3♀ (TARI), same but with “19.VII.2012”; 3♂, 3♀ (TARI), same but with “26.III.2013”; 3♂ (TARI), same locality, 2.III.2009, leg. Y.-T. Chung; 1♂, 3♀ (TARI), same but with “22.VII.2012”; 1♀ (TARI), same but with “30.VII.2012”; 1♀ (TARI), same but with “5.VIII.2012”; 1♂ (TARI), same but with “16.II.2013”; 3♂, 1♀ (TARI), same but with “26.II.2013”; 1♂ (TARI), same but with “7.III.2013”; 1♀ (TARI), same but with “14.III.2013”; 1♂, 3♀ (TARI), same but with “3.IV.2013”; 2♀ (TARI), same but with “16.IV.2013”; 1♀ (TARI), same but with “30.V.2013”; 2♀ (TARI), same but with “9.VI.2013”; 1♂ (TARI), same but with “2.VII.2013”; 1♂ (TARI), same but with “6.VIII.2013”; 1♀ (TARI), same but with “2.IX.2013”; 2♀ (TARI), same but with “23.V.2014”; 1♂, 1♀ (TARI), same but with “22.X.2014”; 1♂ (TARI), same but with “18.III.2016”; 4♂ (TARI), same but with “28.III.2016”; 5♂, 4♀ (TARI), same but with “8.IV.2016”; 2♀ (TARI), same but with “6.VIII.2016”; 4♂, 2♀ (TARI), same but with “24.III.2017”; 6♂, 1♀ (TARI), same but with “4.IV.2017”; 1♀ (TARI), same but with “10.IV.2017”; 1♀ (TARI), same but with “12.V.2017”; 1♂ (TARI), same but with “9.IV.2018”; 1♂ (TARI), same but with “26.VII.2019”; 1♂, 1♀ (TARI), same but with “10.IV.2020”; 1♂ (TARI), same but with “27.IV.2020”; 1♂ (TARI), same but with “10.IV.2021”; 1♂ (TARI), same but with “15.IV.2021”; 1♂ (TARI), same locality, 21.III.2009, leg. M.-H. Tsou; 2♀ (TARI), same but with “28.IV.2012”; 1♀ (TARI), same locality, 8.V.2009, leg. U. Ong; 2♀ (TARI), same but with “1.VIII.2009”; 3♂ (TARI), same but with “26.III.2011”; 1♂, 12♀ (TARI), same but with “23.III.2012”; 3♂ (TARI), same locality, 23.III.2010, leg. J.-C. Chen; 1♂ (TARI), same but with “30.III.2010”; 1♂ (TARI), same but with “21.VII.2010”; 1♂, 1♀ (TARI), same but with “12.VIII.2010”; 1♀ (TARI), same but with “4.X.2010”; 1♂ (TARI), same but with “16.VIII.2011”; 1♀ (TARI), same but with “10.IV.2012”; 1♀ (TARI), same but with “3.VI.2013”; 4♂, 5♀ (TARI), same locality, 14.VIII.2011, leg. Y.-T. Wang; 1♀ (TARI), same but with “26.II.2013”; 1♂ (TARI), same locality, 25.III.2013, leg. B.-X. Guo; 2♀ (TARI), same locality, 6.IV.2013, leg. W.-C. Liao; 2♂ (TARI), same but with “22.III.2015”; 1♂, 3♀ (TARI), Wutai (霧台), 12.IV.2009, leg. U. Ong; 2♀ (TARI), same but with “9.V.2009”; 1♀ (TARI), same but with “12.V.2009”; 1♀ (TARI), same but with “17.V.2009”; Taichung: 1♀ (TARI), Kukuan (谷關), 16.VII.2007, leg. M.-H. Tsou; Tainan: 1♀ (TARI), Kantoushan (崁頭山), 14.III.2010, leg. M.-H. Tsou; 3♂, 2♀ (TARI), Meiling (梅嶺), 19.IV.2014, leg. W.-C. Liao; 2♀ (TARI), same locality, 26.VII.2015, leg. U. Ong; 1♀ (TARI), same but with “22.IV.2016”; Taipei: 1♀ (TARI), Fushan (福山), 18.IV.2010, leg. M.-H. Tsou; 1♂ (BPBM), Rimogan (= Fushan, 福山) – Rahau (= Hsinhhsien, 信賢), 24.VII.1934, leg. J. L. Gressitt (identified as *A. testaceus* by Kimoto (1969)); 1♀ (TARI), Wulai (烏來), 5.III.2009, leg. Y.-L. Lin; 2♂ (TARI), same but with “5.III.2016”; Taitung: 1♂ (TARI), Ima trail (依麻林道), 10.VII.2006, leg. H.-Y. Lee; 1♀ (TARI), Lichia (利嘉), 14.VI.2011, leg. U. Ong; 2♂, 2♀ (TARI), same locality, 15.VII.2014, leg. Y.-T. Chung; 1♂ (TARI), same locality, 16.VII.2014, leg. Y.-T. Wang; 1♂ (TARI), same locality, 1.VII.2016, leg. B.-X. Guo; 1♂, 1♀ (TARI), Taimali (太麻里), 20.III.2008, leg. P.-F. Wang; 1♂, 1♀ (TARI), Tajen (達仁), 16–17.III.2009, leg. U. Ong; 1♀ (TARI), Tulan (都蘭), 4.IX.2018, leg. Y.-T. Chung; Taoyuan: 2♂, 11♀ (TARI), Fufushan (夫婦山), 5.IV.2015, leg. M.-H. Tsou; 1♀ (TARI), same but with “26.IV.2015”; 1♀ (TARI), Fuhsing (復興), 3.V.2010, leg. Y.-T. Wang; 1♂, 1♀ (TARI), Hsiaowulai (小烏來), 29.IX.2009, leg. S.-F. Yu; 2♀ (TARI), Hsuehwunao (雪霧鬧), 2.IV.2011, leg. M.-H. Tsou; 2♀ (TARI), same but with “10.IV.2011”; 1♂ (TARI), same locality, 1.IX.2012, leg. Y.-L. Lin; 1♂, 1♀ (TARI), Paling (巴陵), 4.IV.2010, leg. M.-H. Tsou; 1♂ (TARI), same but with “29.III.2015”; 1♀ (TARI), Suleng (四稜), 9.IV.2016, leg. Y.-L. Lin; 1♀ (TARI), Tungyanshan (東眼山), 14.VIII.2008, leg. H. Lee; 1♀ (TARI), same but with “21.IV.2009”; 1♀ (TARI), same but with “2.V.2009”.
